# Heteroatoms Synergistic Anchoring Vacancies in Phosphorus-Doped CoSe_2_ Enable Ultrahigh Activity and Stability in Li–S Batteries

**DOI:** 10.1007/s40820-025-01806-0

**Published:** 2025-06-23

**Authors:** Xiaoya Zhou, Wei Mao, Chengwei Ye, Qi Liang, Peng Wang, Xuebin Wang, Shaochun Tang

**Affiliations:** Key National Laboratory of Solid State Microstructures, Collaborative Innovation Center of Advanced Microstructures, Jiangsu Key Laboratory of Artificial Functional Materials, College of Engineering and Applied Sciences, Nanjing University, Nanjing, 210093 People’s Republic of China

**Keywords:** Vacancy, Heteroatomic anchoring, Vacancy migration, Activity/stability trade-off, Electrocatalysts

## Abstract

Electrocatalyst activity and stability demonstrate a “seesaw” relationship. Introducing vacancies (Vo) enhances the activity by improving reactant affinity and increasing accessible active sites. However, deficient or excessive Vo reduces polysulfide adsorption and lowers catalytic stability. Herein, a novel “heteroatoms synergistic anchoring vacancies” strategy is proposed to address the trade-off between high activity and stability. Phosphorus-doped CoSe_2_ with remained rich selenium vacancies (P-CS-Vo-0.5) was synthesized by producing abundant selenium Vo followed by controlled P atom doping. Atomic-scale microstructure analysis elucidated a dynamic process of surface vacancy generation and the subsequent partial occupation of these vacancies by P atoms. Density functional theory simulations and in situ Raman tests revealed that the Se vacancies provide highly active catalytic sites, accelerating polysulfide conversion, while P incorporation effectively reduces the surface energy of Se vacancies and suppresses their inward migration, enhancing structural robustness. The battery with the optimal P-CS-Vo-0.5 separator delivers an initial discharge capacity of 1306.7 mAh g^−1^ at 0.2C, and maintain 5.04 mAh cm^−2^ at a high sulfur loading (5.7 mg cm^−2^, 5.0 μL mg^−1^), achieving 95.1% capacity retention after 80 cycles. This strategy of modifying local atomic environments offers a new route to designing highly active and stable catalysts.

**Supplementary Information:**

The online version contains supplementary material available at 10.1007/s40820-025-01806-0.

## Highlights

- A volcano-type relationship between catalytic activity and vacancy concentration is revealed based on systematic investigation on selenium-vacancy-rich CoSe_2_.
- A novel “heteroatoms synergistic anchoring vacancies” tactic is firstly proposed to achieve P-doped CoSe_2_ with remained rich selenium vacancies (P-CS-Vo-0.5).
- It has been demonstrated that P doping lowers Se vacancy surface energy and effectively “pins” active sites, markedly suppressing dynamic migration of vacancies.

**Supplementary Information:**

The online version contains supplementary material available at 10.1007/s40820-025-01806-0.

## Graphical abstract

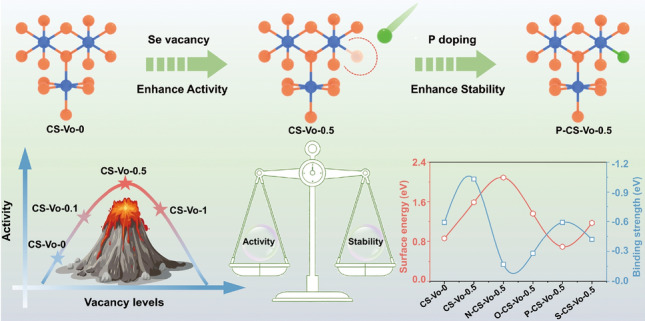

## Introduction

Lithium-sulfur batteries (LSBs) have been considered promising candidates for next-generation energy storage due to high theoretical energy density (2600 Wh kg^−1^) and capacity (1675 mAh g^−1^) [1, 2]. The shuttle effect and slow sulfur conversion kinetics result in low sulfur utilization and rapid capacity decay, seriously hindering the practical application of LSBs [3, 4]. Polar catalysts (e.g., materials featuring polar groups or unsaturated coordination metal centers) mitigate the shuttle effect and enhance sulfur utilization by facilitating lithium polysulfide (LiPSs) conversion, modulating adsorption behavior, and improving ion transport [5, 6]. Cobalt selenide (e.g., CoSe_2_) is widely used in catalysis due to its excellent physicochemical properties, and its overall performance surpasses that of other metal selenides, such as FeSe_2_, NiSe_2_, and MoSe_2_ [6]. Firstly, CoSe_2_ exhibits strong adsorption of LiPSs because the d-orbital electrons of cobalt, especially in the eg orbital, form strong covalent bonds with sulfur atoms, effectively suppressing the shuttle effect. Secondly, CoSe_2_ has a high electrical conductivity (around 10^3^ S cm^−1^), which is much higher than that of semiconducting materials like FeSe_2_ and MoSe_2_, facilitating electron transport and enhancing catalytic efficiency. Additionally, CoSe_2_ features a stable cubic octahedral structure that can accommodate the volume changes of the sulfur cathode during charge–discharge cycles, maintaining structural stability. However, insufficient active site density represents a critical bottleneck limiting catalytic performance enhancement and hindering substantial improvements in efficiency.

Vacancy defect engineering has emerged as an effective strategy for enhancing catalytic activity. Due to the coordinative unsaturation of neighboring atoms, Vo serve as highly active centers promoting strong interactions with reactant molecules [7, 8]. Furthermore, Vo indirectly modulate the electronic structure by introducing defect states and altering the band structure, thereby facilitating reactant adsorption and activation [9, 10]. However, the role of Vo is a double-edged sword: insufficient vacancy concentration limits active sites, hindering effective catalysis, while excessive concentration compromises structural integrity, leading to active site collapse and rapid electrode degradation [11]. Shao et al. demonstrated that a sulfur vacancy concentration below 8% in MoS_2_ catalysts diminishes the adsorption of LiPSs, consequently reducing catalytic performance [12]. Gao et al. synthesized WSe_1.51_ with the best polysulfide binding and catalysis, and demonstrated that excessive defects (e.g., W: Se = 1.33) led to structural collapse and significant decrease in activity [13]. Therefore, precise control of vacancy concentration is essential [12]. In addition, even with optimized initial vacancy concentrations, these ‘bare’ Vo often exhibit instability under harsh reaction conditions, prone to migration, aggregation, or even annihilation, ultimately leading to diminished catalytic performance [14, 15]. Maintaining high activity while suppressing the dynamic migration of Vo to enhance stability remains a significant challenge.

Here, we proposed a novel “heteroatoms synergistic anchoring vacancies” strategy to obtain phosphorus-doped CoSe_2_ containing rich selenium vacancies (P-CS-Vo-0.5) with high catalytic activity and stability. Modulating the NaBH_4_ concentration enabled precise control over vacancy levels, revealing a volcano-type relationship between these vacancies and catalytic activity. Phosphorus doping addressed the stability issue of highly active catalysts by lowering Se vacancy surface energy, anchoring active sites, and inhibiting vacancy migration. Benefiting from the synergistic effects of vacancy control and heteroatom anchoring, the battery with optimal P-CS-Vo-0.5 separator demonstrated exceptional electrochemical performance, including a high sulfur utilization of 1306.7 mAh g^−1^ at 0.2C and a remarkable cyclic stability. This work not only elucidates the deactivation mechanism of Se-vacancy catalysts at the atomic level but also establishes a general strategy for stabilizing active sites via doping engineering, paving the way for the development of long-life, high-performance energy storage devices.

## Experimental Section

### Preparation of ZIF-67 and TA-ZIF-67

Firstly, 300 mg of cobalt nitrate hexahydrate (Co(NO_3_)_2_·6H_2_O) and 15 mg of cetyltrimethyl ammonium bromide (CTAB) were added into 20 mL of deionized water (DI) and stirred to obtain a transparent pink solution (solution A); 2-methylimidazole with a weight of 908 mg was added to 140 mL DI and stirred for a while to obtain a transparent solution B. Then pour solution A quickly into solution B and stir vigorously for 40 min to get a purple suspension. The resulting precipitate was washed six times with ethanol and dried under vacuum at 60 °C overnight to obtain ZIF-67. Afterward, the as-prepared ZIF-67 was dispersed into 20 mL of ethanol and then poured into 180 mL of a mixed solution of ethanol and deionized water (VH_2_O:Vethanol = 1:1) containing tannic acid (TA, 1 mg mL^−1^) and stirred for 15 min at room temperature. The products were collected by centrifugation, washed several times with ethanol and denoted as TA-ZIF-67.

### Preparation of CoSe_2_

The TA-ZIF-67 powder and selenium powder (mass ratio 1:3) were put into a crucible and heated at 500 °C for 2 h under the protection of H_2_/Ar (at a heating rate of 2 °C min^−1^) to obtain CoSe_2_. The prepared CoSe_2_ (50 mg) was soaked in 0.5 mol L^−1^ NaBH_4_ solution for 30 min, then washed with deionized water, and dried overnight at 60 °C to obtain target material (CS-Vo-0.5).

### Preparation of P-CS-Vo-0.5

NaH_2_PO_2_ (2 g) and CS-Vo-0.5 (100 mg) were placed in the upstream and downstream of the tubular furnace, respectively, and heated at 350 °C for 2 h under an Ar atmosphere to obtain P-CS-Vo-0.5.

More details of other syntheses and characterizations can be seen in Supporting Information.

## Results and Discussion

### Construction of Vacancies and Screening of Heteroatomic Stable Vacancies

To elucidate the effect of varying vacancy levels on the catalytic conversion of LiPSs, we synthesized a series of selenium-vacancy-rich CoSe_2_ (CS) catalysts using NaBH_4_ treatment at varying concentrations (CS-Vo-M, M = NaBH_4_ concentration: 0; 0.1; 0.5; 1 mol L^−1^) (Figs. S1 and S2). The reactive hydrogen species (H⁻/H·) released from NaBH_4_ preferentially attack Se^2−^ in the CoSe_2_ lattice, reducing selenium atoms to H_2_Se and thereby creating selenium vacancies within the lattice. Simultaneous electron injection induces a reduction in the cobalt oxidation state, resulting in the breaking of Se-Co bonds and the extraction of selenium atoms, ultimately leading to the formation of stable vacancy defects. In addition, inductively coupled plasma optical emission spectroscopy (ICP-OES) analysis showed that the stoichiometric ratios of the metal elements deviated from the theoretical values (Table S1), confirming the differences in vacancy concentrations from the perspective of elemental composition.

**Fig. 1:**
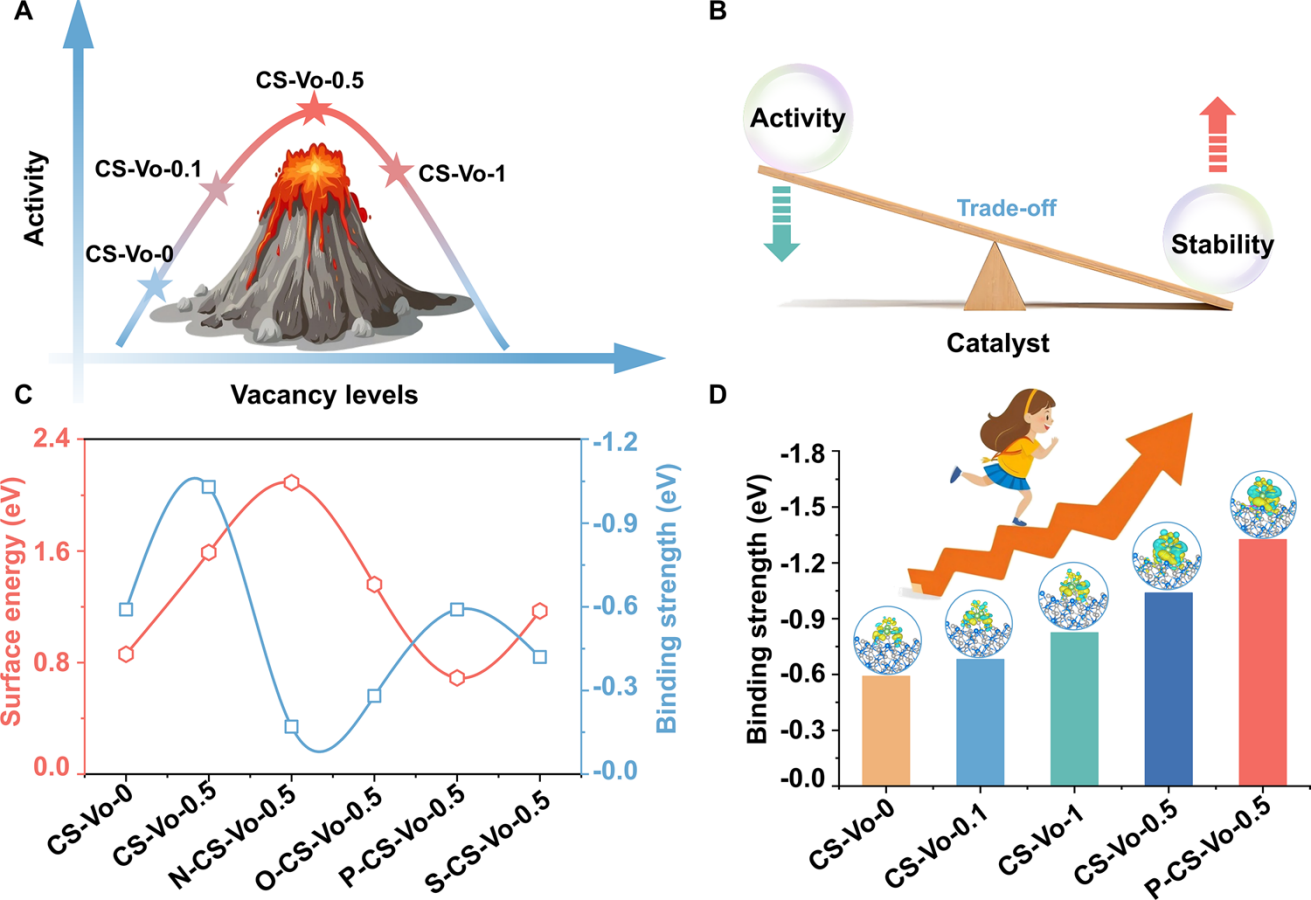
**A** Relationship between vacancy levels and catalytic activity; **B** seesaw relationship between catalyst activity and stability; **C** calculated binding strength to Li_2_S_4_ and surface energy of N-CS-Vo-0.5, S-CS-Vo-0.5, O-CS-Vo-0.5, P-CS-Vo-0.5; **D** Adsorption energy and differential charge density of the Li_2_S_4_ on the catalyst

Density functional theory (DFT) simulations disclosed a nonlinear relationship between the polysulfide adsorption energy and the concentration of vacancies. As the vacancy concentration increased, the adsorption strength first increased and then decreased, resulting in a volcano-shaped adsorption profile (Figs. 1A and S3-S7). Low vacancy concentrations hinder the formation of sufficient active sites, while high vacancy concentrations led to significant geometric damage or reduced activity [12]. Consequently, the CS-Vo-0.5 catalyst exhibited superior electrochemical activity. The augmented catalytic performance was predominantly attributed to the surface or subsurface vacancies serving as active sites during surface chemical reactions. The formation energy of CS-Vo-0.5 remained at a relatively high level, with the formation of internal vacancies (E_Int-1_ = 2.07 eV, E_Int-2_ = 2.16 eV) being lower than that of surface vacancies (E_Sur-1_ = 2.71 eV, E_Sur-2_ = 2.59 eV) (Fig. S8). The inward migration of these vacancies leaded to a decline in catalytic performance during cycling [16, 17].

An intrinsic seesaw relationship exists between catalyst activity and stability (Fig. 1B). Modifying the catalyst with non-metal atoms to stabilize vacancies represents a promising approach to reconciling this inherent conflict. The incorporation of different dopant elements markedly alters the surface and binding energies of the material, primarily through the synergistic modulation of the electronic structure and lattice strain by their electronegativity and atomic radius. The surface energy of CS-Vo-0 was 0.86 eV, which increased to 1.59 eV upon vacancy formation (CS-Vo-0.5), indicating an inherent instability of vacancy-laden surfaces. Optimized models of N-CS-Vo-0.5, S-CS-Vo-0.5, O-CS-Vo-0.5, and P-CS-Vo-0.5 were used to determine binding energies and surface energies (Figs. 1C and S9). The results show that the surface energy exhibits an initial increase followed by a decrease upon doping, primarily due to the extent of lattice distortion induced by the dopants. Nitrogen, with its high electronegativity (3.04) and small atomic radius (0.71 Å), tends to cause lattice contraction, leading to a significant increase in surface energy. In contrast, phosphorus possesses a moderate electronegativity (2.19) and an atomic radius (1.07 Å) close to that of Se (1.16 Å), which allows it to alleviate lattice strain while optimizing the electronic structure, resulting in the lowest surface energy for the P-CS-Vo-0.5 configuration. Oxygen and sulfur exhibit a mismatch between electronegativity and atomic radius, yielding intermediate modulation effects. Binding energy analysis further reveals that phosphorus doping not only reduces the surface energy (0.69 eV) but also significantly enhances the vacancy binding energy (− 0.59 eV), thereby improving structural stability. This behavior is closely associated with the efficiency of interfacial charge transfer. Although nitrogen and oxygen enhance interfacial polarization due to their high electronegativity, excessive polarization hinders effective electron transfer, resulting in reduced binding energy. In contrast, the moderate electronegativity of phosphorus facilitates electron delocalization, thereby enhancing interfacial adsorption and structural stability. Furthermore, incorporating P effectively anchored the vacancies, thereby preventing their inward migration (Fig. S10). To elucidate the roles of vacancies and P modification in the adsorption of LiPSs, we investigated the charge density differences of the CS-Vo-0, CS-Vo-0.5, and P-CS-Vo-0.5 adsorption systems (Fig. 1D). The binding energy of P-CS-Vo-0.5 with Li_2_S_4_ (− 1.326 eV) surpassed that of CS-Vo-0 (− 0.591 eV) and CS-Vo-0.5 (− 1.039 eV), indicating enhanced chemical adsorption capabilities of P-CS-Vo-0.5, which effectively captured LiPSs. Consequently, to balance the adsorption and catalytic activity of CS-Vo-0.5, the P atom was selected as a dopant to optimize the trade-off between the activity and stability of CS-Vo-0.5, warranting further investigation [18].

### Morphological and Structural Characterizations

**Fig. 2:**
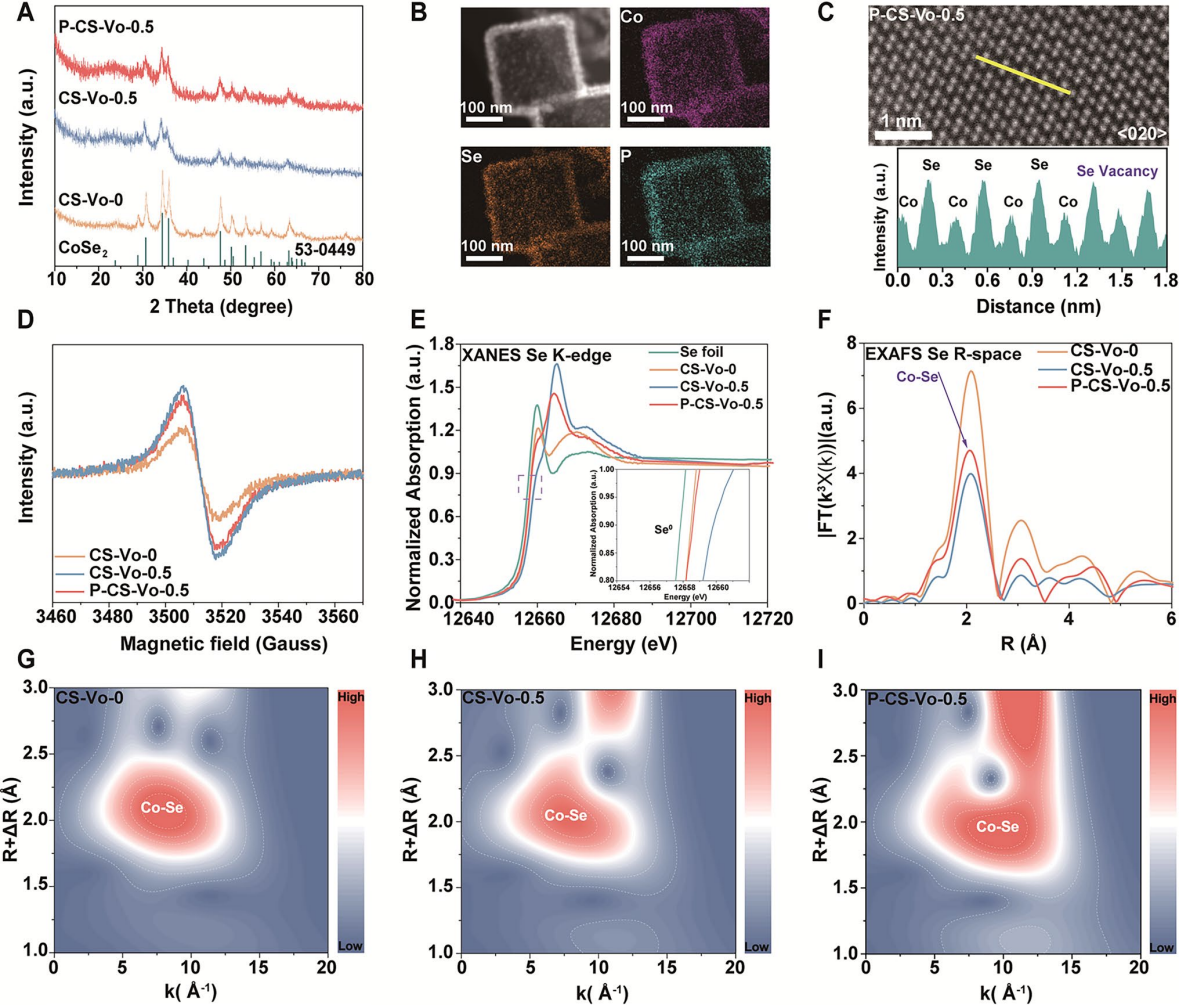
**A** XRD patterns; **B** Scanning TEM-high-angle annular dark-field (STEM-HADDF) image, and mapping images of P-CS-Vo-0.5; **C** AC-HADDF-STEM image and line-scanning intensity profile acquired from the yellow line; **D** Electron paramagnetic resonance (EPR) spectra; **E–F** Se K-edge XANES spectra and corresponding FT-EXAFS spectra; **G-I** Wavelet transform contour plots of Se K-edge in CS-Vo-0, CS-Vo-0.5, P-CS-Vo-0.5

The borohydride-induced vacancies enhanced the activity of the catalyst, while P atom incorporation enhanced stability [11]. DFT calculations revealed that P preferentially fills Se vacancies within the CS-Vo-0.5 lattice, forming a more stable configuration compared to substitutional doping (Figs. S11 and S12). X-ray diffraction (XRD) patterns of CS-Vo-0, CS-Vo-0.5, and P-CS-Vo-0.5, shown in Fig. 2A, revealed no characteristic peaks of cobalt phosphide in P-CS-Vo-0.5, suggesting that the incorporation of phosphorus did not disrupt the crystal structure [11]. Transmission electron microscopy (TEM) images illustrated that P-CS-Vo-0.5 retained a hollow cubic morphology (Fig. S13), affirming that its structure remained unchanged after NaBH_4_ treatment. Elemental mapping demonstrated the uniform distribution of Co, Se, and P elements in the P-CS-Vo-0.5 sample (Figs. 2B and S14-S16). The high-resolution TEM (HRTEM) image of P-CS-Vo-0.5 exhibited regular crystal fringes (Fig. S17).

Spherical aberration-corrected HADDF-STEM (AC-HADDF-STEM) observations were carried out on CS-Vo-0, CS-Vo-0.5, and P-CS-Vo-0.5 to observe the Se vacancy. Among them, in agreement with the theoretical model, the atomic columns of Co and Se could be observed along the < 020 > zone axis for CS-Vo-0.5 and along the < 022 > zone axis for P-CS-Vo-0.5, respectively. The strength of the designated Se site was much weaker than that of other Se sites, as could be seen from the examination of Figs. 2C and S18, which supported the presence of selenium vacancies. The change of valence states of metal elements was shown by EELS, as depicted in Fig. S19. Compared with CS-Vo-0, the L3/L2 ratio of CS-Vo-0.5 slightly increased, mainly due to the decreased valence state of cobalt after vacancy formation, leading to an increased electron cloud density around it. Conversely, the L3/L2 ratio of P-CS-Vo-0.5 slightly decreased, primarily because phosphorus filled the vacancies, reducing the vacancy concentration and thus decreasing the surrounding electron cloud density. Electron paramagnetic resonance (EPR) measurements further probed the evolution of vacancy, as shown in Figs. 2D and S20. The EPR signal of CS-Vo-0.5 (spins = 4.625 × 10^17^) was higher than that of CS-Vo-0 (spins = 3.062 × 10^17^), suggesting that the treatment with sodium borohydride increased the vacancy concentration. The EPR signal of P-CS-Vo-0.5 was lower (spins = 4.016 × 10^17^), suggesting that P atom filling lowered the vacancy concentration. The BET specific surface area and pore volume of the electrode material were further studied (Fig. S21). Compared with CS-Vo-0, the surface area and pore volume of CS-Vo-0.5 and P-CS-Vo-0.5 were improved after NaBH_4_ treatment. These improvements in surface area and pore volume may be caused by vacancies created in CS-Vo-0. Notably, the P-CS-Vo-0.5 separator possessed a uniform thickness (15 μm) and enhanced wettability, which facilitated rapid electrolyte penetration (Figs. S22 and S23).

X-ray photoelectron spectroscopy (XPS) was used to further investigate the chemical state and local atomic structure of CS-Vo-0, CS-Vo-0.5, and P-CS-Vo-0.5. In the high-resolution Co 2*p* XPS spectra of P-CS-Vo-0.5 (Fig. S24A), the Co 2*p*_1/2_ and Co 2*p*_3/2_ peaks were located at 797.2 and 781.2 eV, respectively. Meanwhile, two peaks at 793.2 eV (Co) and 778.3 eV (Co 2*p*_3/2_) could be attributed to partial surface oxidation of the sample in air [11]. Compared to CS-Vo-0 (780.6 eV), the 2*p*_3/2_ peak of CS-Vo-0.5 (780.4 eV) was shifted to a lower binding energy due to Se vacancies. The Se 3*d*_5/2_ and 3*d*_3/2_ peaks of P-CS-Vo-0.5 were centered at 54.98 and 55.78 eV, respectively (Fig. S24B). In comparison with CS-Vo-0.5, the Se 3*d*_5/2_ and 3*d*_3/2_ peaks of P-CS-Vo-0.5 were shifted to lower binding energy, indicating the enhanced electron density of Se due to the additional P doping [19]. The P 2*p* spectra showed two broad peaks, which could be further deconvoluted into P-O, P 2*p*_3/2_, and P 2*p*_1/2_ bonds at 138.18, 133.58, and 134.58 eV (Fig. S24C). The XPS results demonstrated that the incorporation of P into the structure of CS-Vo-0.5 enhances the modulation of the electronic structure [8].

X-ray absorption near-side structure spectra (XANES) and extended X-ray absorption fine structure spectra (EXAFS) were acquired to investigate the local structure of Se atoms in more detail. Figure 2E displays the Se K-edge XANES spectra of CS-Vo-0, CS-Vo-0.5, P-CS-Vo-0.5, and Se foil. Compared to the CS and Se foils, the absorption edge of the Se K-edge in CS-Vo-0.5 moved toward higher energies, indicating a decrease in electron density on the Se atom due to the Se vacancy. At the same time, the absorption edge of P-CS-Vo-0.5 moved slightly in the direction of lower energy than that of CS-Vo-0.5, indicating that the average electron density around Se increased due to the additional doping of the P atom [20].The XANES results were in good agreement with the above XPS results. To better understand the detailed local geometric coordination environment of the sample, we obtained the Fourier transforms corresponding to the k3-weighted EXAFS oscillations (FT-EXAFS) of CS-Vo-0, CS-Vo-0.5, P-CS-Vo-0.5, and Se foils (Fig. 2F). The central peak of the Co-Se bond appeared in CS-Vo-0, CS-Vo-0.5, and P-CS-Vo-0.5 samples at ≈2.1 Å. Compared with CS-Vo-0, the peak intensity of the unique shell scattering of the Co-Se bond of CS-Vo-0.5 was significantly reduced, which indicated that the coordination number of Co and Se was lower, and the presence of Se vacancy led to the increase of material disorder. Notably, P-CS-Vo-0.5 had a higher peak strength than CS-Vo-0.5, which meant that the P atoms filled the vacancy, reducing the disorder of the material. Prominent peaks in the Fourier transform may mask weak peaks, resulting in the loss of weak peaks [20, 21]. Therefore, using wavelet transform (WT) EXAFS and contour plots, the structural disorder and electronic structure of CS-Vo-0, CS-Vo-0.5, and P-CS-Vo-0.5 could be further distinguished (Fig. 2G-I). Maximum intensity was observed in the wavelet transform analysis and was related to the Co-Se bond. Compared with CS-Vo-0 and P-CS-Vo-0.5, a recognizable negative displacement of radial distance was shown in CS-Vo-0.5, indicating that the vacancy generation caused the bond length change of Co-Se [22, 23].

### Chemisorption and Electrocatalytic Performance

**Fig. 3:**
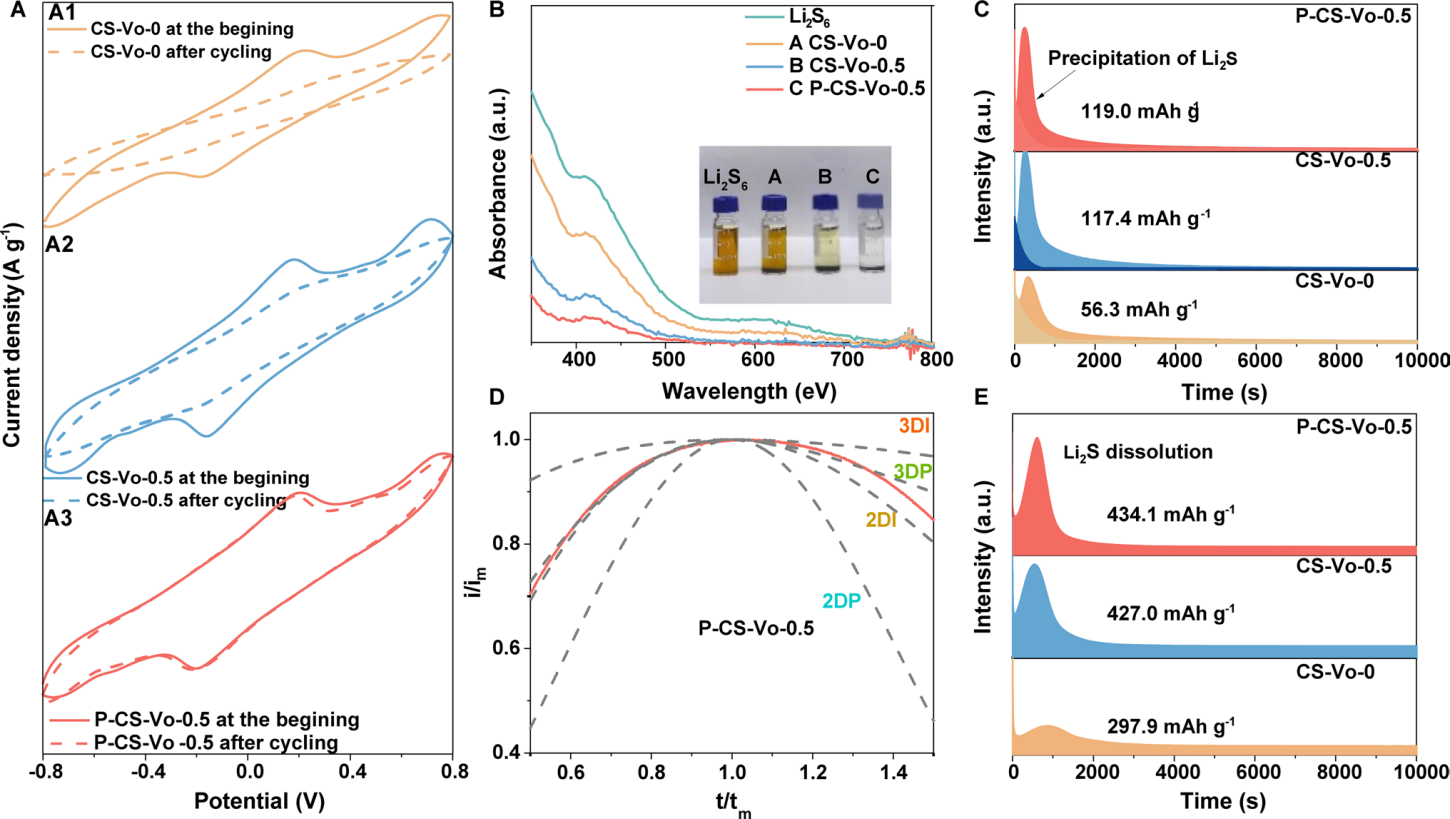
**A** CV curves of the symmetric cells at the beginning and after cycling; **B** Photographs of the Li_2_S_6_ adsorption and the UV–vis spectra; **C** Nucleation process; **D** Corresponding dimensionless transient curves of P-CS-Vo-0.5; **E** Dissociation process of Li_2_S with different catalysts

To evaluate the catalytic activity and stability, cyclic voltammetry (CV) measurements were performed using symmetric cells (Fig. 3A). In the first cycle, the CV peak current and peak area of CS-Vo-0.5 increased significantly compared with those of CS-Vo-0, indicating that the abundant vacancies in CS-Vo-0.5 enhance its catalytic performance (Fig. 3A1-2) . However, after 100 cycles, CS-Vo-0.5 exhibited a sharp decrease in peak current and peak area relative to the initial cycle, and the redox peaks disappeared, suggesting a decline in cycling stability. The CV curves of the symmetric cell with the P-CS-Vo-0.5 electrode were shown in Fig. 3A3. Compared with CS-Vo-0 and CS-Vo-0.5, P-CS-Vo-0.5 displayed a higher initial current density and a larger peak area, indicating superior catalytic performance. Moreover, after 100 cycles, the peak current and peak area of the P-CS-Vo-0.5 symmetric cell remained stable, demonstrating excellent catalytic stability. These results suggested that the catalytic stability of CS-Vo-0.5 could be effectively enhanced through P modification. The adsorption capacity of catalysts for polysulfides played a crucial role in mitigating the shuttle effect.

Visual adsorption tests and UV–vis spectroscopy were conducted using Li_2_S_6_ solutions (Fig. 3B). While the solutions in the other three systems remained pale yellow or yellow, the Li_2_S_6_ solution in the P-CS-Vo-0.5 turned clear and colorless after 12 h, indicating strong chemical interactions between P-CS-Vo-0.5 and LiPSs. This result was in line with the UV–vis absorption spectrum. To further evaluate the catalytic effects of different electrocatalysts on the reduction and oxidation processes, Li_2_S precipitation and dissolution experiments were performed (Fig. 3C). The battery with P-CS-Vo-0.5 separator exhibited higher electrocatalytic activity for Li_2_S nucleation, with a nucleation capacity (119.0 mAh g^−1^) surpassing that of CS-Vo-0.5 (117.4 mAh g^−1^) and CS-Vo-0 (56.3 mAh g^−1^). The Li_2_S deposition on the P-CS-Vo-0.5 catalyst followed a 3D pattern, indicative of superior nucleation and growth rates (Fig. S25) [24]. During the Li_2_S dissolution process, the decomposition capacity of P-CS-Vo-0.5 (434.1 mA h g^−1^) far exceeded that of CS-Vo-0.5 (427.0 mAh g^−1^) and CS-Vo-0 (297.9 mA h g^−1^) (Fig. 3D). These results suggested that doping and vacancy control could significantly enhance electrochemical performance by improving the redox kinetics associated with Li_2_S nucleation and dissociation [25].

**Fig. 4:**
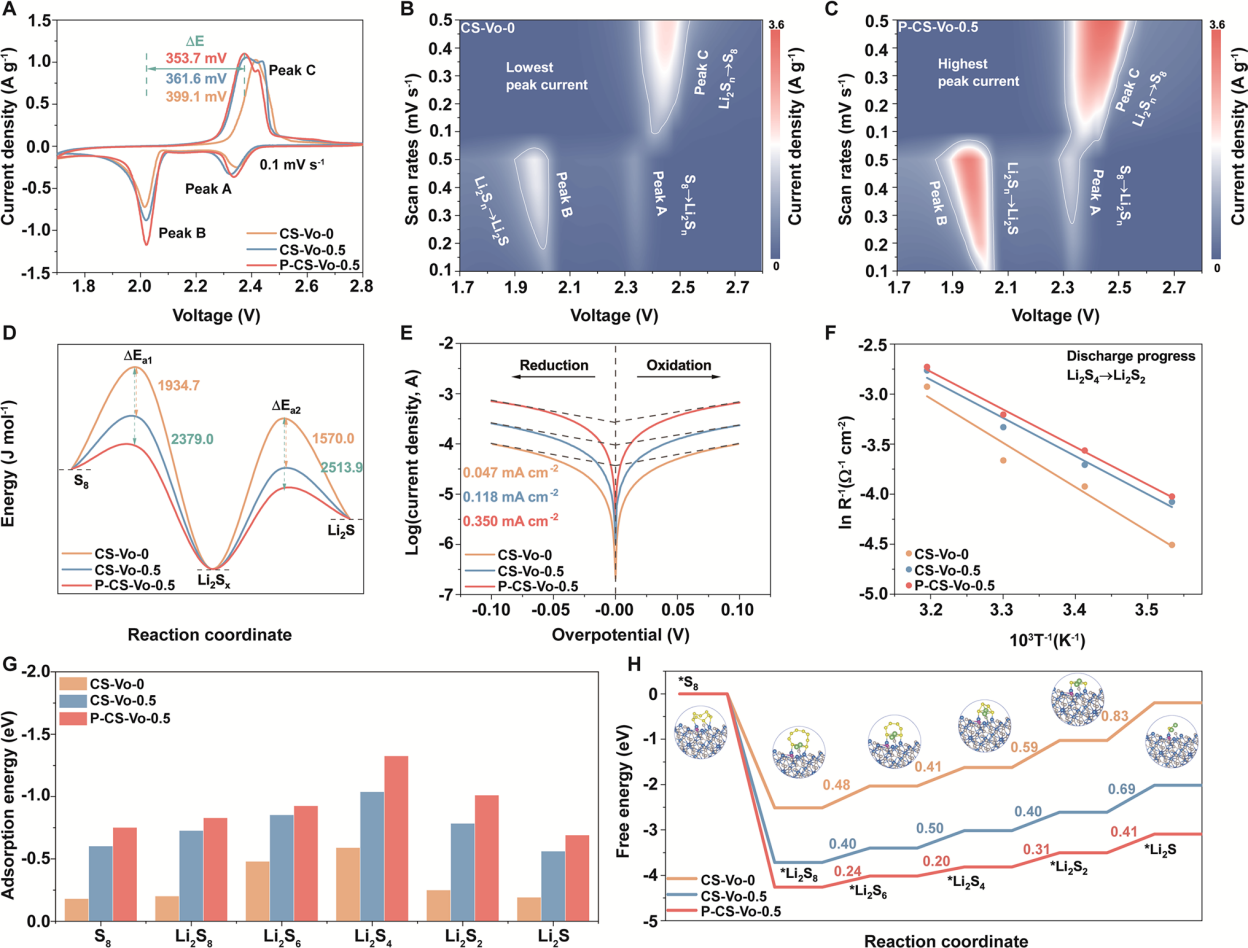
**A** CV curves; Contour plots of CV patterns for **B** CS-Vo-0 and **C** P-CS-Vo-0.5 with different scan rates; **D** Relative activation energies of the two sulfur cathodes; **E** Tafel curves; **F** Linear relationship between the inverse of absolute temperature (*T*) and the logarithm of the reciprocal of the charge-transfer resistance; **G** Calculated adsorption energy of sulfur species, and **H** relative free energy

The batteries were assembled using separators modified with different catalysts to evaluate their electrochemical performance (Fig. S26). As shown in Fig. 4A, compared with CS-Vo-0 and CS-Vo-0.5, the batteries equipped with P-CS-Vo-0.5 separator exhibited notably greater redox current responsiveness, effectively reduced electrochemical polarization, and improved catalytic activity. Furthermore, the Tafel slopes of P-CS-Vo-0.5 were notably smaller than those of CS-Vo-0 and CS-Vo-0.5, suggesting the rapid conversion reaction kinetics of polysulfides on P-CS-Vo-0.5 (Fig. S27) [26]. Figures 4B, C and S28 depicts the contour plots of the CV curves at varying scan rates where P-CS-Vo-0.5 demonstrated the lowest polarization potential and larger redox peak currents compared to CS-Vo-0 and CS-Vo-0.5, further suggesting accelerated reaction kinetics. In addition, P-CS-Vo-0.5 exhibited enhanced lithium ion diffusion properties and a superior lithium-ion transfer rate (Figs. S29-S32). As demonstrated in Fig. 4D, the activation energies (ΔEa) for polysulfide conversion in these sulfur cathodes were calculated from the intercept and slope of the corresponding Tafel curves. For the reduction process of S_8_ to Li_2_S_x_, the ΔE_a1_ values of P-CS-Vo-0.5 and CS-Vo-0.5 were decreased by 2379.0 and 1934.7 J mol^−1^, respectively, compared to CS. Similarly, for the conversion of Li_2_Sₓ to Li_2_S, the ΔEa_2_ values for P-CS-Vo-0.5 and CS-Vo-0.5 were significantly lowered by 2513.9 and 1570.0 J mol^−1^, respectively. These results indicated that the energy barriers for polysulfide and Li_2_S formation were substantially reduced due to the synergistic effects of vacancy and phosphorus atom doping [27, 28].

In Fig. 4E, Tafel curves were obtained in the overpotential range of − 100 to 100 mV. Compared to CS-Vo-0.5 (0.118 mA cm^−2^) and CS-Vo-0 (0.047 mA cm^−2^), P-CS-Vo-0.5 exhibited the highest exchange current density (0.350 mA cm^−2^), indicating its superior catalytic capability in accelerating LiPSs conversion kinetics by enhancing the intrinsic electron transfer rate between the electrode and the electrolyte. To evaluate charge transfer resistance, measurements were conducted at 2.0 V, corresponding to the rate-determining step (Li_2_S_4_ to Li_2_S_2_). Based on the electrochemical impedance at different temperatures, the Ea was calculated using the Arrhenius equation (Fig. S33). The battery with the P-CS-Vo-0.5 separator exhibited the lowest Ea (0.325 eV), which was lower than that of the CS-Vo-0 (0.383 eV) and CS-Vo-0.5 (0.330 eV) separator batteries (Fig. 4F and S34). The above results indicated that P-CS-Vo-0.5 significantly reduced the activation energy for LiPSs conversion, thereby accelerating the reaction kinetics of LiPSs [29].

DFT simulations were employed to further elucidate the atomic-level interactions of sulfur species on the surfaces of P-CS-Vo-0.5, CS-Vo-0.5, and CS-Vo-0 (Fig. S35). As demonstrated in Fig. 4G, the adsorption energies of Li_2_S_8_, Li_2_S_6_, Li_2_S_4_, Li_2_S_2_, and Li_2_S on P-CS-Vo-0.5 were higher than those on CS-Vo-0.5, and CS-Vo-0, further proving the superior adsorption ability of P-CS-Vo-0.5, in agreement with the previous visualization results. The improvement in reaction kinetics was corroborated by the calculation of Gibbs free energies (Fig. 4H). The following five reduction steps, from Li_2_S_8_ to Li_2_S, were the endothermic reactions, while the last two steps were the rate-determining step. The energy barrier values of rate-limiting steps for the P-CS-Vo-0.5 surface were 0.31 and 0.41 eV, respectively, indicating that the reduction process on the P-CS-Vo-0.5 surface occurred with relative ease [30, 31].

### In Situ Characterizations

**Fig. 5:**
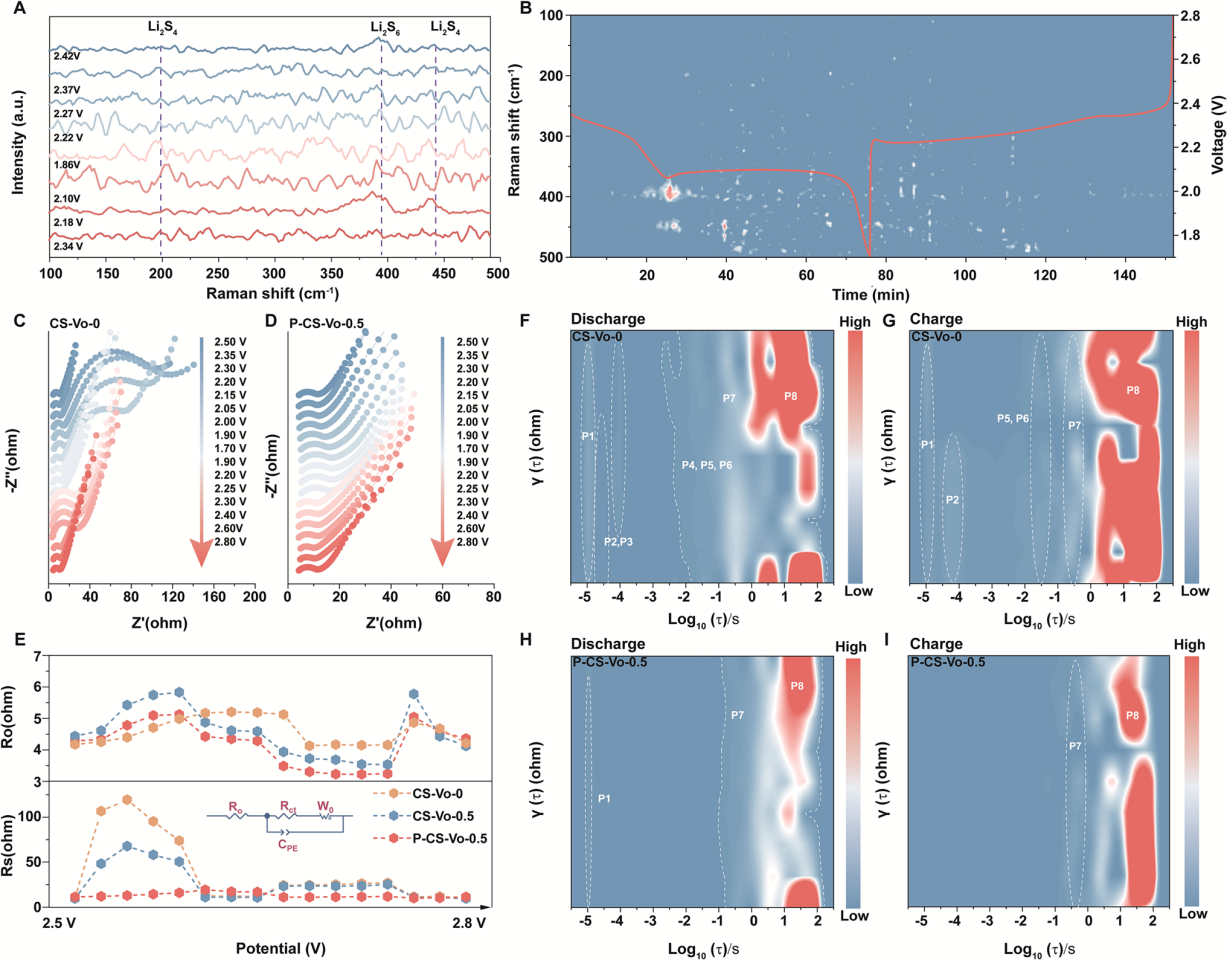
**A-B** In situ Raman spectra and corresponding Raman curves measured at different voltages during discharging/charging process of battery with P-CS-Vo-0.5 separator; **C-D** In situ electrochemical impedance spectroscopy (EIS) Nyquist plots of battery with CS-Vo-0 and P-CS-Vo-0.5 separator at 0.2C and **E** fitting results of Ro, Rs in different depths of discharge and charge. DRT contour plots were calculated from EIS measurements at different voltages of **F-G** CS-Vo-0 and **H-I** P-CS-Vo-0.5

By utilizing in situ Raman spectroscopy, we tracked the dynamic changes of LiPSs during the whole electrochemical process. As shown in Fig. 5A, B, only weak signals corresponding to S_6_^2–^ and S_4_^2–^ were observed in the battery employing the P-CS-Vo-0.5 separator during discharge and charge cycles. This suggested that the design featuring dual active sites of vacancies and P dopants significantly enhanced the affinity and catalytic activity toward LiPSs, thereby effectively mitigating the shuttle effect [25, 32]. The change of the resistance of the battery reflects the dynamic changes of the charge transfer ability. In situ EIS was employed to assess the ion transfer kinetics and to gain further insights into the charge carrier dynamics at the electrode/electrolyte interface [33, 34]. Notably, as shown in Figs. 5C-E and S36a, batteries incorporating CS-Vo-0.5 and CS-Vo-0 separators exhibited relatively high impedance during discharge. This could be primarily attributed to the conversion of sulfur into LiPSs dissolved in the electrolyte, which increased the electrolyte viscosity and leaded to a significant rise in charge transfer resistance [35, 36]. During the charging process, the charge transfer resistance initially decreased and then increased, a phenomenon attributed to the oxidation of Li_2_S and the regeneration of insulating sulfur. The initial increase and subsequent decrease in R₀ during in situ EIS arises from the dynamic dissolution–precipitation of polysulfides, which first increases electrolyte viscosity and hinders ion transport, then reduces viscosity and restores conductivity as insoluble species precipitate and Li⁺ concentration stabilizes [37, 38]. In contrast, the battery equipped with the P-CS-Vo-0.5 separator displayed the lowest and most stable impedance throughout the entire charge–discharge cycle, indicating accelerated ion transport and superior interfacial stability. This observation further highlighted the high catalytic activity of P-CS-Vo-0.5 in facilitating polysulfide conversion, underscoring its effectiveness in enhancing reaction kinetics [39].

To further correlate the time constant in impedance measurements with detailed electrochemical behavior, we further analyzed the In situ EIS using relaxation times (DRT) methods (Figs. 5F-I and S36b, c). The peaks P1, P2, and P3 in the high-frequency region corresponded to the ohmic resistance, double-layer relaxation, and lithium-ion migration processes, respectively. The peaks P4, P5, and P6 in the mid-frequency region represented the charge transfer resistance at the positive electrode, while the low-frequency regions P7 and P8 constituted the diffusion process (Fig. S37) [39]. During discharge, the CS-Vo-0 and CS-Vo-0.5 separators exhibited P1-P8 peaks, attributed to the generation of LiPSs during sulfur reduction and the hindered liquid-to-solid conversion. As discharge progresses, the ongoing dissolution of polysulfides increased electrolyte viscosity, significantly impeding diffusion processes and maintaining high diffusion resistance at P7 and P8. In contrast, the battery with the P-CS-Vo-0.5 separator maintained a stable, low impedance, reflecting its high catalytic activity in promoting polysulfide conversion, consistent with DFT calculations. The deposition of insulating Li_2_S late in discharge affects charge transfer, but P-CS-Vo-0.5 demonstrated a faster charge transfer rate, mitigating the impact of Li_2_S deposition. During charging, the continuous oxidation of Li_2_S and the rise in electrolyte viscosity were accompanied by lower diffusion resistance in P-CS-Vo-0.5, aligning with electrochemical tests. Overall, the dissolution of polysulfides and deposition of Li_2_S drove the evolution of diffusion behavior, and the combination of DRT analysis and in situ EIS provided further insights into the liquid–solid conversion kinetics.

### Electrochemical Performances of the Li–S Battery

**Fig. 6:**
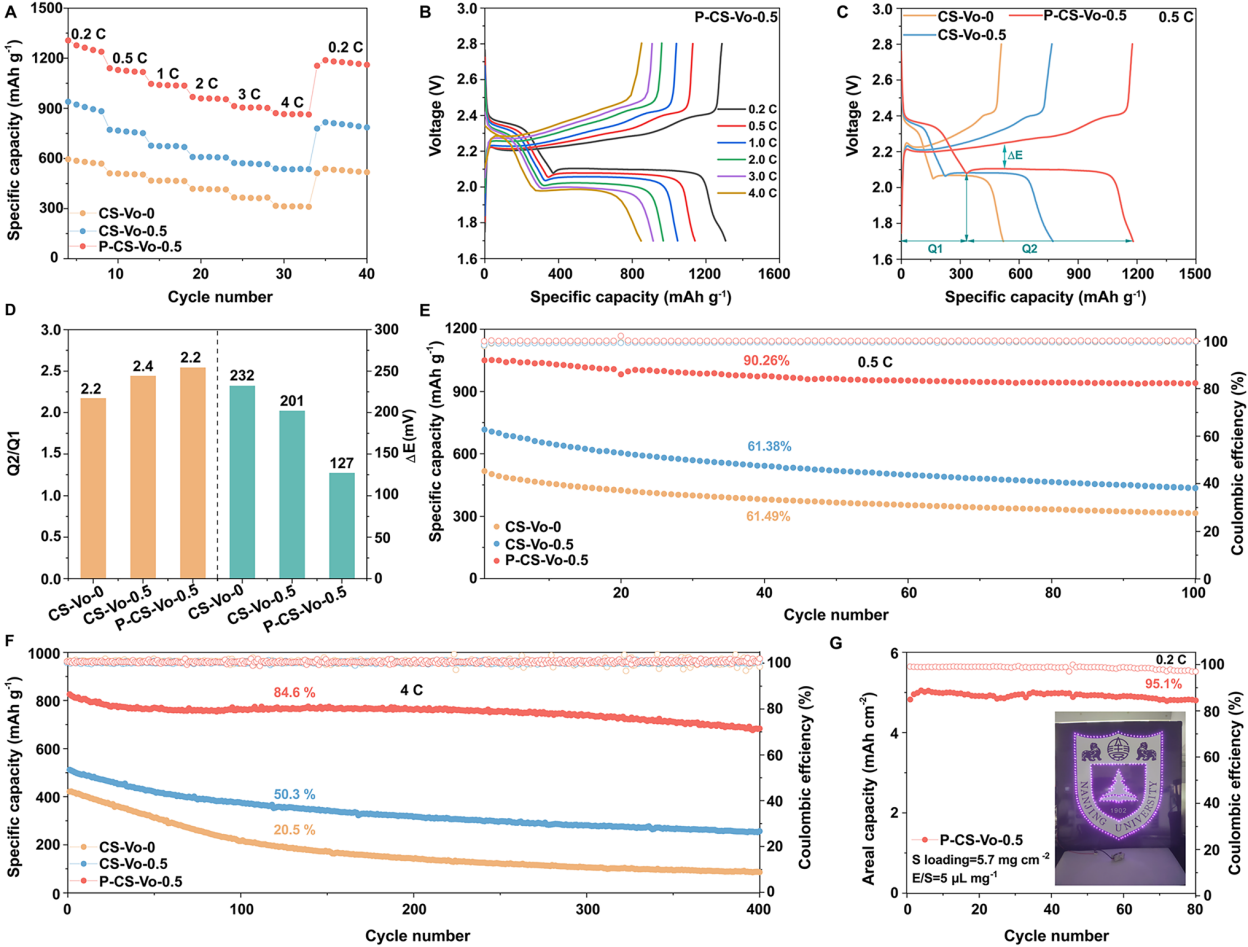
**A** Rate capabilities at different current rates **B** GCD curves of P-CS-Vo-0.5; **C** GCD curves for various electrodes measured at 0.5C and **D** the corresponding values of Q2/Q1 and ΔE; **E–F** Long-term cycling performance at 0.5C and 4C and **G** the capacity retention at high S loading and the inset shows a photograph of an LED powered by a Li–S cell with P-CS-Vo-0.5 separators

To comprehensively assess the impact of the catalyst on battery performance, Li–S batteries were assembled with different separators. As shown in Fig. 6A, the Li–S battery with the P-CS-Vo-0.5 separator exhibited exceptional rate performance, retaining a discharge capacity of 871.9 mAh g^−1^ at 4C (1C = 1675 mA g^−1^), significantly higher than CS-Vo-0.5 (540.4 mAh g^−1^) and CS-Vo-0 (316.8 mAh g^−1^). Figure 6B and S38 displays the voltage profiles at different current rates. Notably, the battery with the P-CS-Vo-0.5 separator maintained stable performance, whereas those with CS-Vo-0.5 and CS-Vo-0 separators suffered from severe polarization and capacity degradation. This high sulfur reaction efficiency was attributed to the strong catalytic activity of P-CS-Vo-0.5. These results demonstrated that the high sulfur reaction efficiency was caused by the strong catalytic activity of P-CS-Vo-0.5.

The potential difference (ΔE) between the charge and discharge plateaus was used to investigate the conversion and polarization dynamics of LiPSs [36]. The capacity contributions of two discharge platforms, denoted as Q1 and Q2, respectively, were utilized to analyze the catalytic activity for the conversion of polysulfide. The related ΔE and Q2/Q1 ratios are displayed in Fig. 6C, D. The battery with P-CS-Vo-0.5 separator exhibited the smallest polarization (ΔE = 127 mV) compared to CS-Vo-0.5 (ΔE = 201 mV) and CS-Vo-0 (ΔE = 232 mV), indicating reduced electrochemical polarization and rapid conversion kinetics. Moreover, the highest Q2/Q1 ratio of 2.5 for the battery with P-CS-Vo-0.5 separator suggested enhanced catalytic activity, facilitating a complete conversion of soluble Li_2_Sₙ to solid Li_2_S/Li_2_S_2_. Electrochemical reaction barrier measurements, as shown in Fig. S39, further confirm that the P-CS-Vo-0.5 separator lowers the nucleation and decomposition barriers of Li_2_S during discharge and charge, attributed to the synergistic effects of vacancies and P doping.

To further evaluate the electrochemical performance, the long-term cycling stability of the electrodes was tested. After 100 cycles at 0.5C, the battery with P-CS-Vo-0.5 separator retained 90.26% of its initial capacity, with an initial specific capacity of 1041.1 mAh g^−1^, Compared with CS-Vo-0.5 (716.1 mAh g^−1^, 61.38%) and CS-Vo-0 (516.0 mAh g^−1^, 61.49%), as illustrated in Fig. 6E. The disassembled batteries showed pronounced anode corrosion and severe cracking on the lithium foils and separators of CS-Vo-0.5 and CS-Vo-0, whereas the P-CS-Vo-0.5 separator and lithium foil exhibited smooth surfaces, indicating minimal anode corrosion and effective prevention of LiPSs interactions (Fig. S40). At a high rate of 4C, the battery with the P-CS-Vo-0.5 separator demonstrated capacity retention of 84.6% over 400 cycles, significantly outperforming the CS-Vo-0.5 and CS-Vo-0 separators, which exhibited rapid capacity fading with retention rates of 50.3% and 20.5%, respectively (Fig. 6F). Under high-rate cycling at 4C, the battery exhibits a non-monotonic capacity evolution—initially decreasing, then recovering, and eventually declining—reflecting the staged nature of electrochemical processes. The initial capacity drop arises from polysulfide dissolution and unstable SEI formation. Subsequent recovery is attributed to gradual electrolyte infiltration and reactivation of electrochemically active species, while long-term degradation is driven by structural deterioration of the electrodes. This stark difference highlighted the critical role of the P-CS-Vo-0.5 catalyst in enhancing both catalytic and adsorption capabilities. In addition, it is observed that the cycling performance of batteries using P-CS-Vo-0.5 separator is superior to other recently reported batteries (Fig. S41) [40, 41]. The development of high-energy–density Li–S batteries and their competitiveness against current lithium-ion batteries hinges on optimizing high-sulfur loading performance [42]. A comprehensive electrochemical assessment was conducted on a battery with a high sulfur loading of 5.7 mg cm^−2^ (Fig. 6G). The battery with the P-CS-Vo-0.5 separator delivered an areal capacity of 5.04 mAh cm^−2^ and retained 95.1% of its capacity after 80 cycles. Notably, the successful illumination of patterned LEDs at Nanjing University using the P-CS-Vo-0.5-based battery further demonstrates its practical applicability, marking a significant step toward the commercial viability of Li–S batteries.

## Conclusions

In summary, we developed a novel “heteroatoms synergistic anchoring vacancies” strategy to synthesize P-CS-Vo-0.5 catalyst with ultrahigh activity and stability. DFT calculations and experiments establish a volcano relationship between catalytic activity and vacancy concentration, with CS-Vo-0.5 exhibiting optimal performance. *P* incorporation enabled vacancy pinning, mitigating vacancy migration and overcoming the activity-stability trade-off. Experimental results showed that the battery with P-CS-Vo-0.5 separator achieved a high sulfur utilization rate of 1306.7 mA h g^−1^ at 0.2C, while also exhibiting excellent long-term cycling stability. After 400 cycles at 4C, the capacity retention rate remained at 84.6%. This work utilizes a heteroatoms synergistic anchoring vacancies strategy, offering a transformative approach for designing vacancy-engineered catalysts with exceptional activity and stability.

## Supplementary Information

Below is the link to the electronic supplementary material.

Supplementary file1 (DOCX 21865 KB)
