# Supplementary material for: Heteroatoms Synergistic Anchoring Vacancies in Phosphorus-Doped CoSe2 Enable Ultrahigh Activity and Stability in Li–S Batteries
Source: Nanomicro Lett. 2025 Jun 23;17:308. doi: 10.1007/s40820-025-01806-0 (PMC12185838; doi:10.1007/s40820-025-01806-0)
Supplement: Supplementary file 1 — Supplementary file1 (DOCX 21865 KB) [file 40820_2025_1806_MOESM1_ESM.docx]

Supporting Information for

**Heteroatoms Synergistic Anchoring Vacancies in Phosphorus-Doped CoSe_2_ Enable Ultrahigh Activity and Stability in Li-S Batteries**

Xiaoya Zhou^1†^, Wei Mao^1†^, Chengwei Ye^1^, Qi Liang^1^, Peng Wang^1^, Xuebin Wang^1*^, Shaochun Tang^1*^

^1^ Key National Laboratory of Solid State Microstructures, Collaborative Innovation Center of Advanced Microstructures, Jiangsu Key Laboratory of Artificial Functional Materials, College of Engineering and Applied Sciences, Nanjing University, Nanjing 210093, P. R. China

† Xiaoya Zhou and Wei Mao contributed equally to this work.

*Corresponding authors. E-mail: [wangxb@nju.edu.cn](mailto:wangxb@nju.edu.cn) (Xuebin Wang); [tangsc@nju.edu.cn](mailto:tangsc@nju.edu.cn) (Shaochun Tang)

**S1 Experimental Section**

**Materials Characterizations:**

The crystal structure of the composites was tested by X-ray diffraction with Cu Kα radiation (XRD, D8 Advance). The surface elemental composition was analyzed by X-ray photoelectron spectroscopy (XPS, Thermo Scientific). The structure and morphology of samples were observed by transmission electron microscope (TEM, Tecnai F20). The spherical aberration-corrected HAADF-STEM images were collected was collected in the spherical-aberration corrected FEI titan cubed G2 60-300 operating at 300 kV. Thermogravimetric analysis (TGA) was obtained on a TG/DTA7300 analyzer at a heating rate of 10 °C min^-1^ under a N_2_ atmosphere. The UV-Vis spectra was measured using a SHIMADZU UV-3600 spectrophotometer. The specific surface area and pore size distribution of separators were analyzed by Brunauer-Emmett-Teller using a surface area analyzer (JW-BK100C).

**Visualized Adsorption of Lithium Polysulfides:**

Firstly, S and Li_2_S (molar ratio 5:1) were mixed and dissolved into DME to obtain 0.05 mol L^-1^ Li_2_S_6_, which was stirred at 60℃ for 48 h. The target materials (15 mg) were added to the prepared solution (1.5 mL) for 12 h, the UV-Vis absorption spectra were tested.

**Li_2_S** **nucleation and dissolution:**

S and Li_2_S with a molar ratio of 7:1 were mixed into Li-S electrolyte (0.5 M LiTFSI and 0.5 M LiNO_3_ additive, DOL: DME = 1:1, v:v) and stirred at 60 ℃ for 24 h to obtain 0.2 M Li_2_S_8_ solution. 20 μL Li_2_S_8_ was injected into the cathode and 20 μL Li-S electrolyte was dropped into the lithium anode. A constant current of 0.112 mA was used to discharge to 2.06 V, and then discharge at constant voltage (2.02 V) to below 10^-5^ A. For the dissolution tests, the assembled cells were first galvanostatically (0.1 mA) discharged to 1.70 V. Then, the battery was charged at 2.35 V until 30000 s for the sufficient dissolution of Li_2_S.

**Preparation of Separator:**

70% active material of the cathode, 20% carbon black, and 10% polyvinylidene fluoride (PVDF) were mixed in N-methyl-2-pyrrolidone (NMP), and the mixture was pasted onto celgard 2500 membrane and dried at 50 °C for 8 h to obtain the modified separator.

**Electrochemical Tests:**

80% S/CNT, Super P, and PVDF (7:2:1 wt%) were completely mixed into a slurry, coated onto a piece of carbon-coated Al foil, and then vacuum dried under 50 ^o^C for 6 h. The obtained cathode was cut into disks (diameter of 12 mm) with the S mass loading of 1~2 mg cm^-2^. The areal loading for the interlayer was approximately 0.3 mg cm^-2^. The electrolyte/sulfur (E/S) ratio was·maintained at 30~40 µL mg^-1^ for each cell. The battery was assembled with lithium metal as cathode, 80% S/CNT as the anode, modified separator, 0.5 M LiTFSI in DME/DOL (1:1, vol%) solvent and 0.5 M LiNO_3_ as electrolyte. The galvanostatic charge/discharge test (1.7-2.8 V) was conducted on the LAND CT3001A battery testing system instrument. CV was tested at 0.1~0.5 mV s^-1^ and a scan range from 1.7 to 2.8 V vs. Li/Li^+^ and EIS (100 kHz to 0.01 Hz) was obtained on a CHI760E electrochemical workstation (Chenhua, Shanghai, China).

The ionic conductivity can be determined using the following equation:

$\delta=\frac{d}{R\cdot A}$ (S-1)

where *d* (cm) and *A* (cm^2^) are the thickness and area of the separator, and *R* (Ω) is the bulk resistance.

The calculation of the Li-ion transference number ($t_{{Li}^{+}}$)is specifically carried out using Bruce and Vincent's correction formula.

$$t_{{Li}^{+}}\text{=}\frac{I_{s}(\Delta V-I_{0}R_{0})}{I_{0}(\Delta V-I_{S}R_{s})} \text{(S-2)}$$

where $I_{0}$ represents the initial current value; $I_{S}$ stands for the constant current value; ΔV is the applied constant potential difference value (10 mV); $R_{0}$ is the interface impedance before polarization, and $R_{S}$ is the interface impedance after polarization.

**Methods**

We have employed the first-principles [S1, S2] to perform density functional theory (DFT) calculations within the generalized gradient approximation (GGA) using the Perdew-Burke-Ernzerhof (PBE) formulation [S3]. We have chosen the projected augmented wave (PAW) potentials [S4-S6] to describe the ionic cores and take valence electrons into account using a plane wave basis set with a kinetic energy cutoff of 520 eV. The GGA+U method was adopted in our calculations. The value of the effective Hubbard U was set as 4.615 eV for Co Partial occupancies of the Kohn-Sham orbitals were allowed using the Gaussian smearing method and a width of 0.05 eV. The electronic energy was considered self-consistent when the energy change was smaller than 10^−5^ eV. A geometry optimization was considered convergent when the energy change was smaller than 0.05 eV Å^-1^. The Brillouin zone integration is performed using 2×2×1 Monkhorst-Pack k-point sampling for a structure. Finally, the adsorption energies (E_ads_) were calculated as E_ads_=E_ad/sub_ -E_ad_ -E_sub_, where E_ad/sub_, E_ad_, and E_sub_ are the total energies of the optimized adsorbate/substrate system, the adsorbate in the structure, and the clean substrate, respectively. The free energy was calculated using the equation:

G=E_ads_+ZPE-TS

where G, Eads, ZPE and TS are the free energy, total energy from DFT calculations, zero point energy and entropic contributions, respectively, where T is set to 300K.

**S2 Supplementary Figures and Tables**


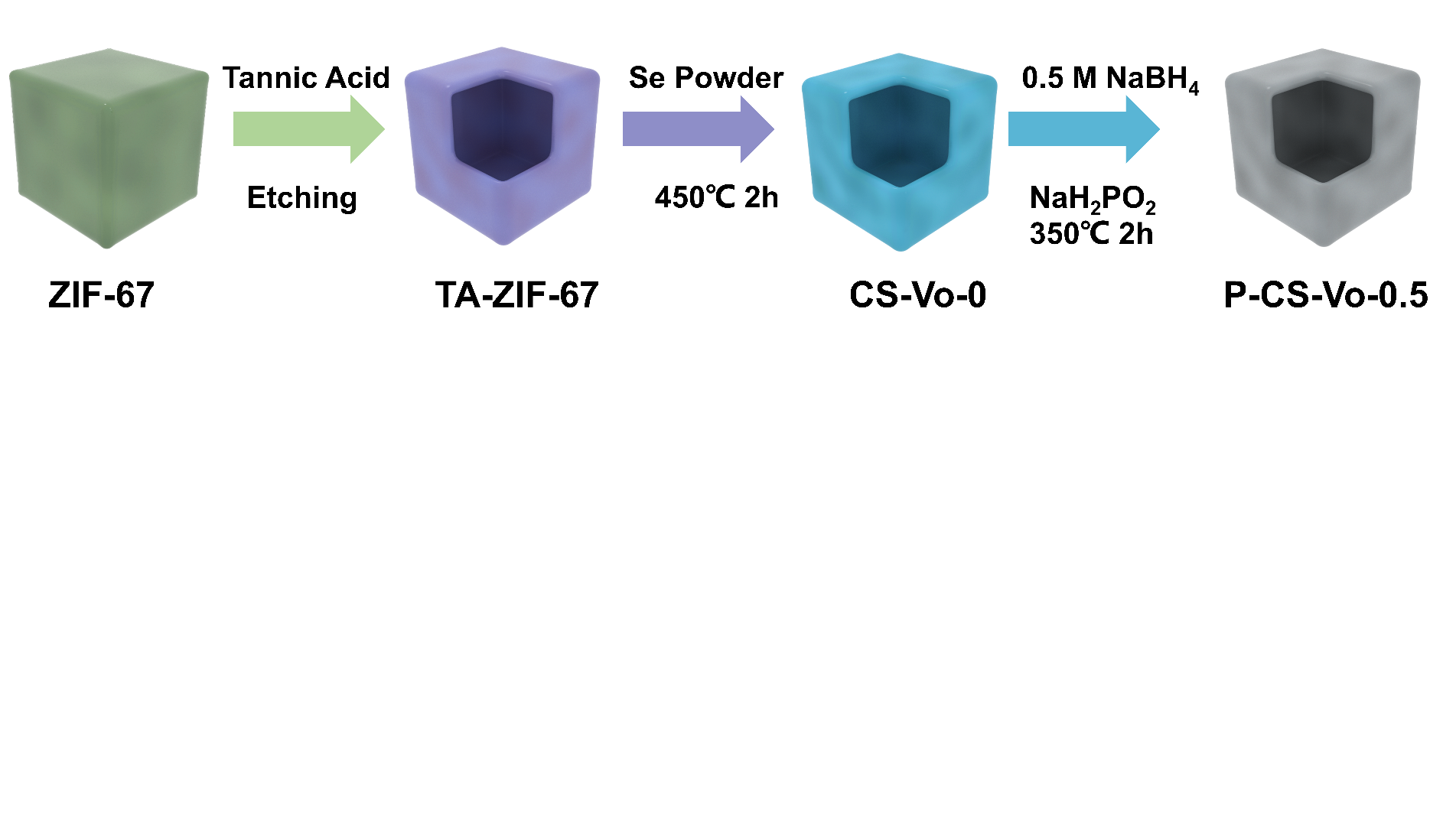


**Fig. S1** Schematic illustration of the P-CS-Vo-0.5 fabrication process


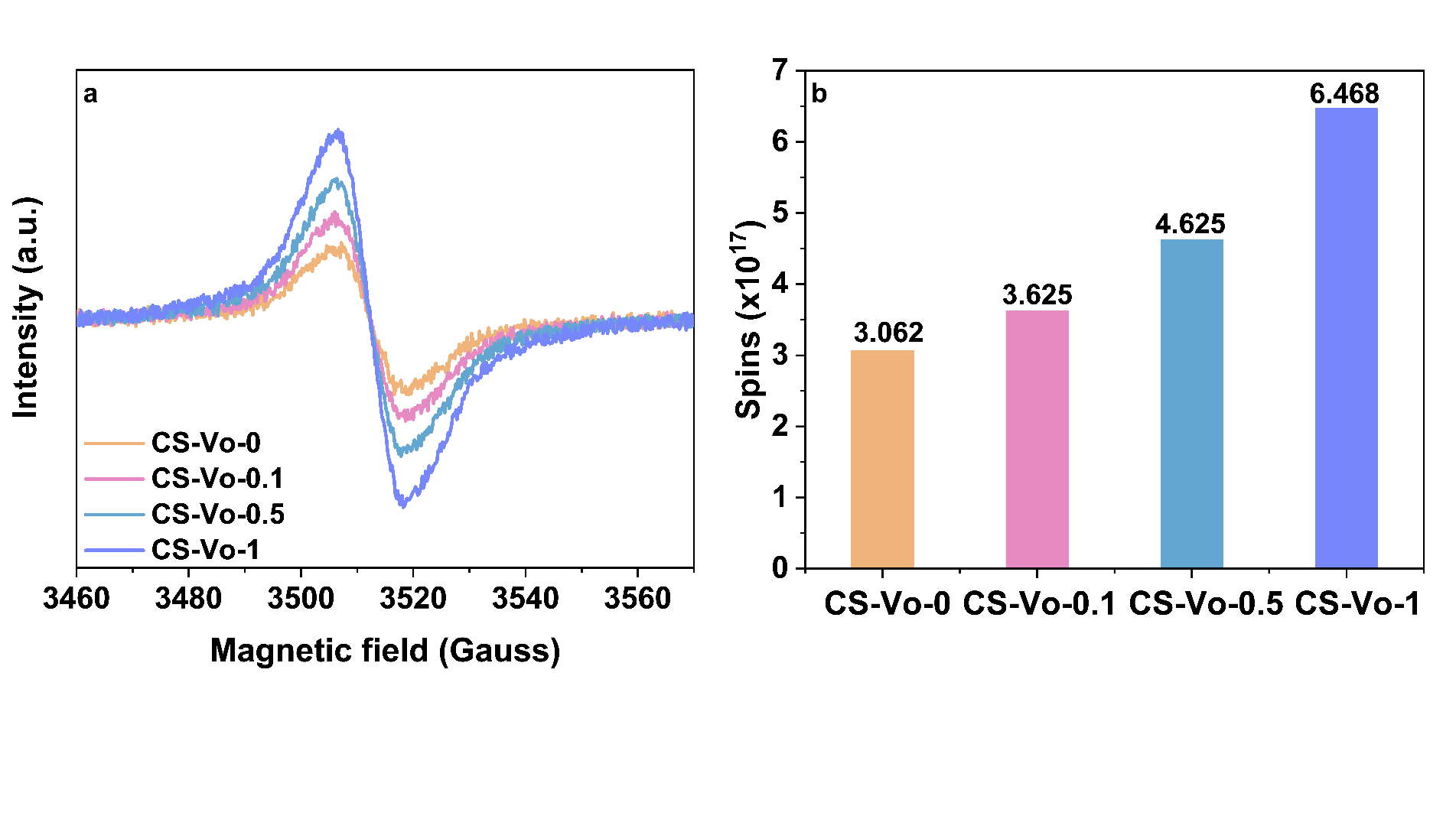


**Fig. S2** (**a**) EPR spectra, and (**b**) the corresponding vacancy concentration of different samples


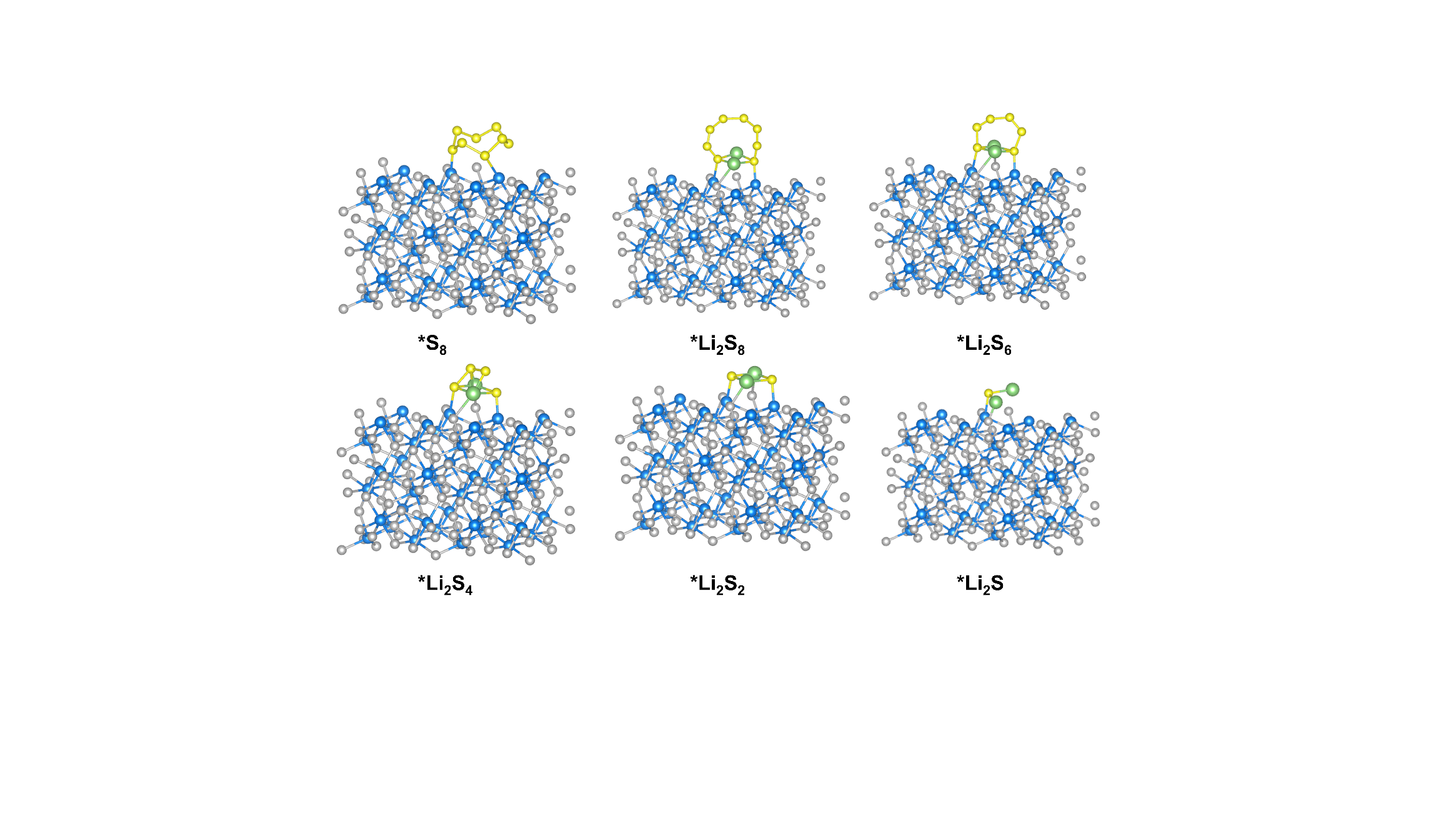


**Fig. S3** Optimized configurations of sulfur species absorption on CS-Vo-0


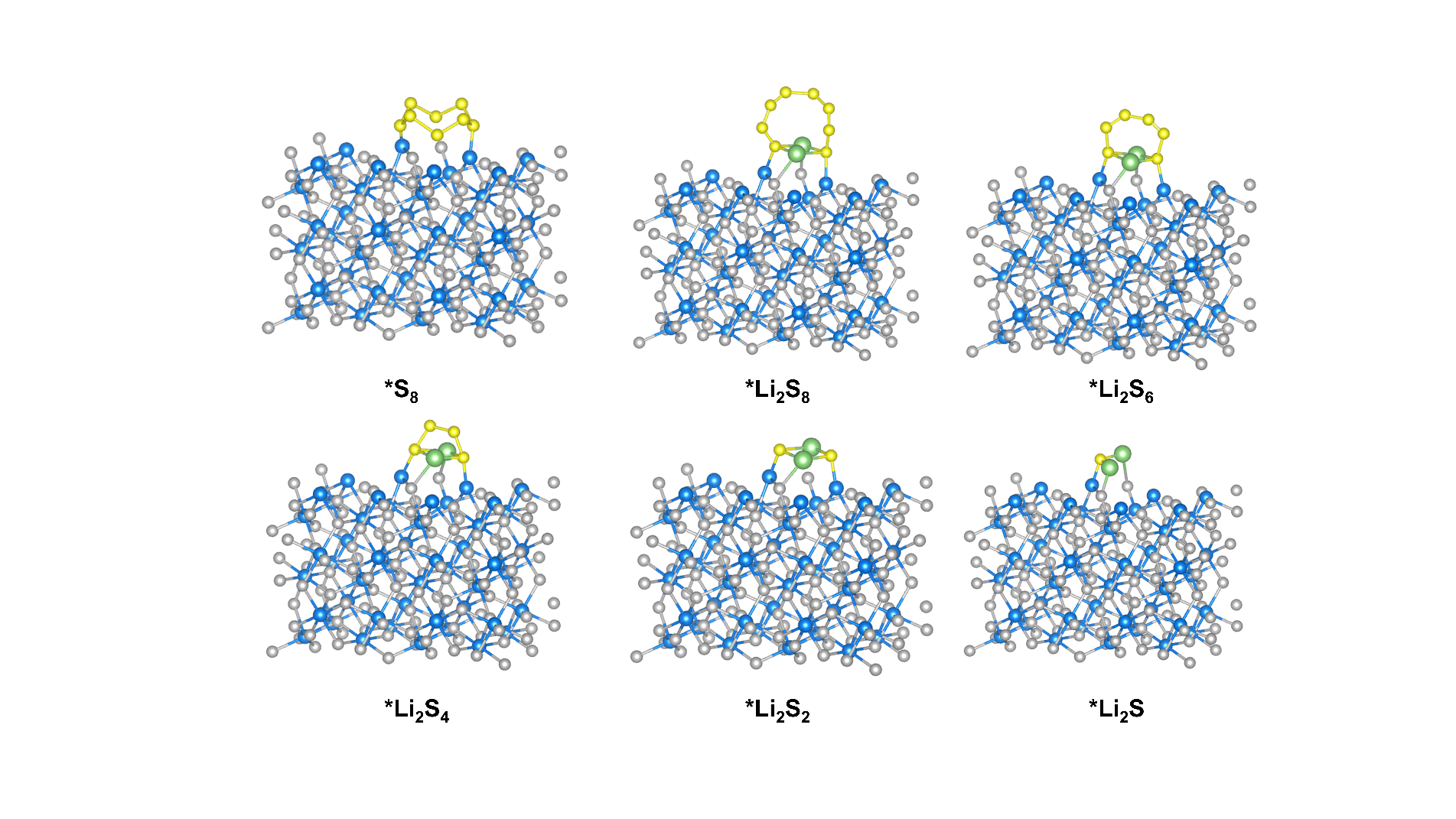


**Fig. S4** Optimized configurations of sulfur species absorption on CS-Vo-0.1


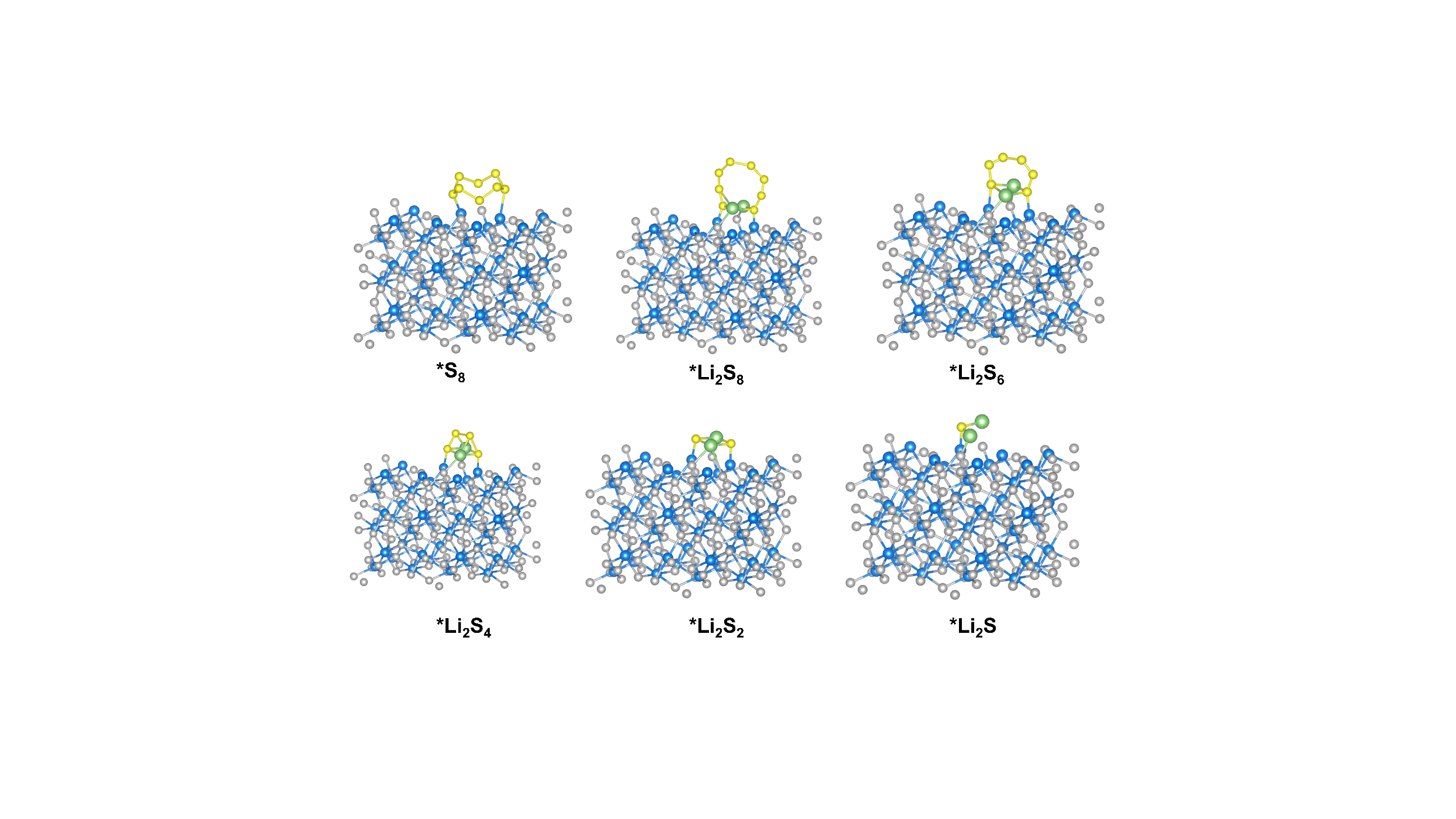


**Fig. S5** Optimized configurations of sulfur species absorption on CS-Vo-0.5


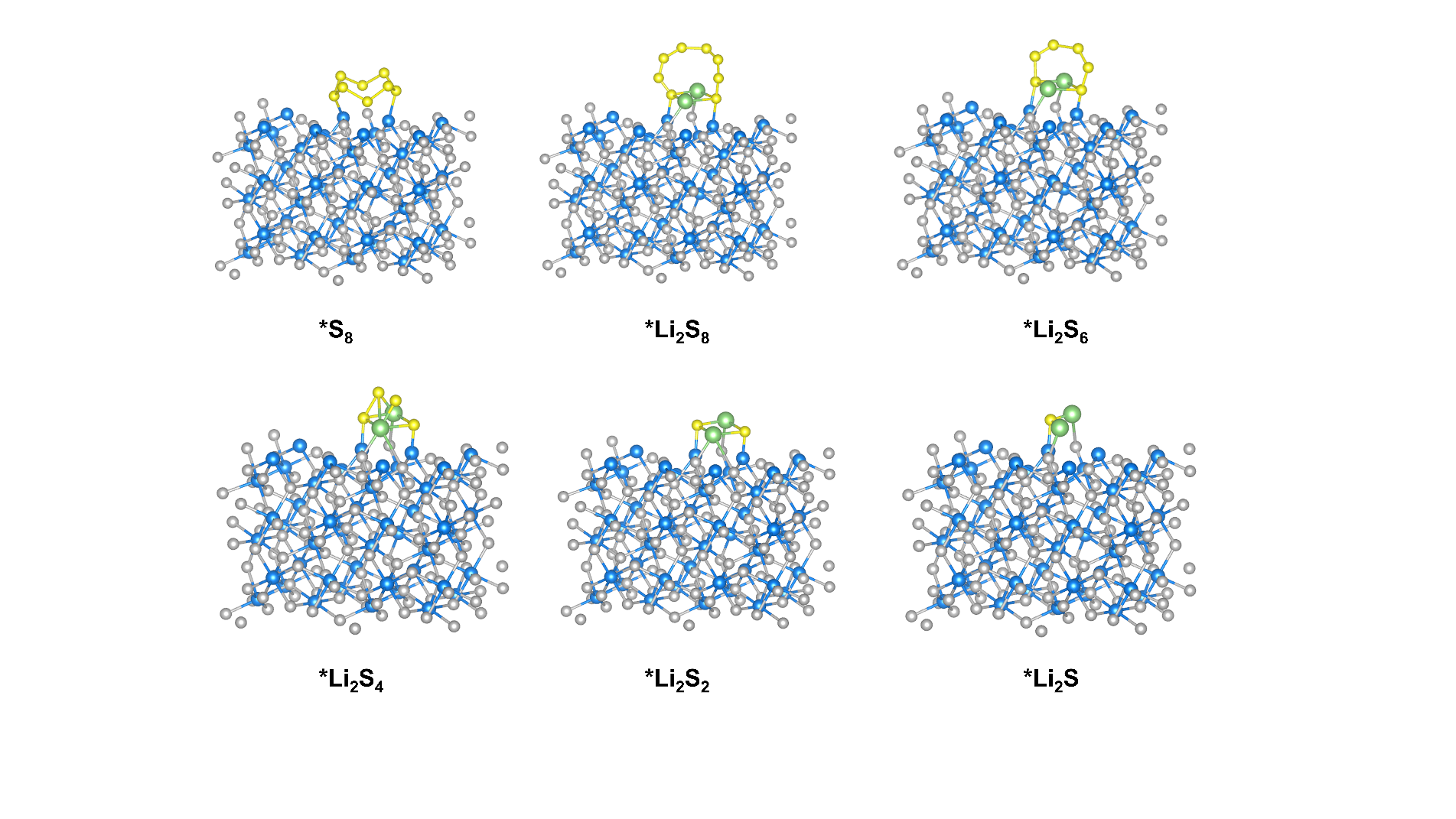


**Fig. S6** Optimized configurations of sulfur species absorption on CS-Vo-1


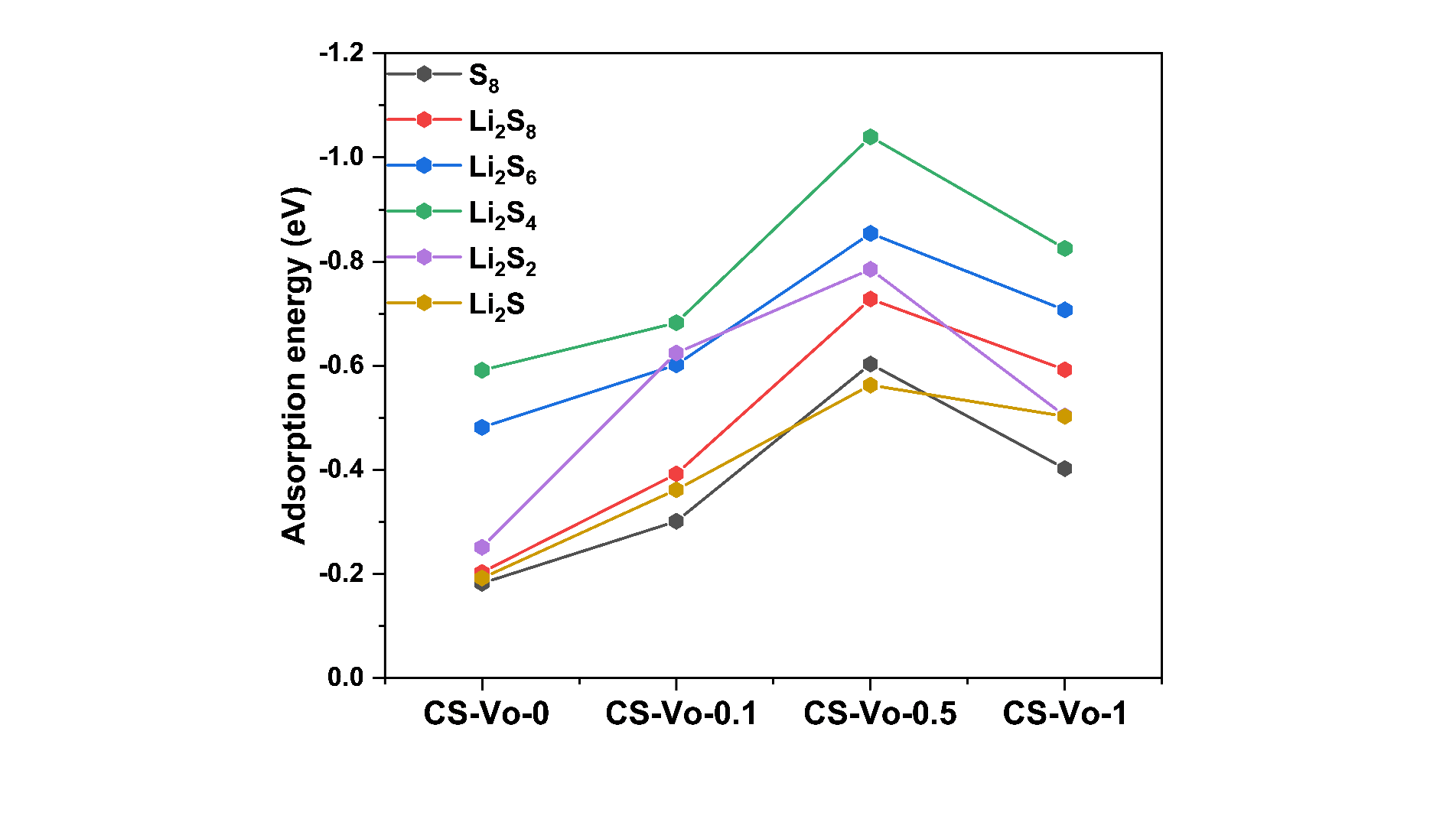


**Fig. S7** the calculated adsorption energy of sulfur species


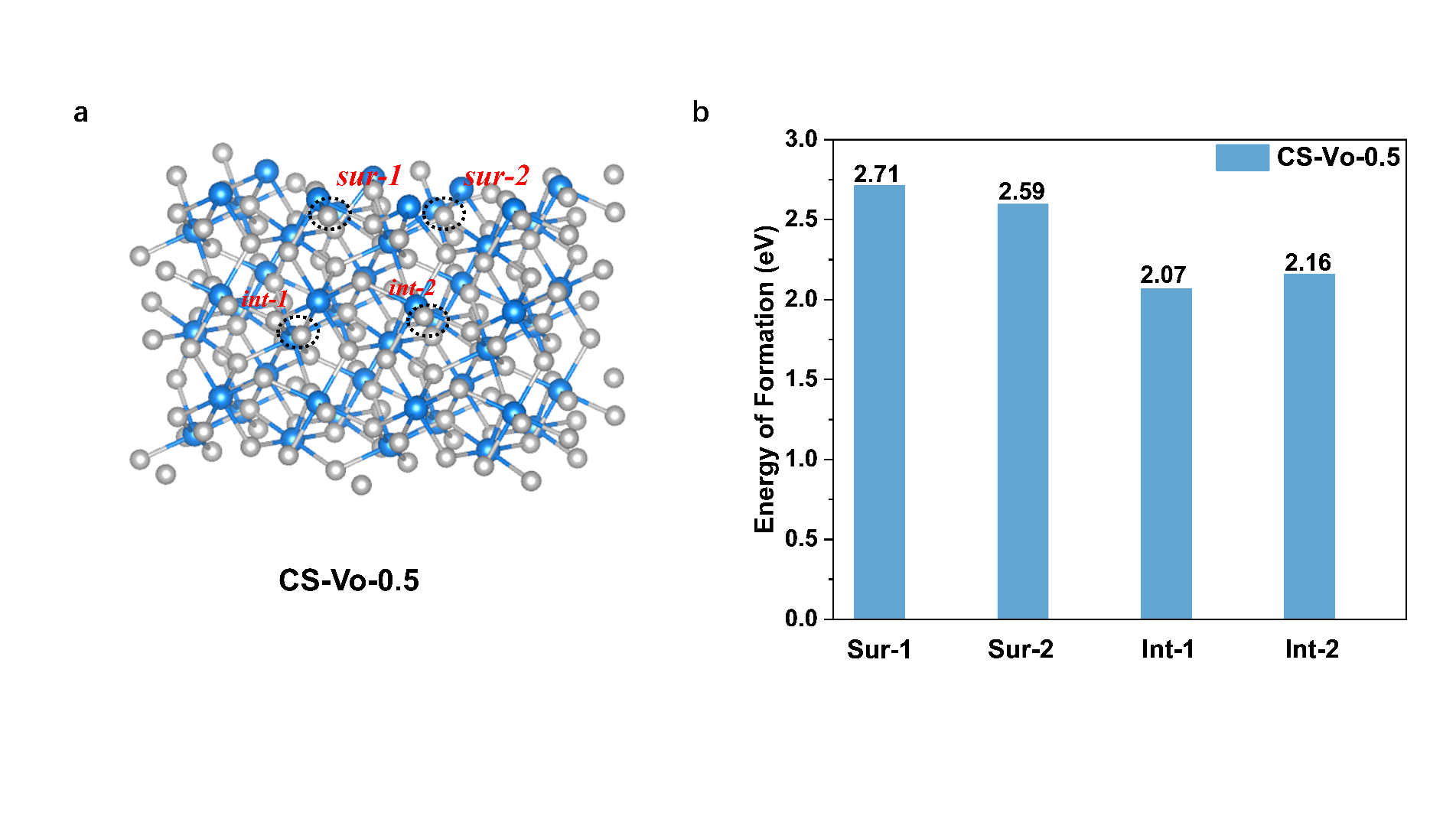


**Fig. S8** Schematic diagram of vacancies in different positions of (a) CS-Vo-0.5 and the formation energies of vacancies in different positions


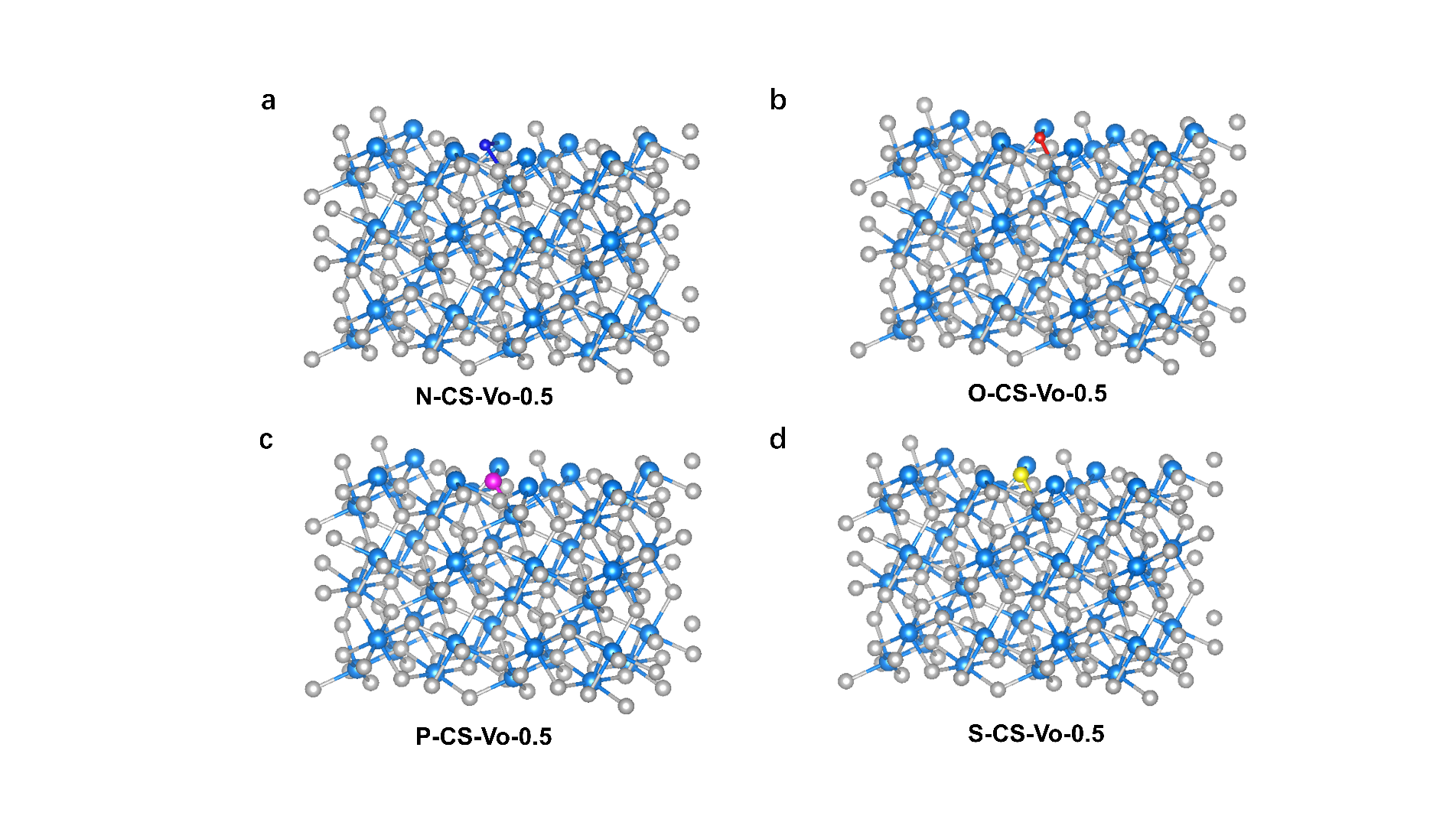


**Fig. S9** Crystal structure of (**a**) N-CS-Vo-0.5, (**b**) S-CS-Vo-0.5, (**c**) O-CS-Vo-0.5 and (**d**) P-CS-Vo-0.5


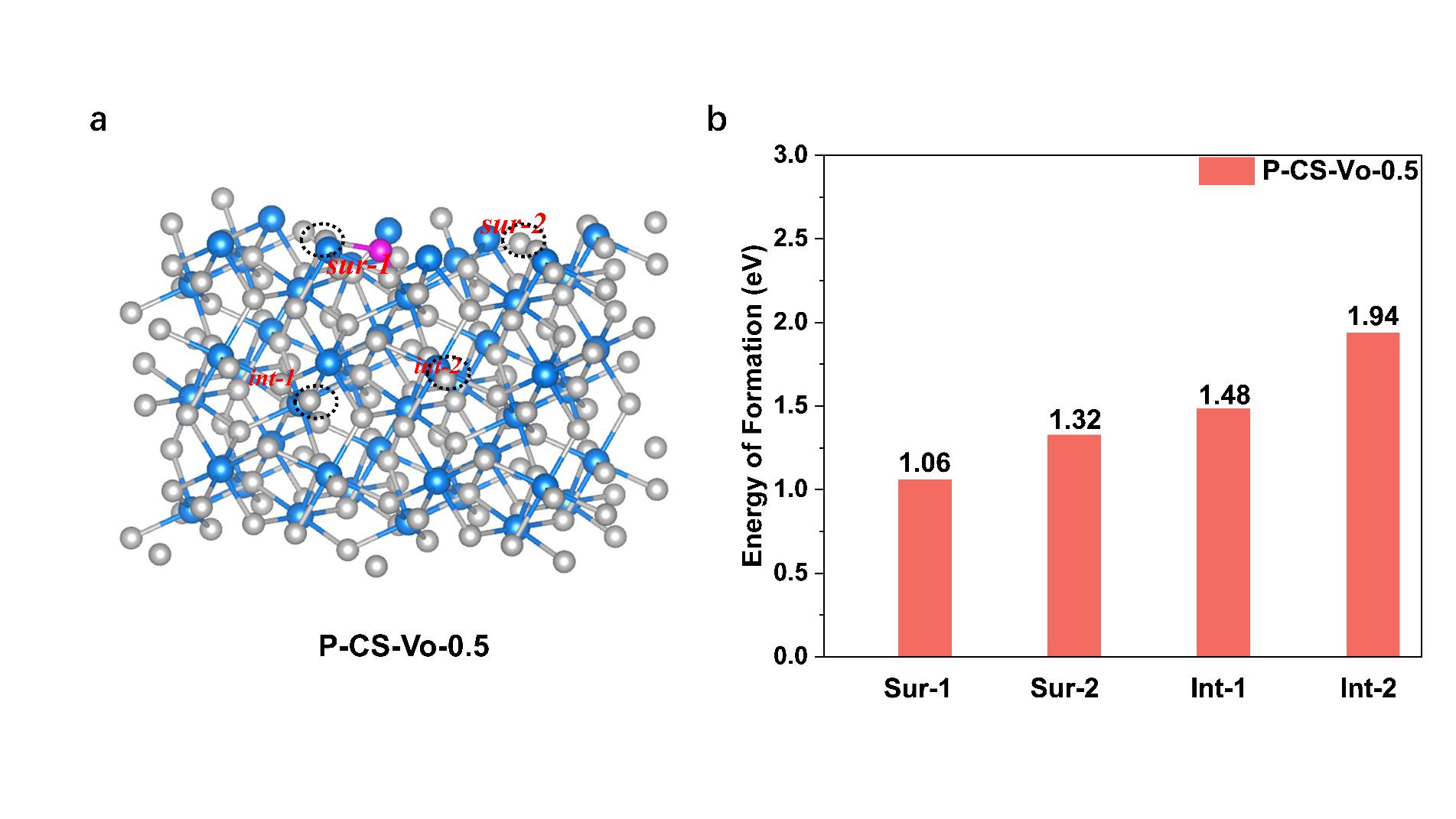


**Fig. S10** Schematic diagram of vacancies in different positions of (**a**) P-CS-Vo-0.5 and (**b**) the formation energies of vacancies in different positions


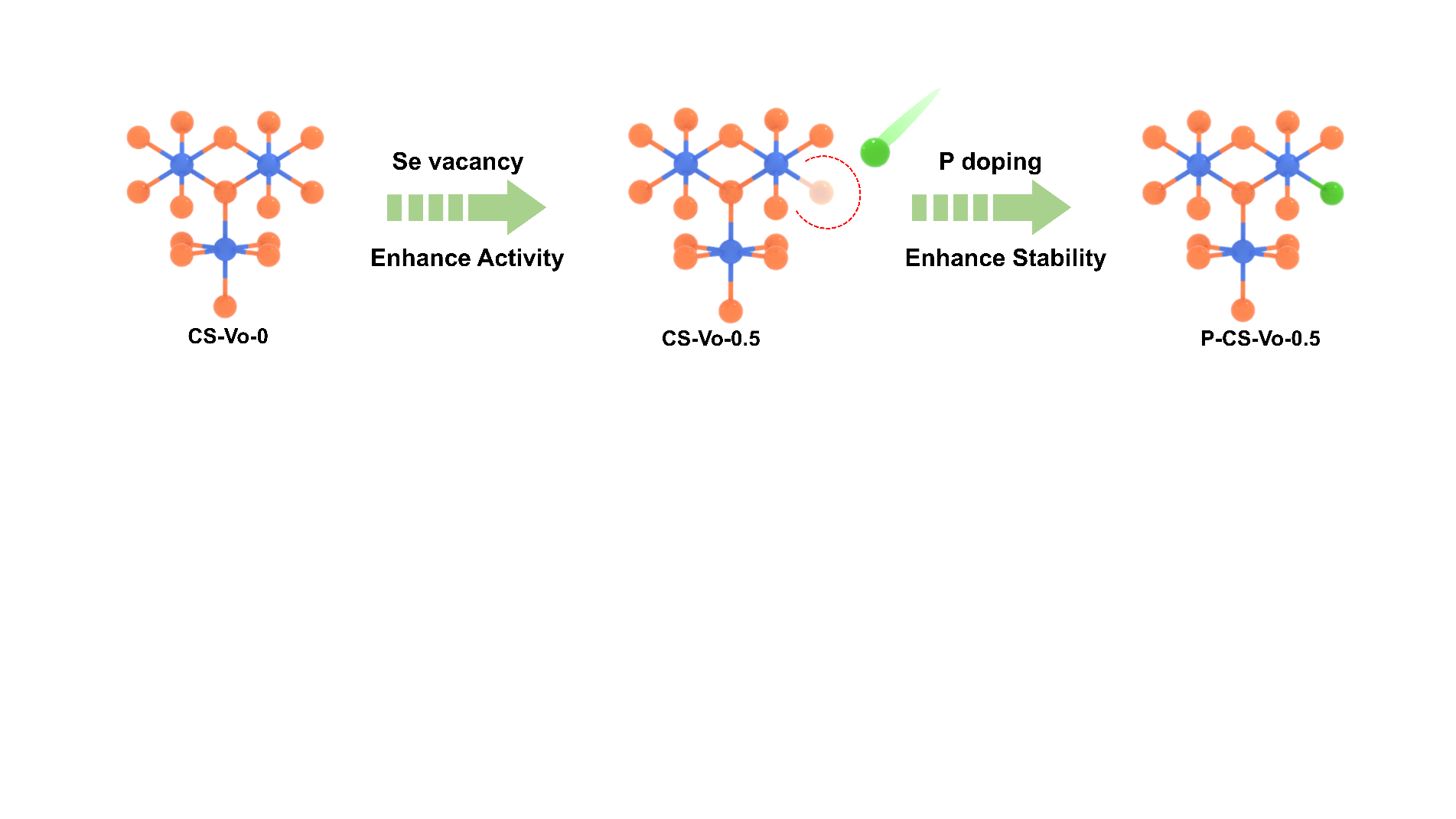


**Fig. S11** Schematic illustration of the synthesis of P-CS-Vo-0.5


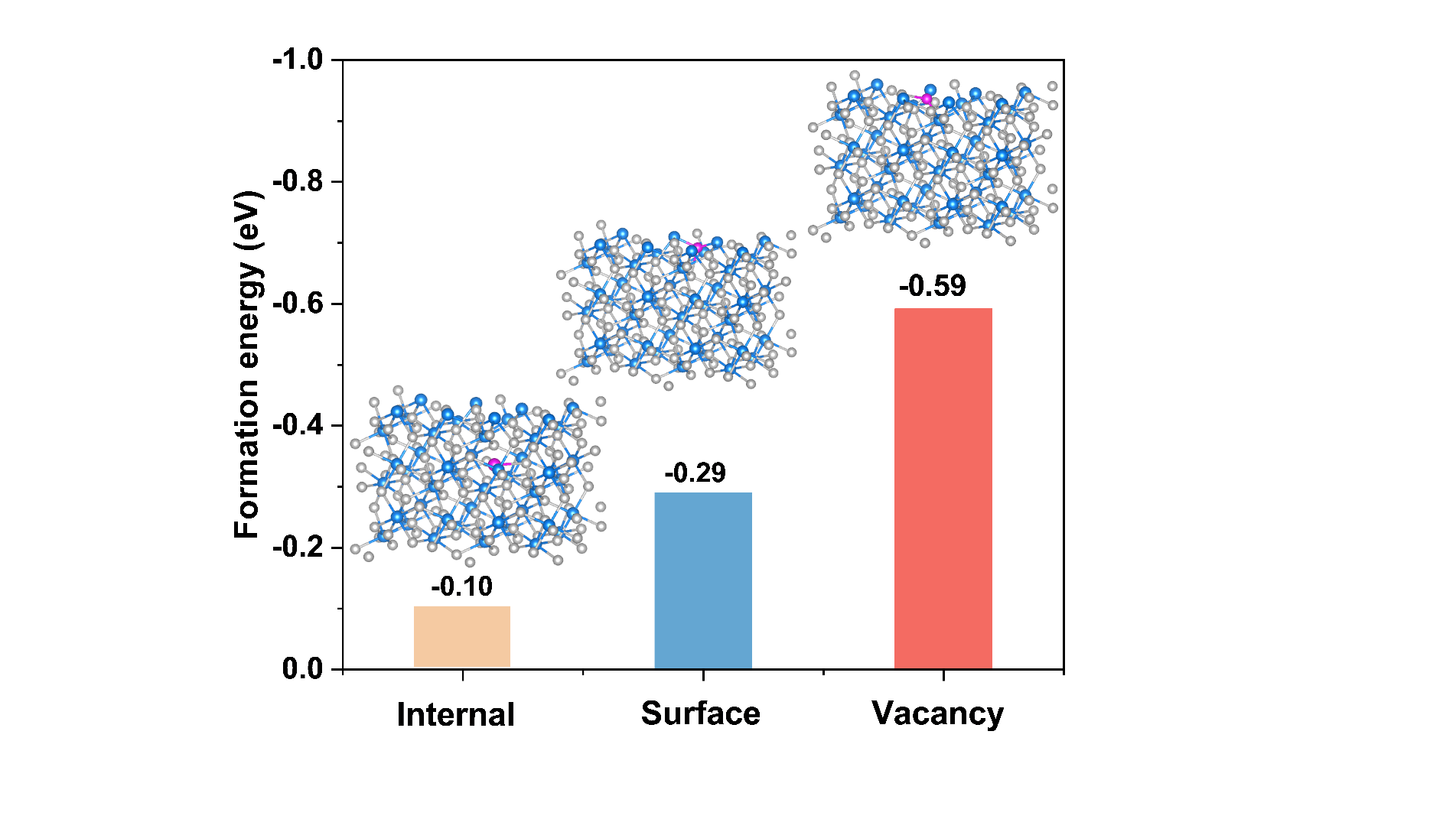


**Fig. S12** The formation energy of the P substituted position


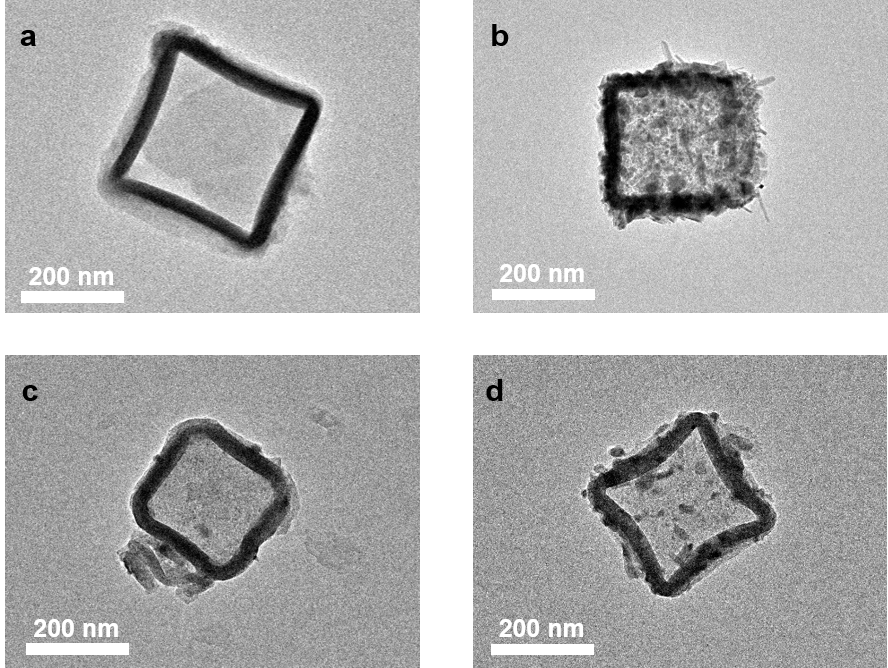


**Fig. S13** TEM image of (a) TA-ZIF-67, (b) CS-Vo-0, (c) CS-Vo-0.5 and (d) P-CS-Vo-0.5


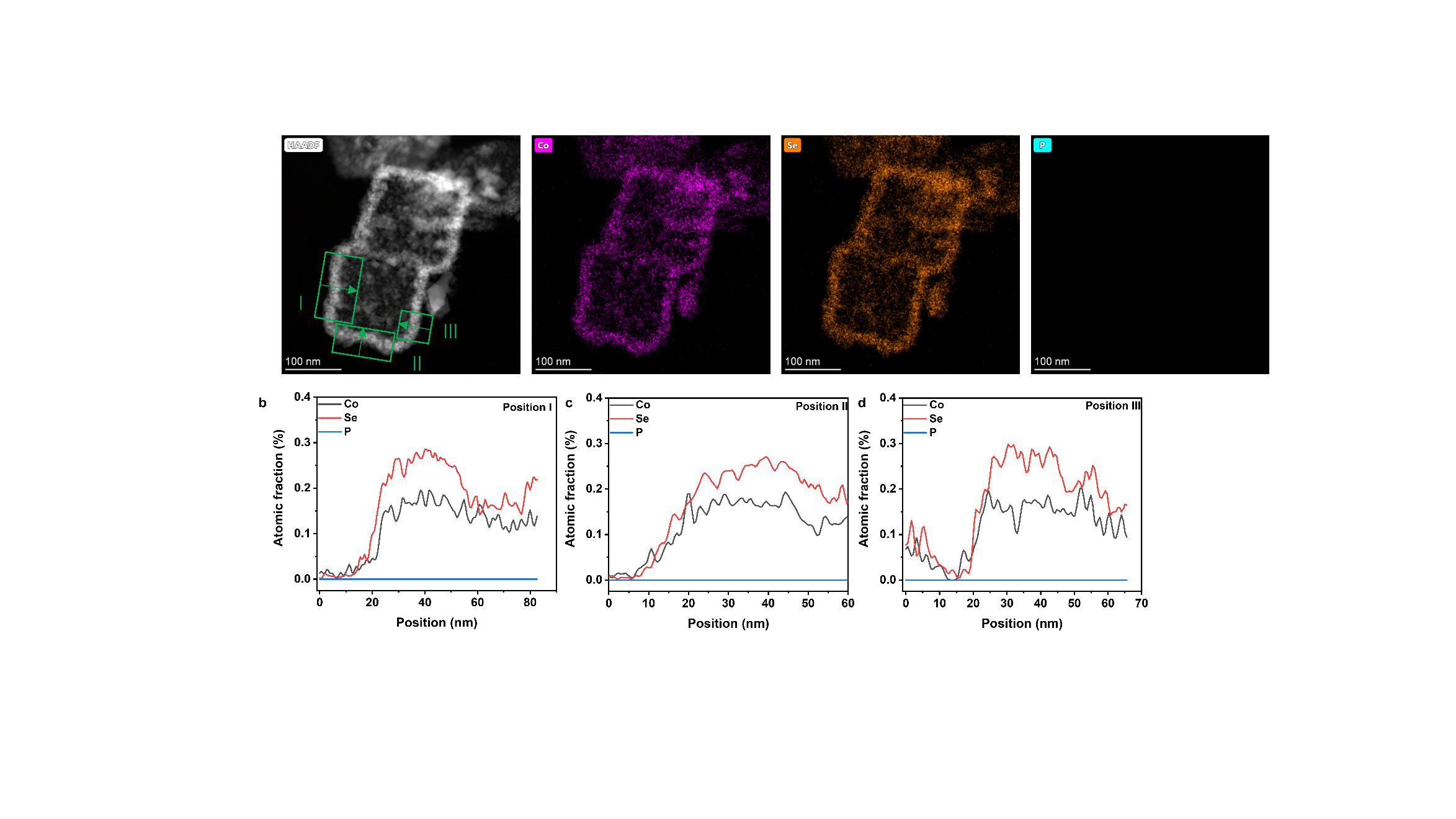


**Fig. S14** (**a**) HAADF image and corresponding EDS image and (**b**) the proportion of elements in different locations of CS-Vo-0


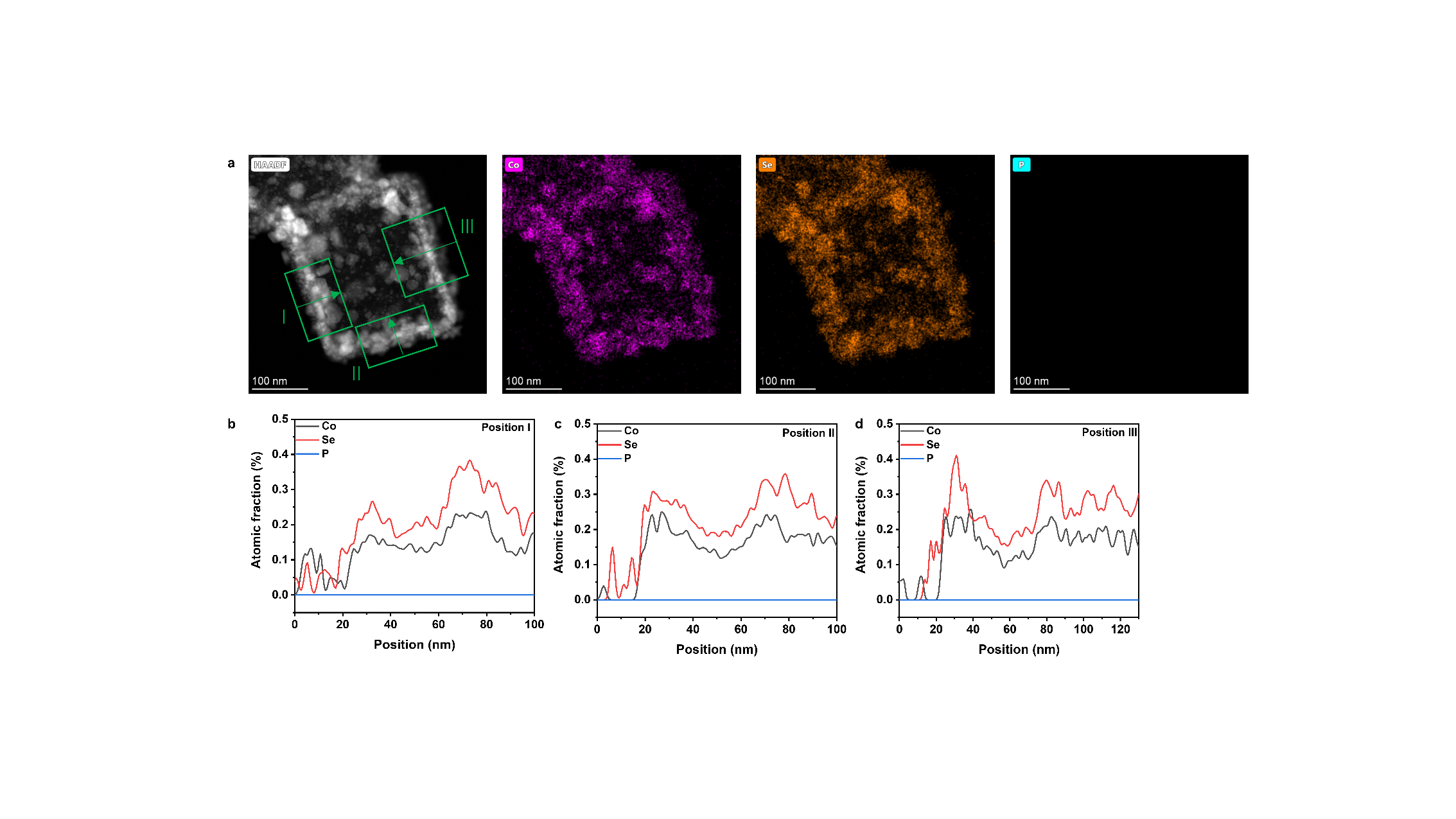


**Fig. S15** (**a**) HAADF image and corresponding EDS image and (**b**) the proportion of elements in different locations of CS-Vo-0.5


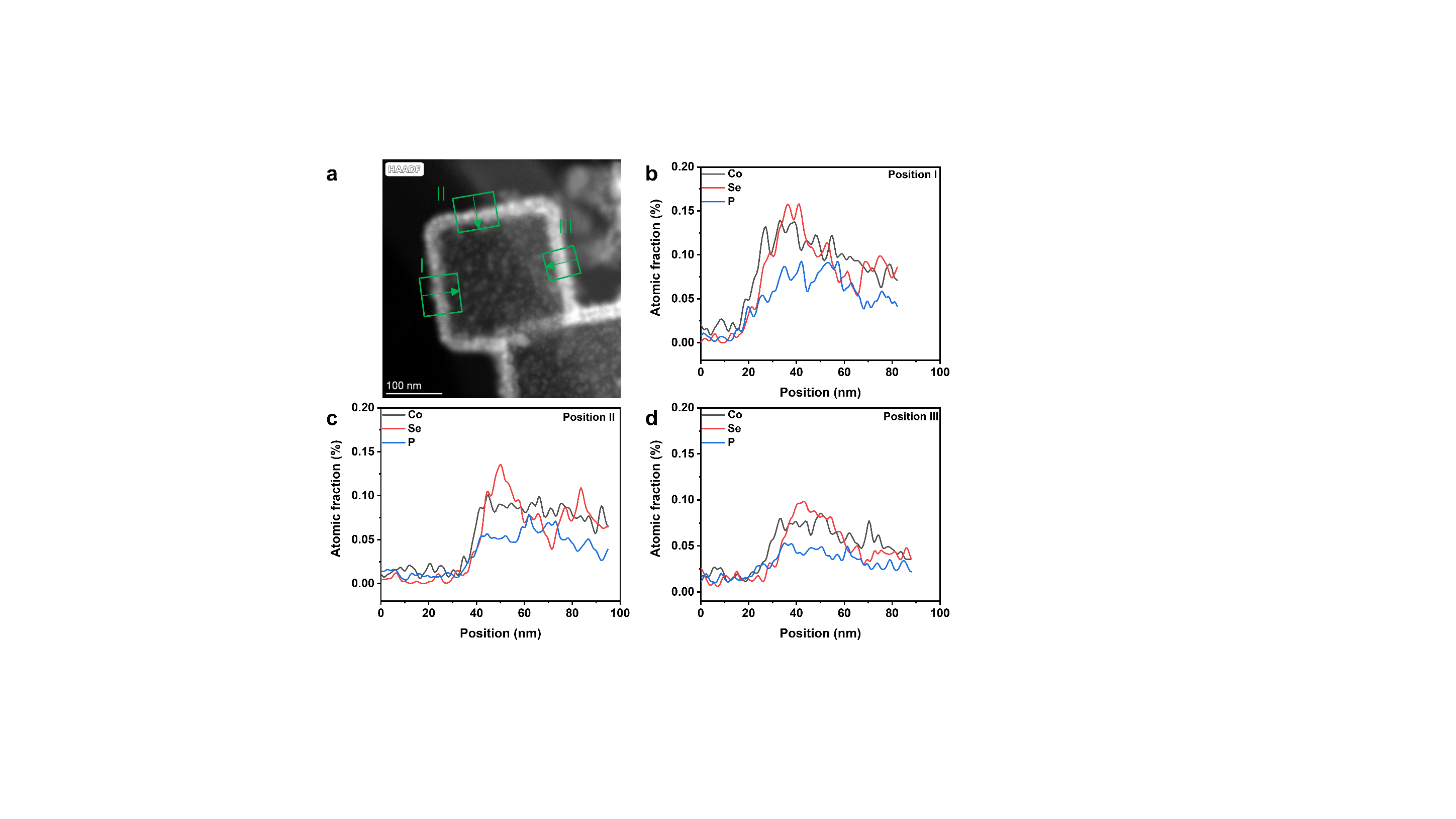


**Fig. S16** (**a**) HAADF image and (**b**) the proportion of elements in different locations of P-CS-Vo-0.5


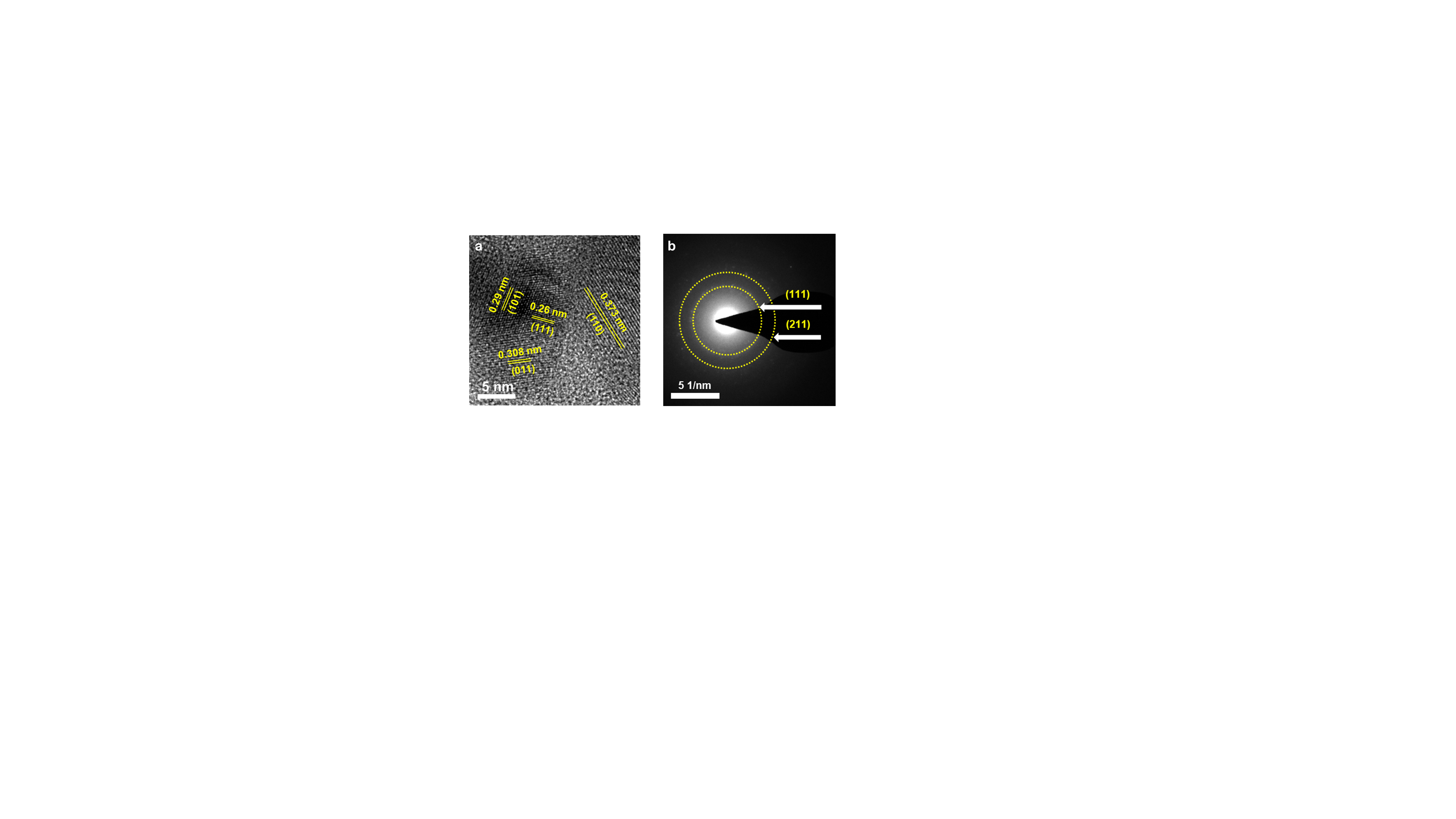


**Fig. S17** (**a**) HRTEM image and (**b**) SAED image of P-CS-Vo-0.5


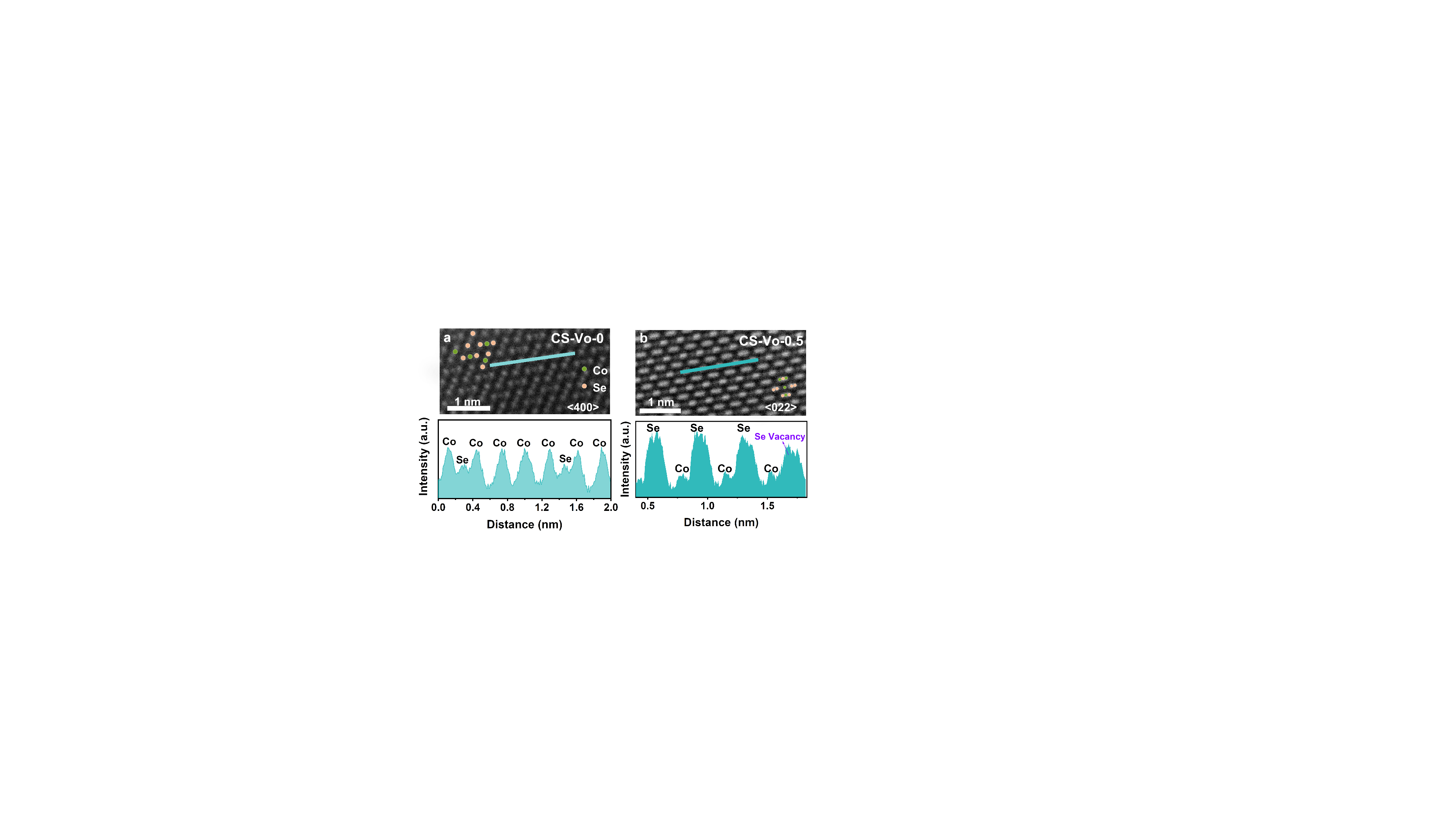


**Fig. S18** AC-HADDF-STEM images and line-scanning intensity profile acquired from the blue line of (**a**) CS-Vo-0 and (**b**) CS-Vo-0.5


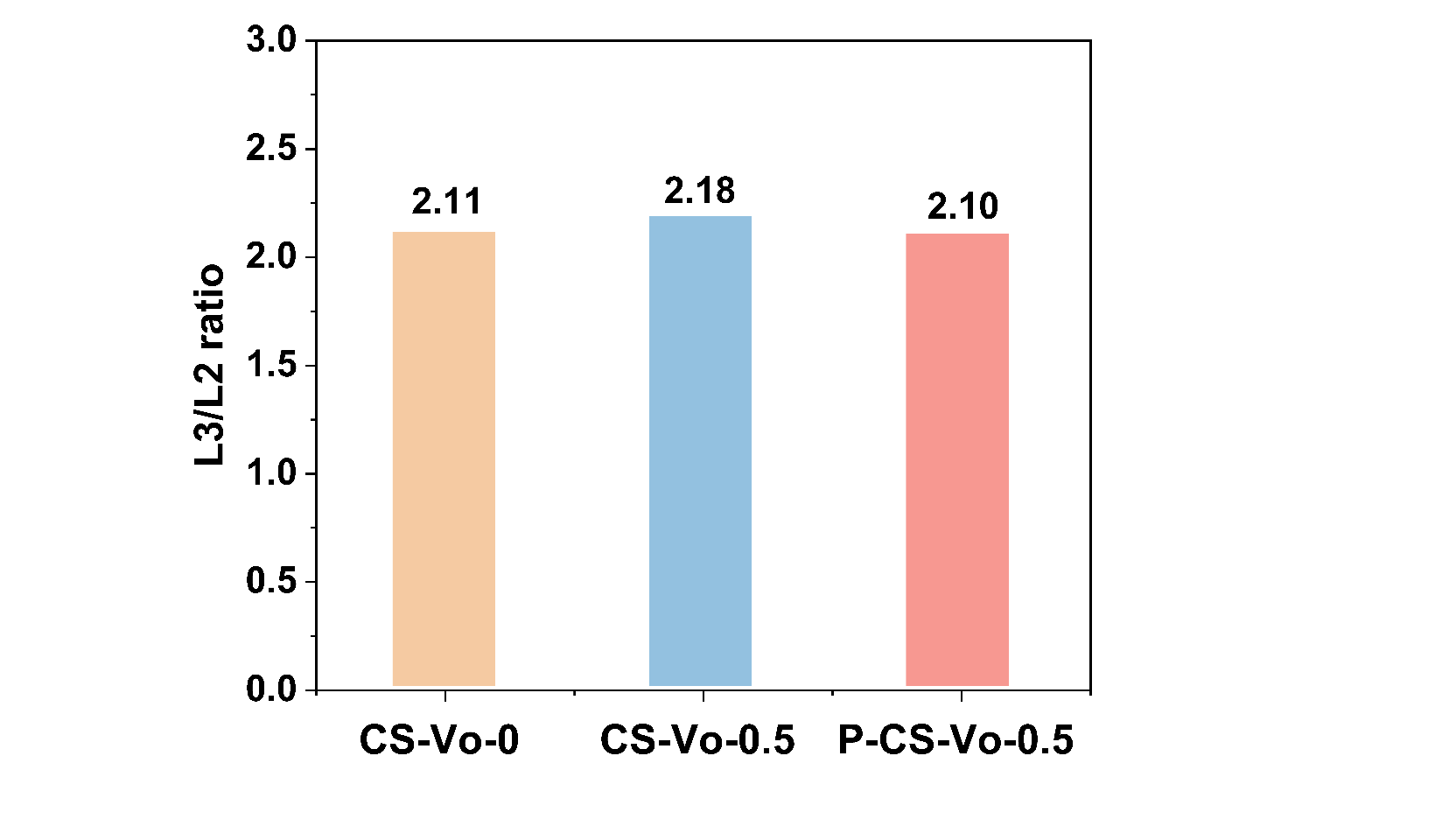


**Fig. S19** The L3/L2 ratio of Co L-edge.


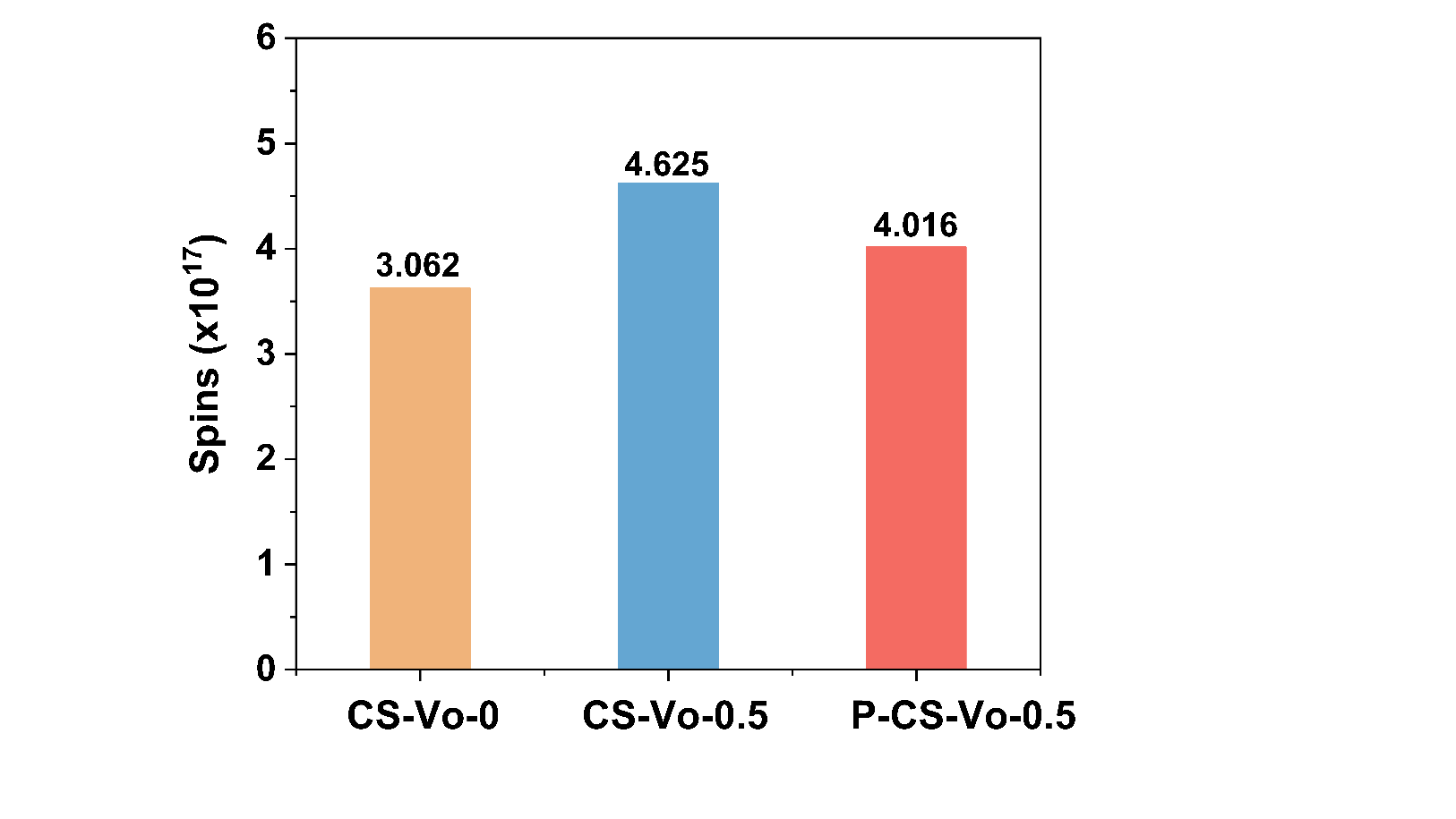


**Fig. S20** The corresponding vacancy concentration of different samples


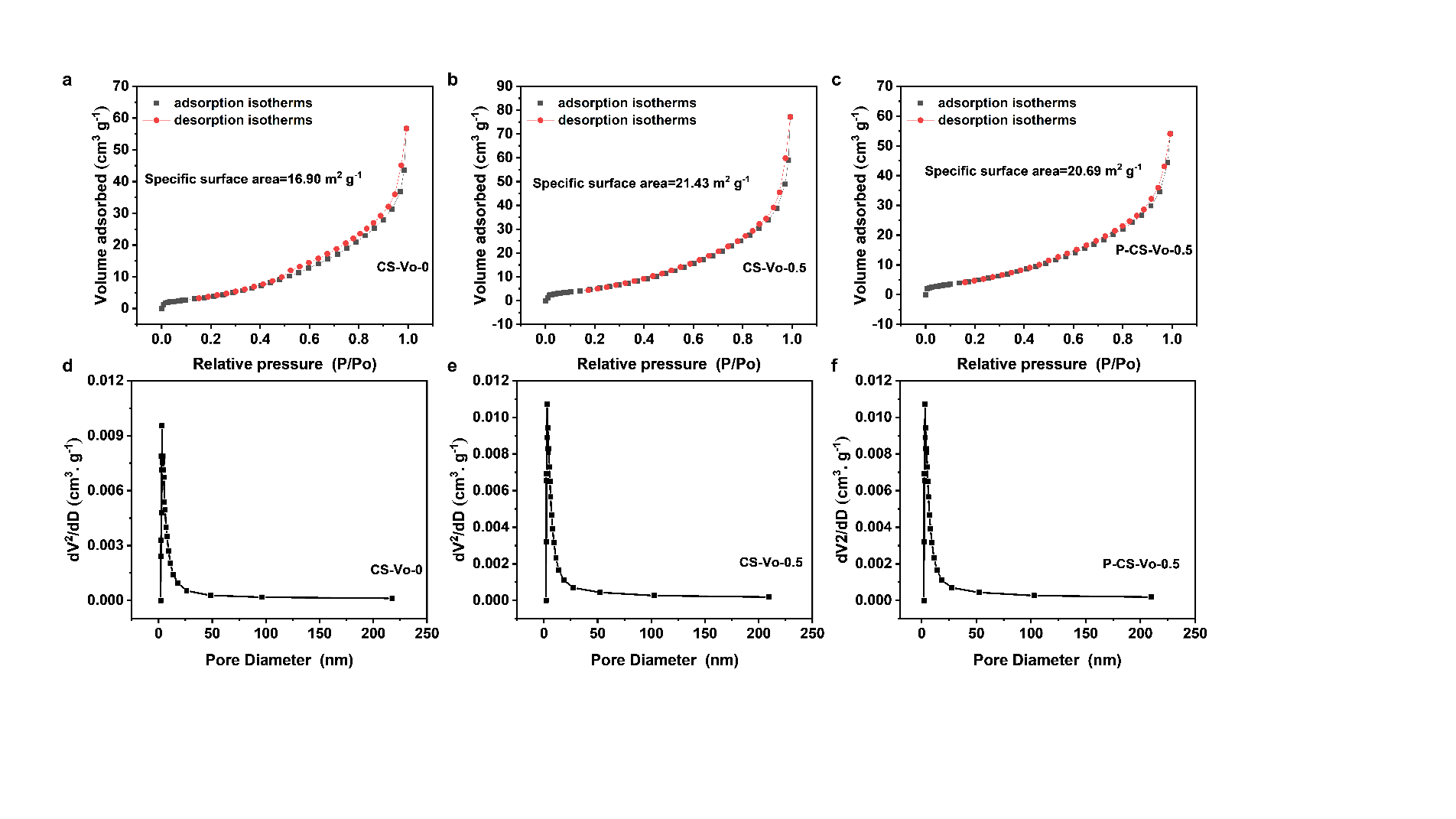


**Fig. S21** N_2_ adsorption-desorption isotherms and pore distribution of (**a, d**) CS-Vo-0, (**b, e**) CS-Vo-0.5 and (**c, f**) P-CS-Vo-0.5


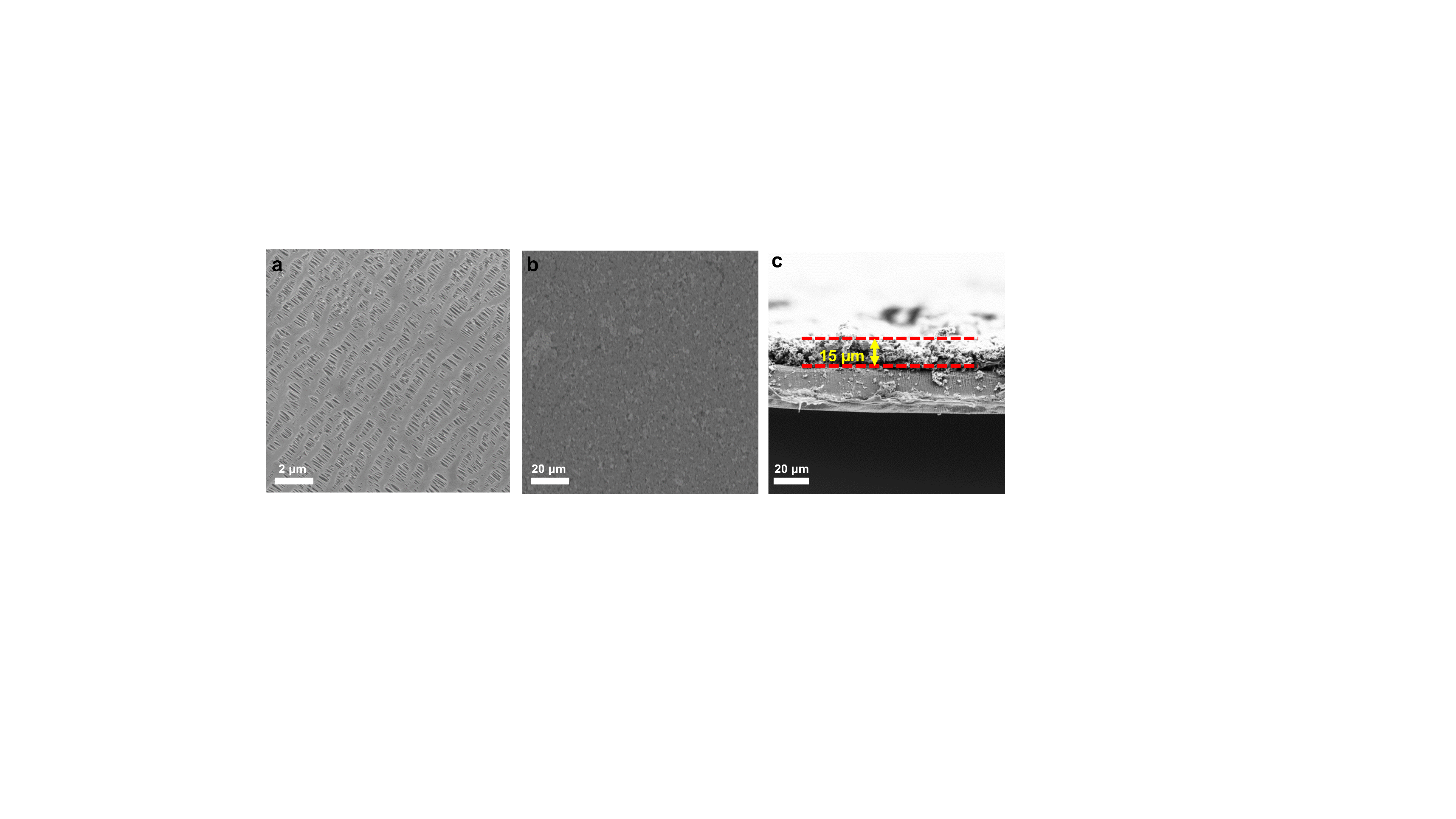


**Fig. S22** SEM image of (**a**) PP and (**b**) P-CS-Vo-0.5 separator, (**c**) the cross-sectional SEM image of P-CS-Vo-0.5 separator


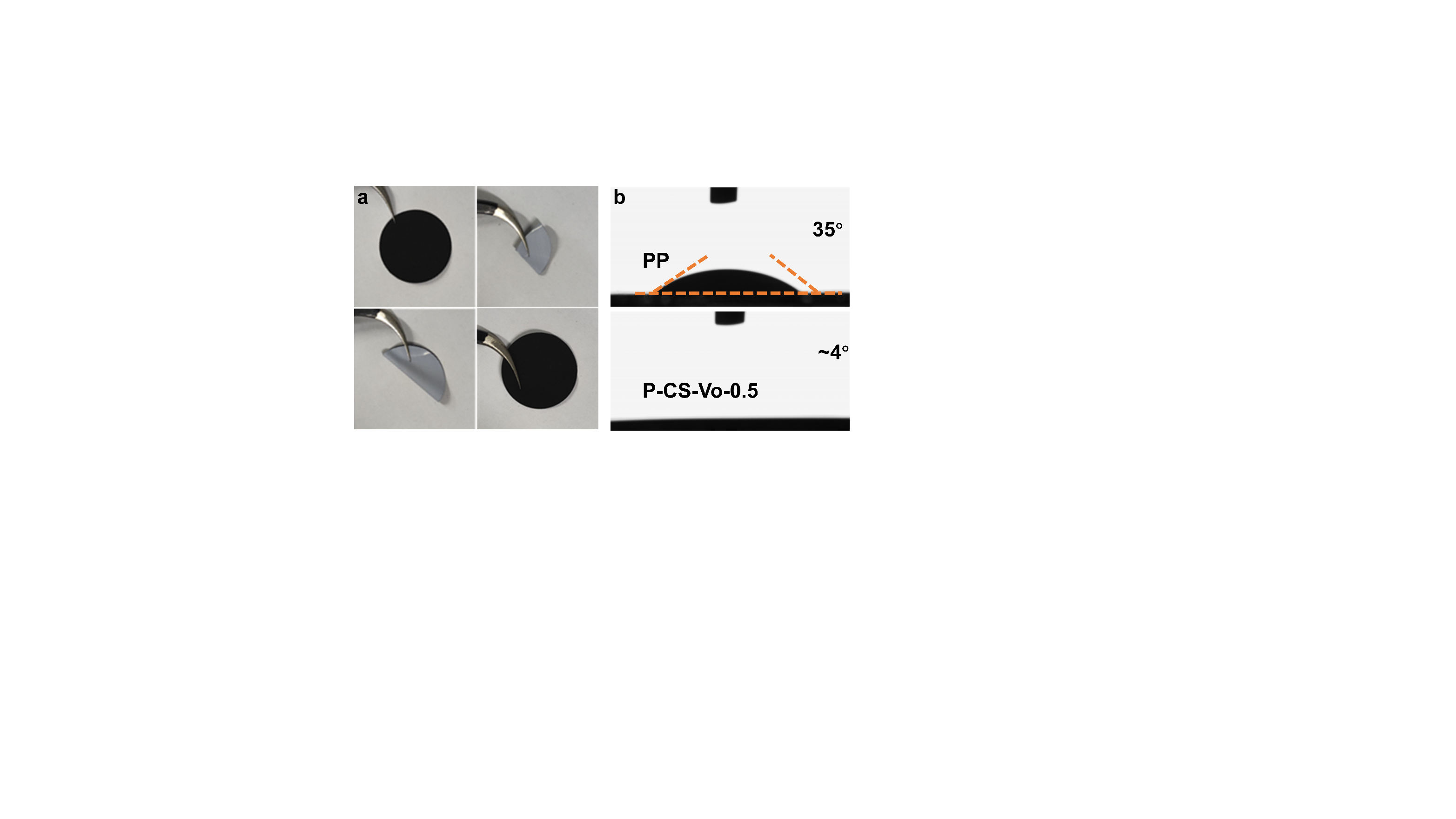


**Fig. S23** (**a**) The digital photo of P-CS-Vo-0.5 separator, (**b**) the contact angle between the electrolyte and different separators PP and P-CS-Vo-0.5


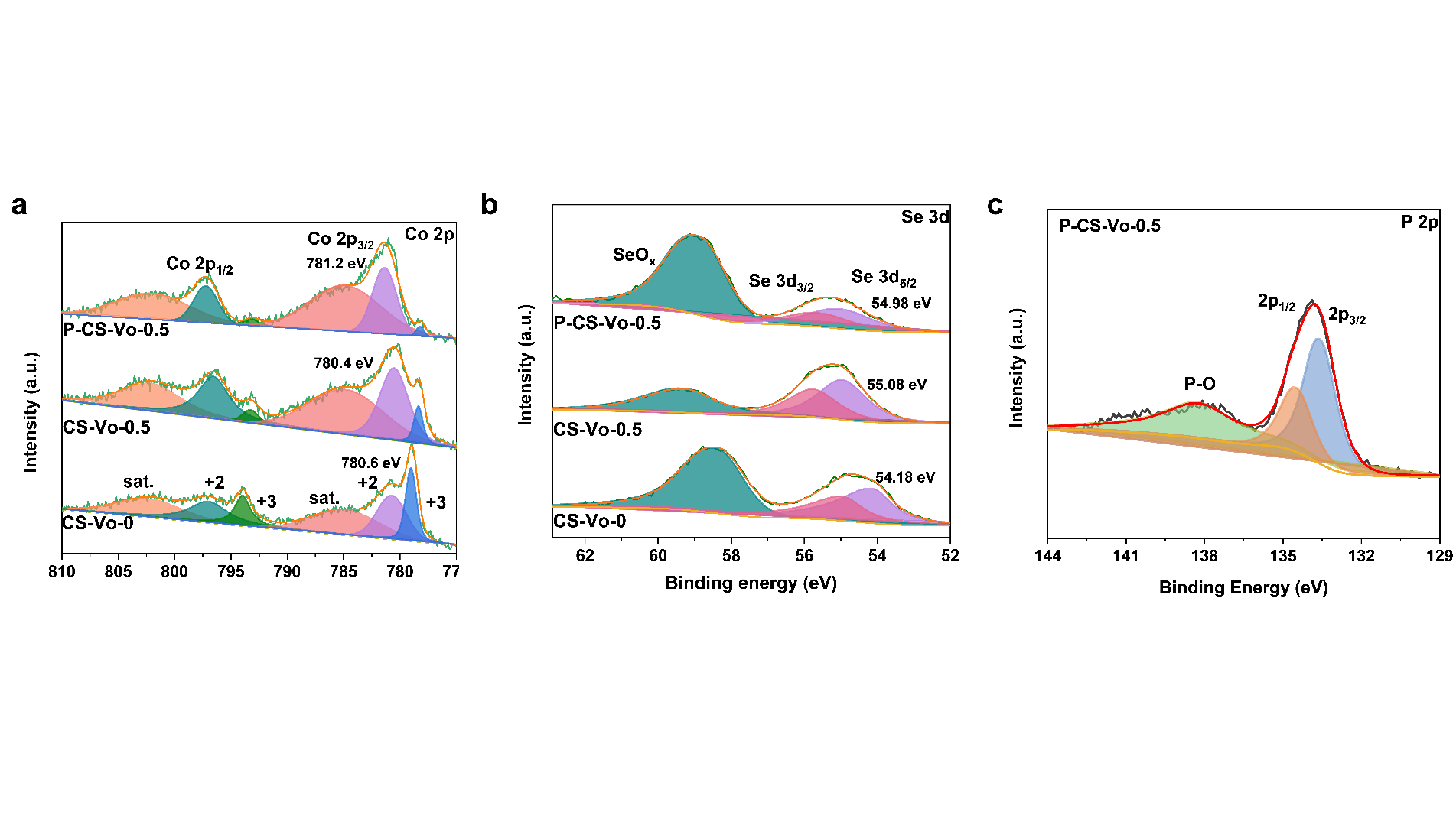


**Fig. S24** XPS of (**a**) Co 2p and (**b**) Se 3d (**c**) P 2p


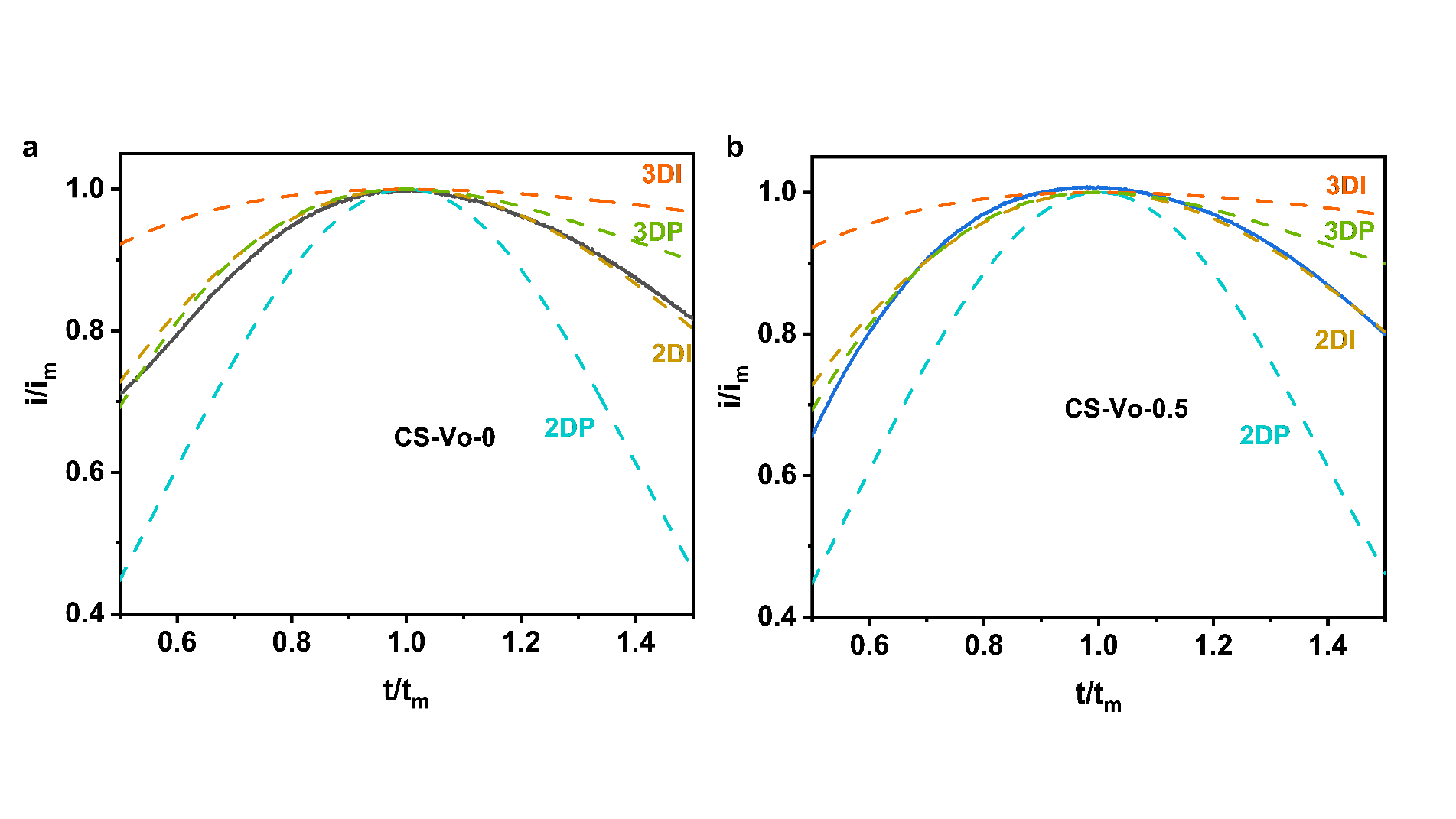


**Fig. S25** Corresponding dimensionless transient curves of (a) CS-Vo-0 and (b) CS-Vo-0.5


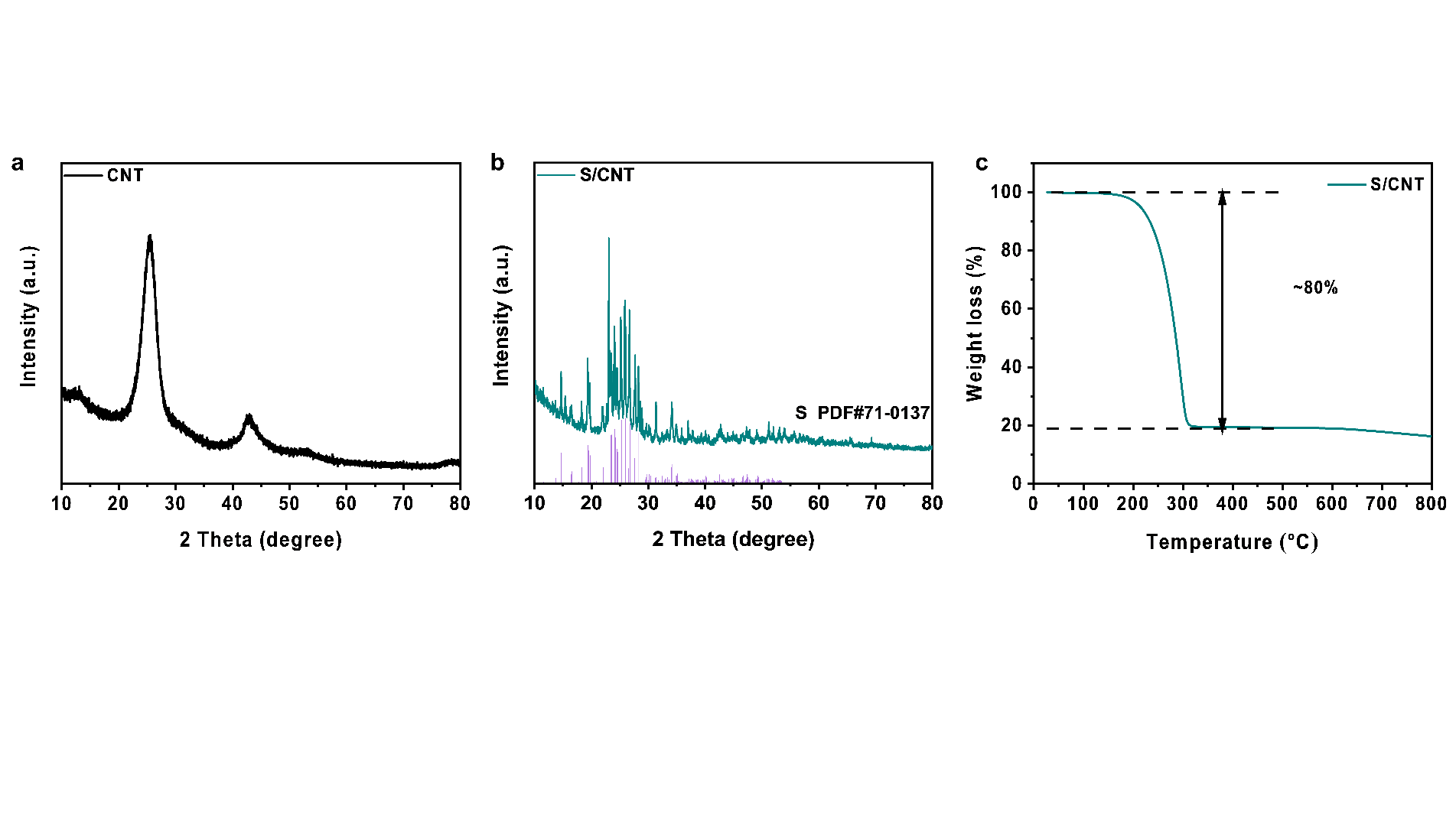


**Fig. S26** XRD patterns of (**a**) CNT and (**b**) S/CNT, (**c**) TG curves of S/CNT


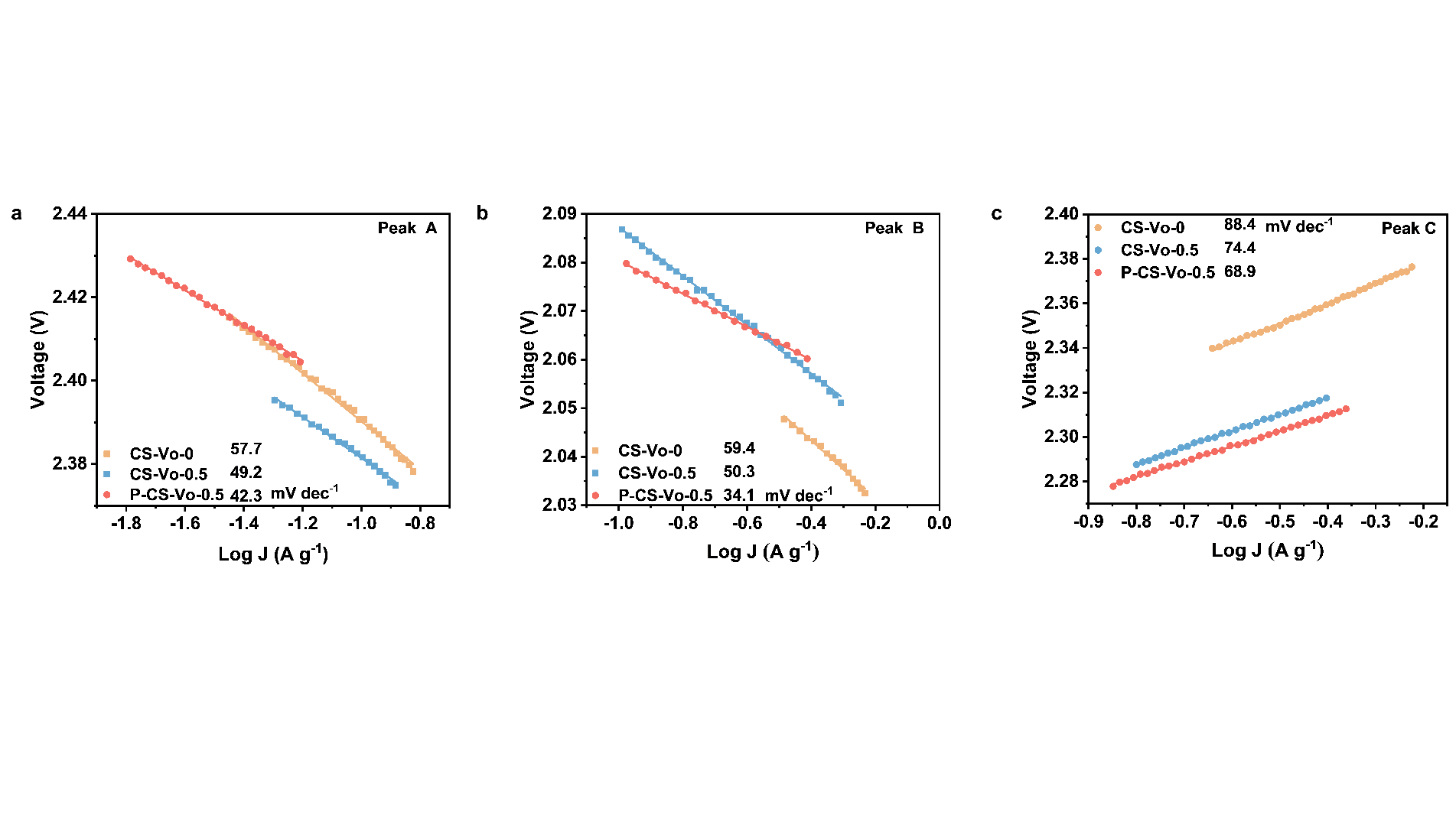


**Fig. S27** Tafel plots derived from CV curves calculated from the (**a**) Peak A, (**b**) Peak B, (**c**) Peak C


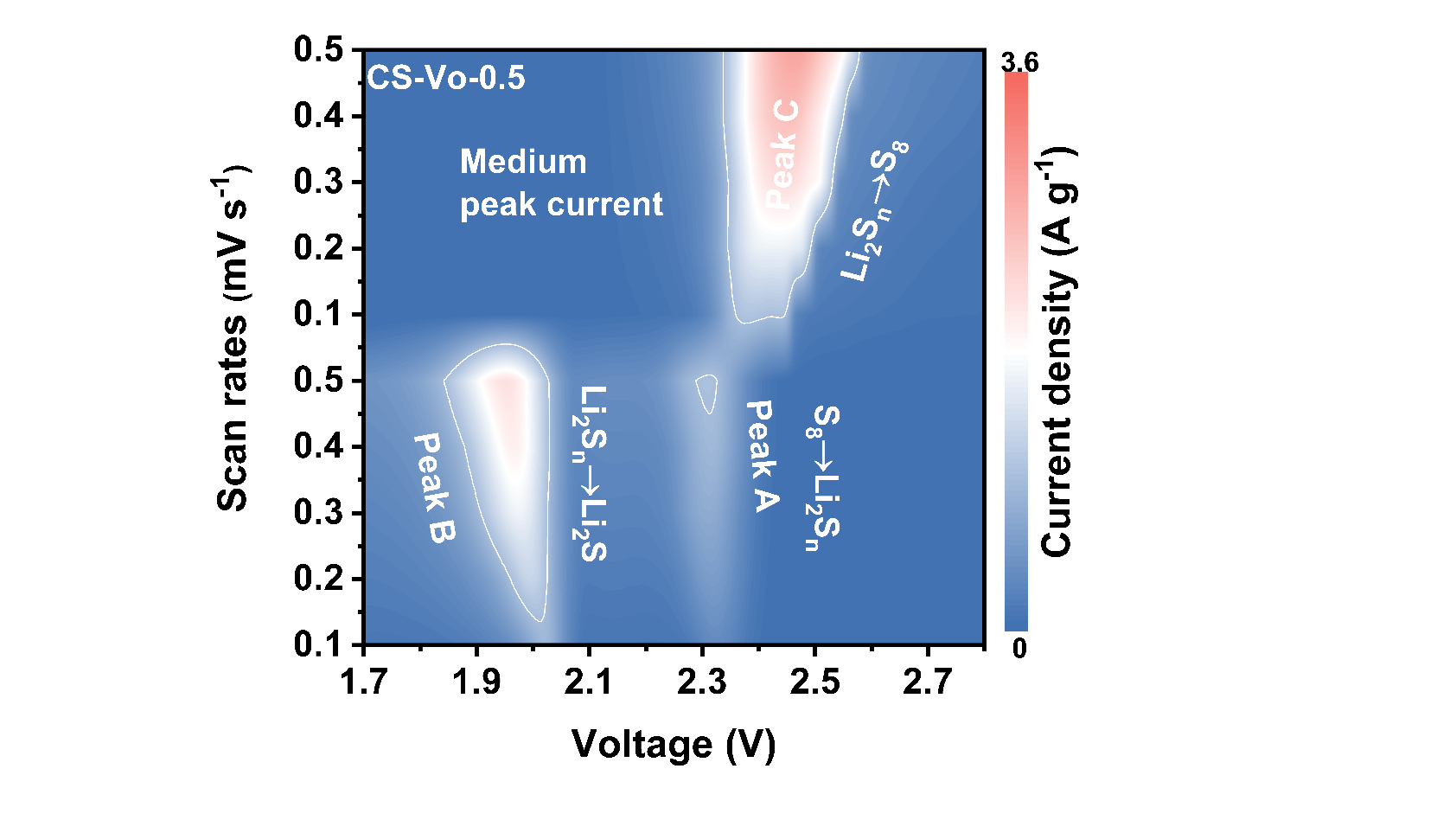


**Fig. S28** Contour plots of CV patterns for CS-Vo-0.5


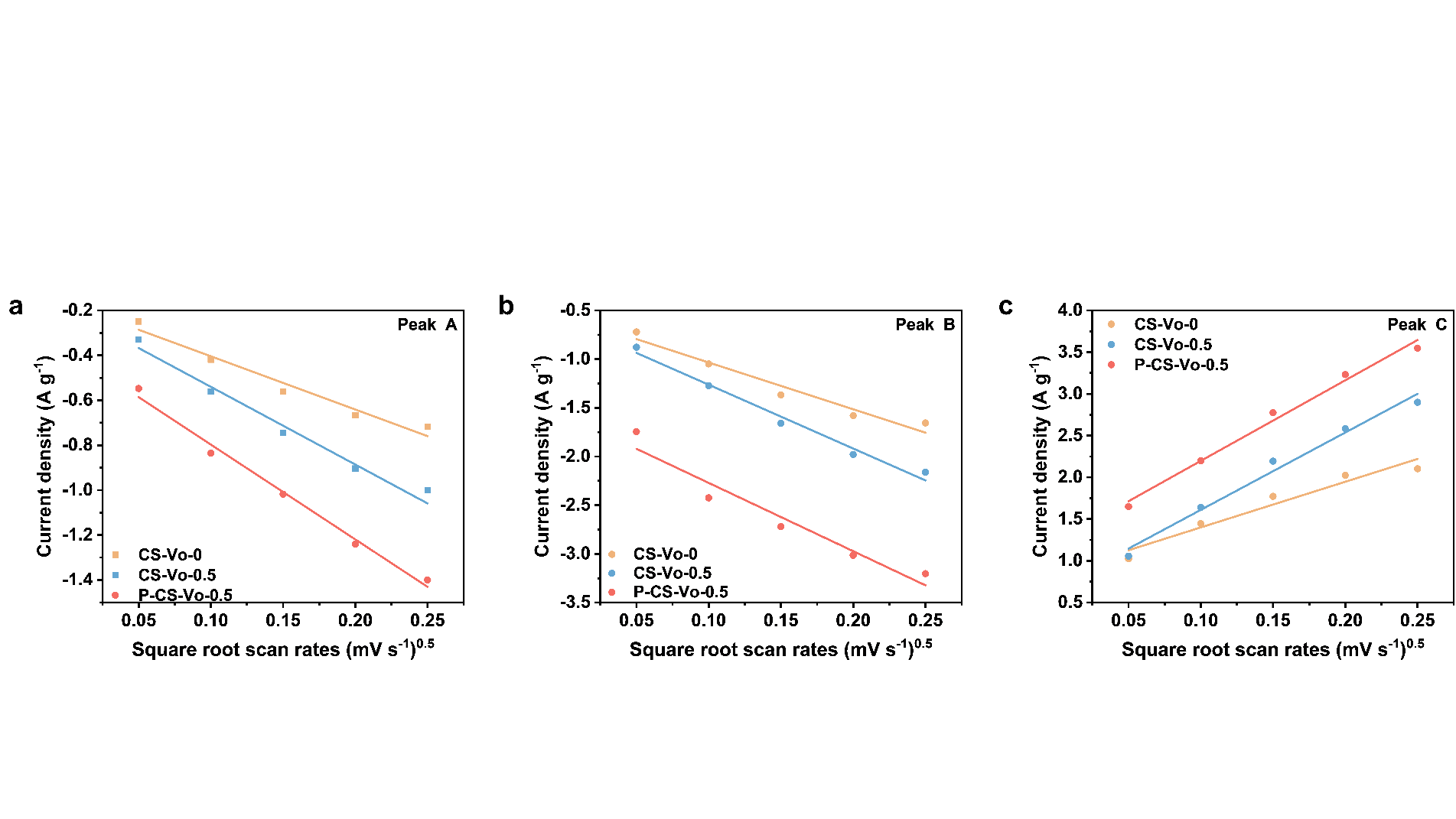


**Fig. S29** CV peak currents vs. square root of scan rate of (**a**) Peak A, (**b**) Peak B, and (**c**) Peak C with CS-Vo-0, CS-Vo-0.5 and P-CS-Vo-0.5


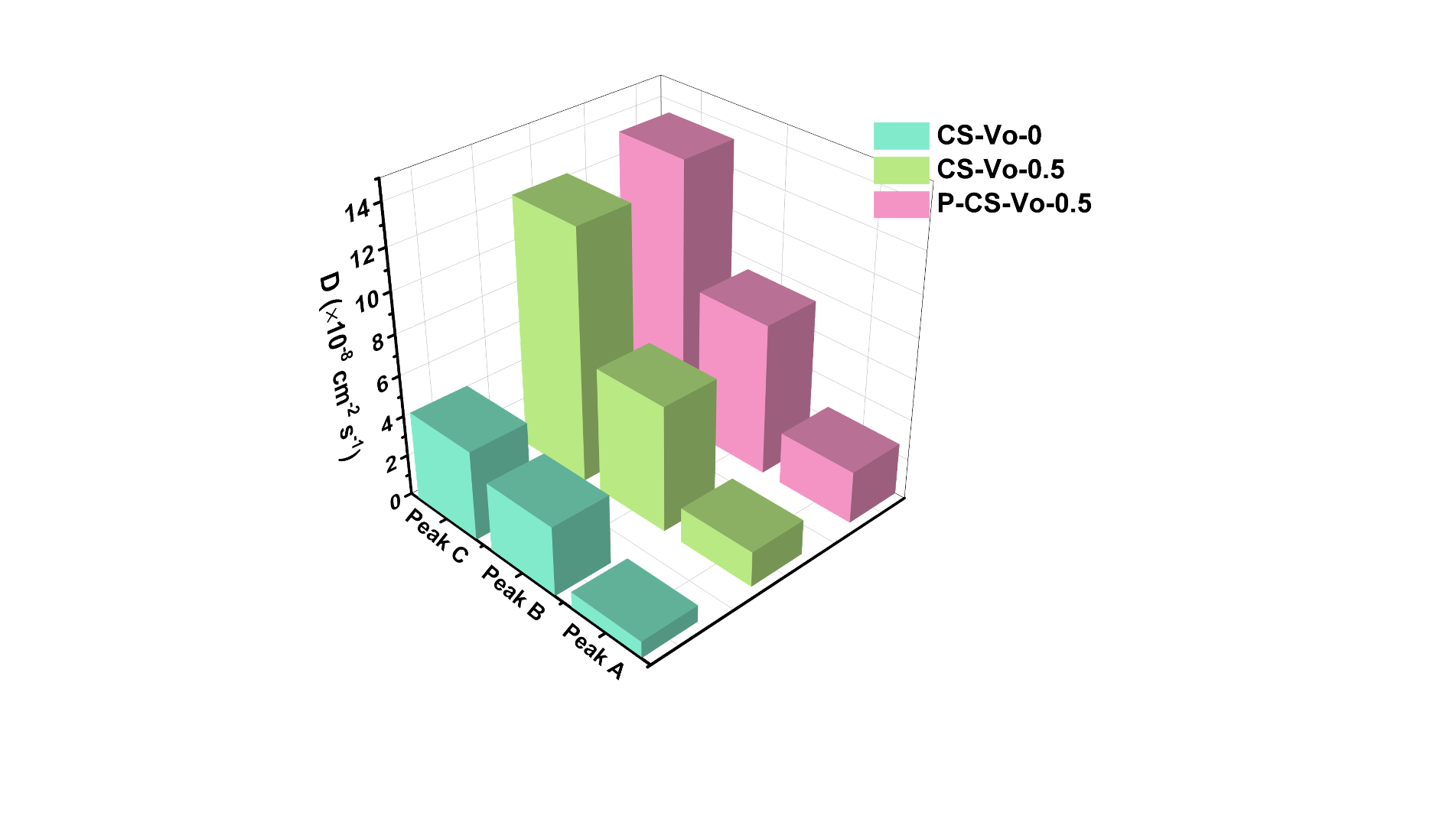


**Fig. S30** Lithium ion diffusion coefficient


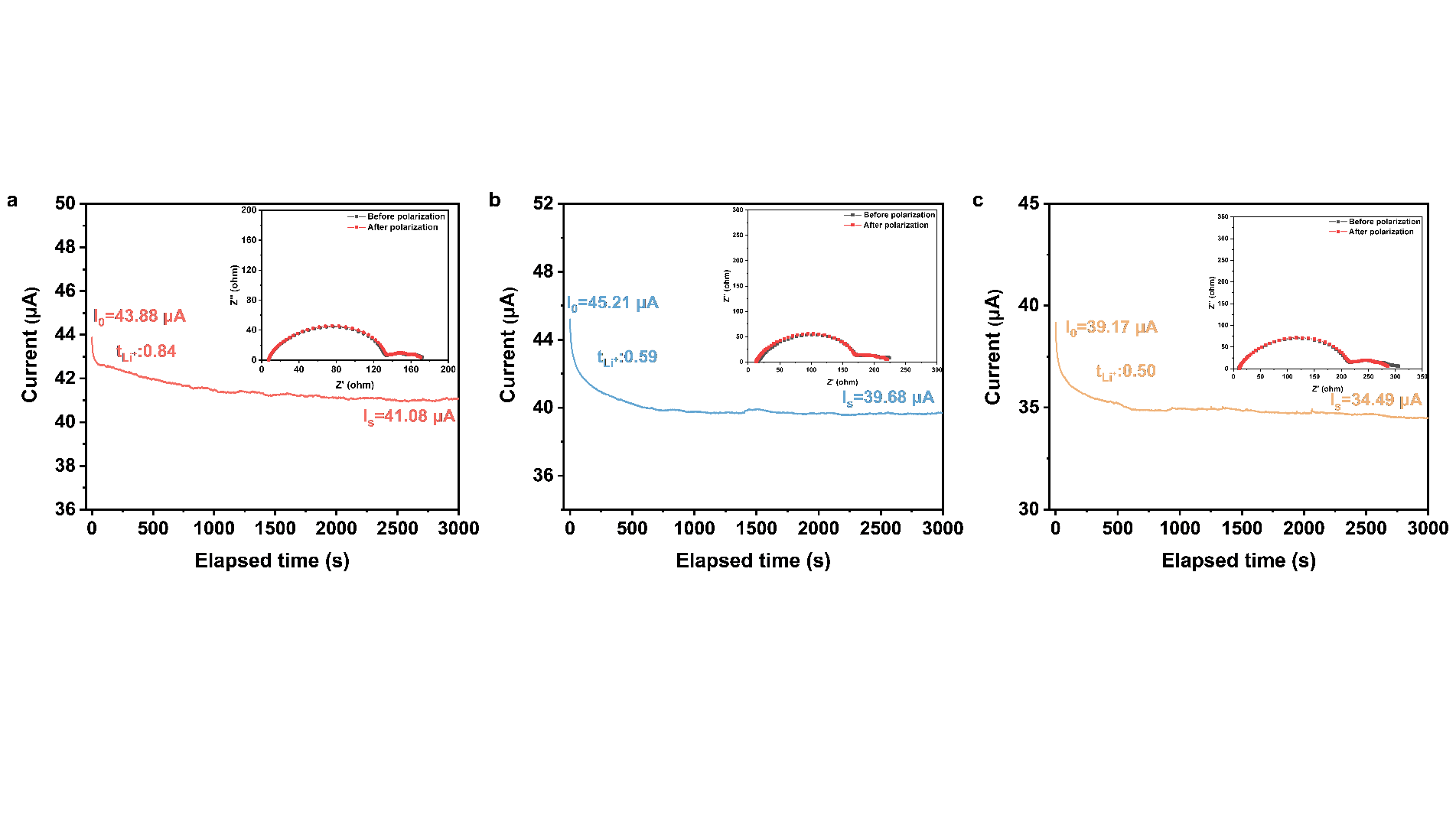


**Fig. S31** DC polarization curves of the (**a**) P-CS-Vo-0.5, (**b**) CS-Vo-0.5 and (**c**) CS-Vo-0; the inset shows the initial and steady-state Nyquist plots


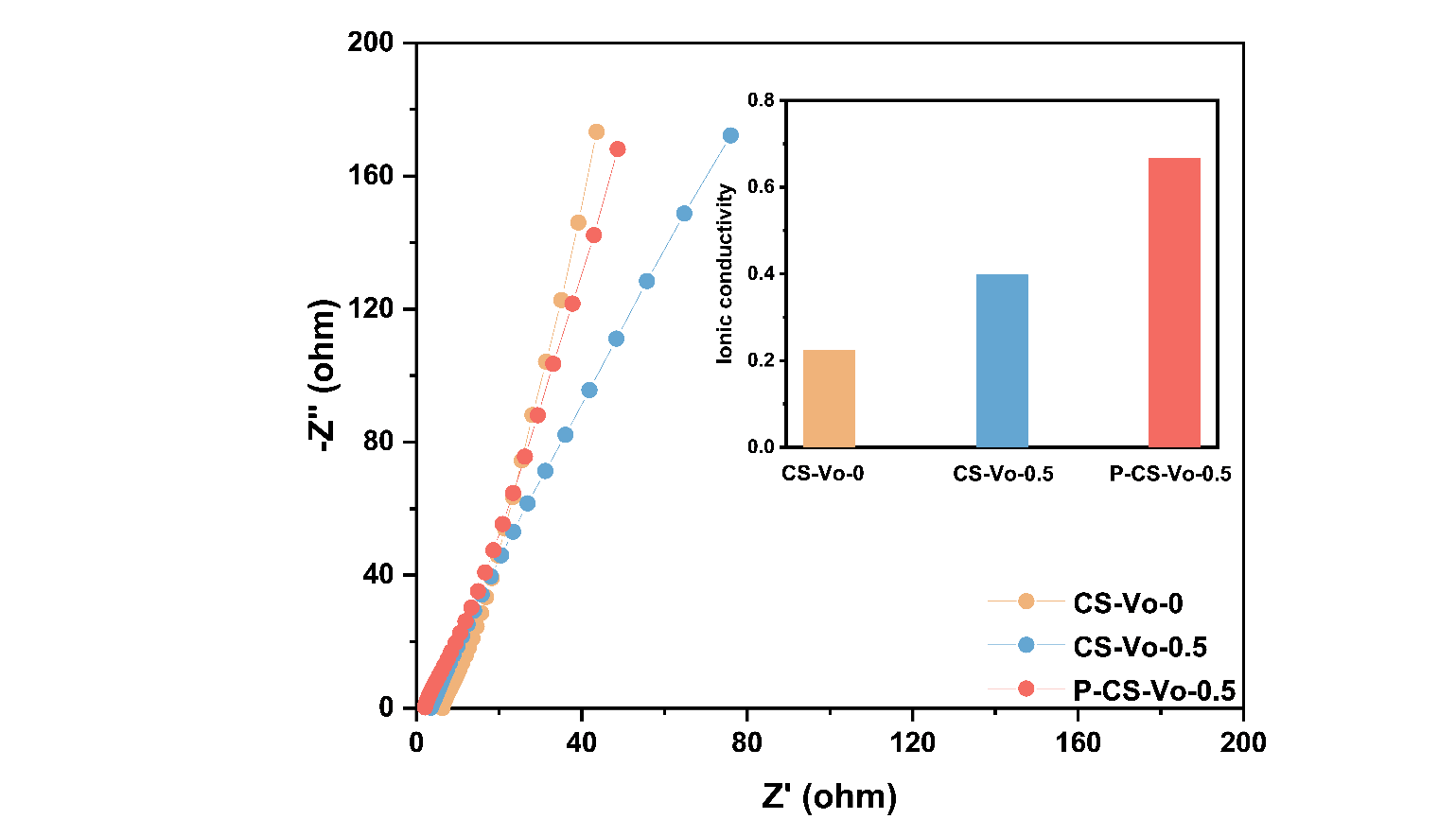


**Fig. S32** EIS curves and inset illustrates the lithium-ion conductivity of the batteries


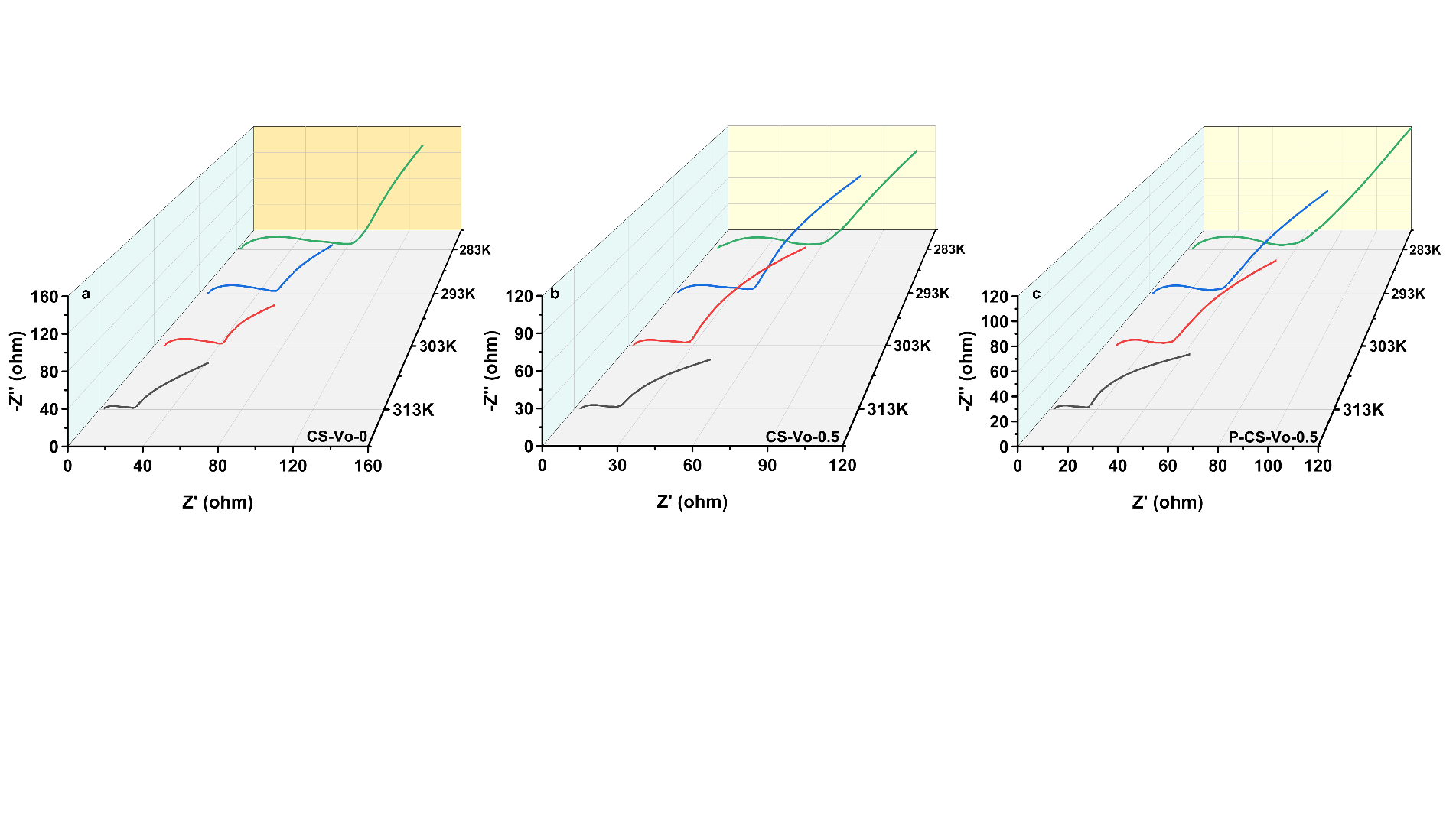


**Fig. S33** EIS measurements at various temperatures of Li-S batteries with different catalysts: (**a**) CS-Vo-0, (**b**) CS-Vo-0.5 and (**c**) P-CS-Vo-0.5


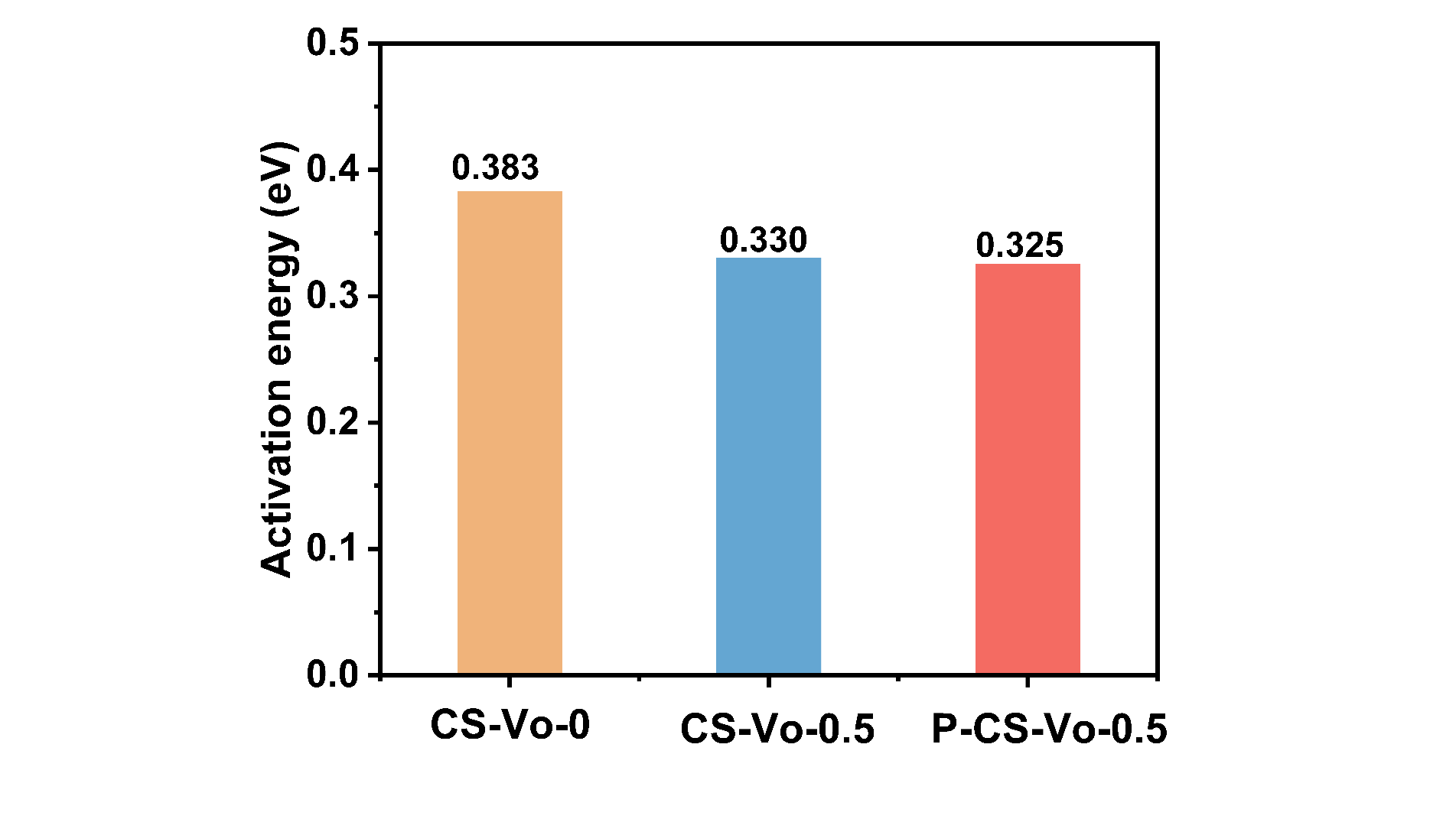


**Fig. S34** Activation energies for the step from Li_2_S_4_ to Li_2_S_2_ of the sulfur redox reactions


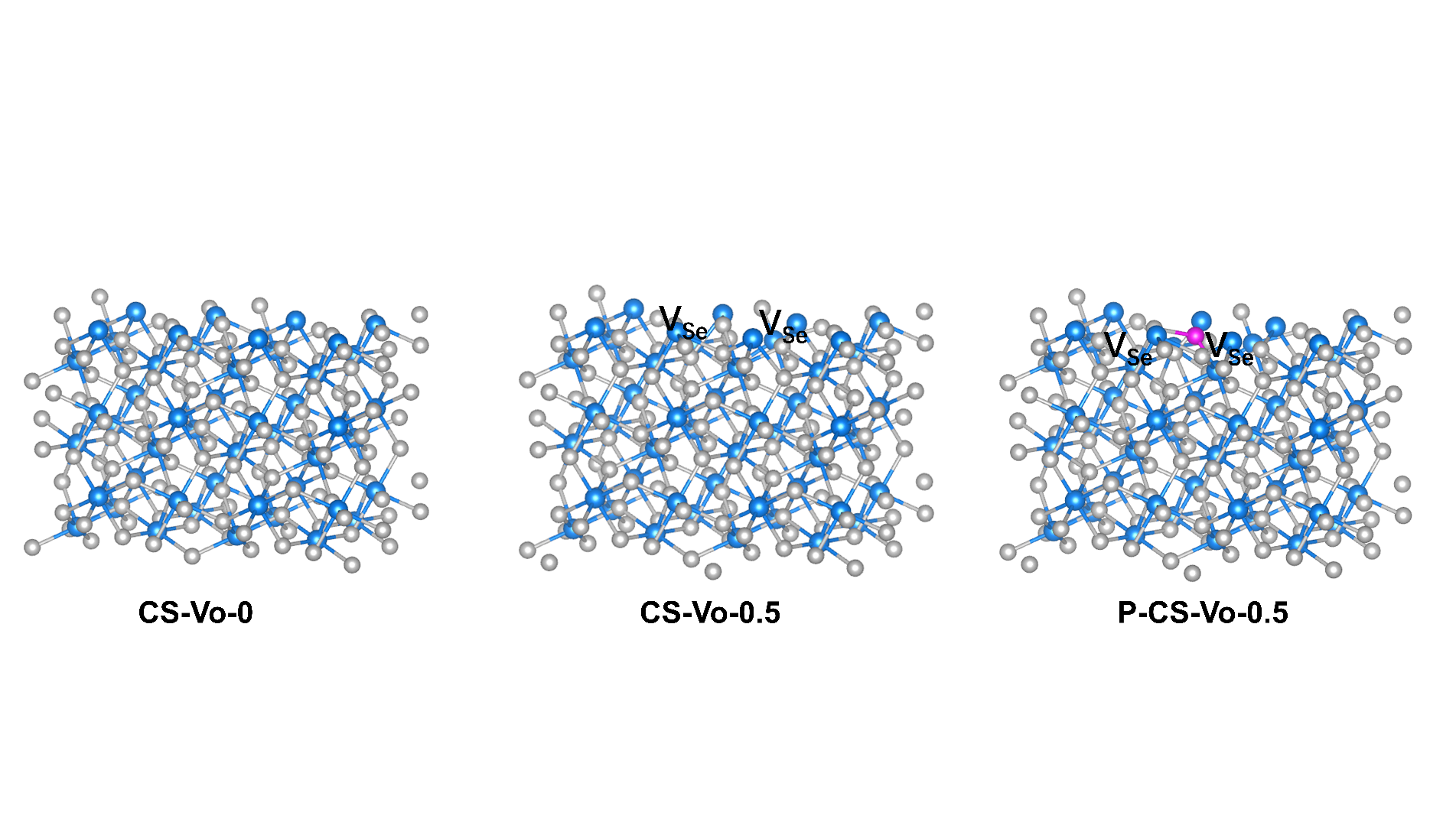


**Fig. S35 C**rystal structure of CS-Vo-0, CS-Vo-0.5 and P-CS-Vo-0.5


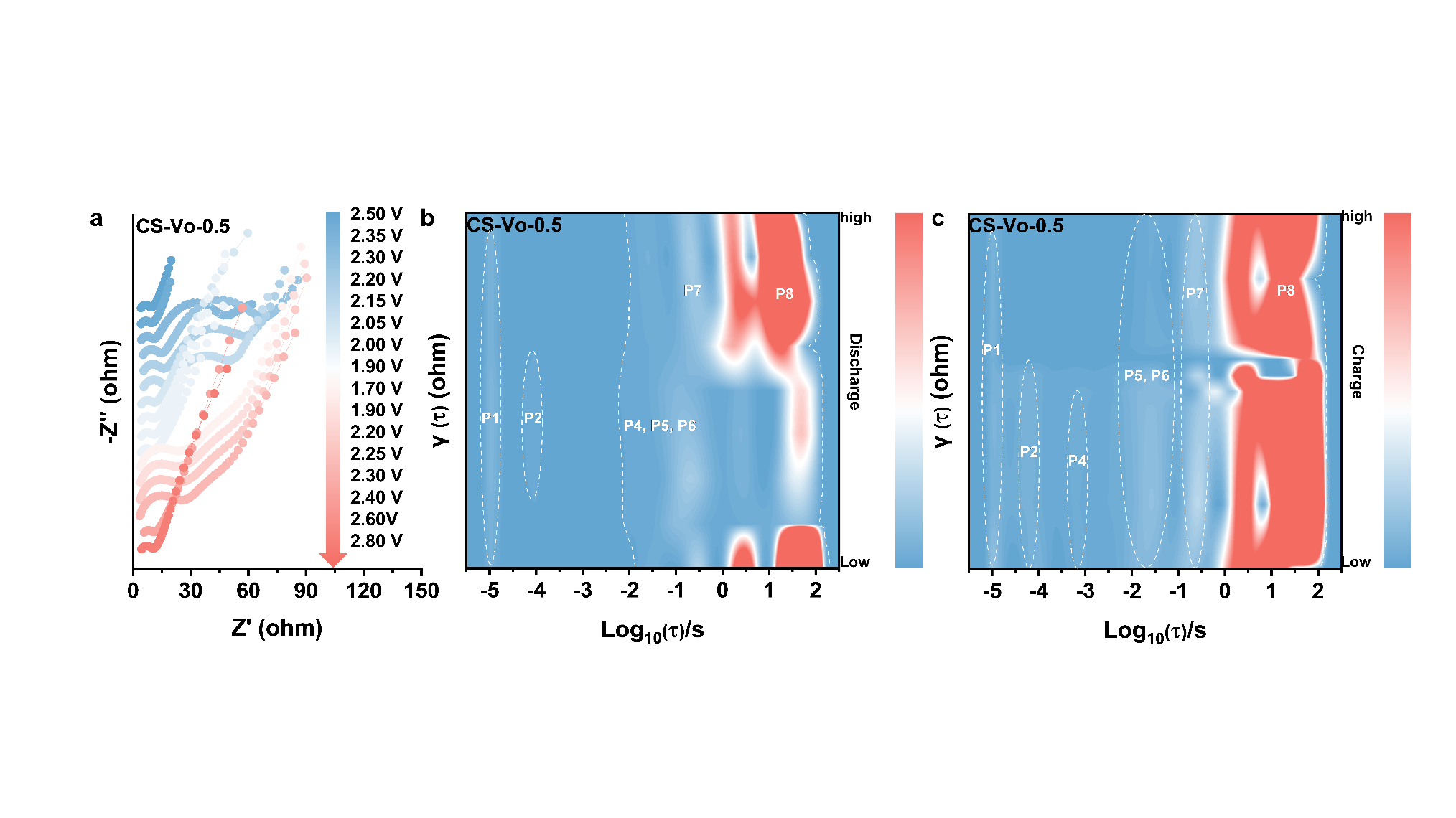


**Fig. S36** (**a**) *In situ* EIS Nyquist plots of battery with separator. DRT contour plots calculated from EIS measurements during (**b**) discharge and (**c**) charge process at different voltages of CS-Vo-0.5.


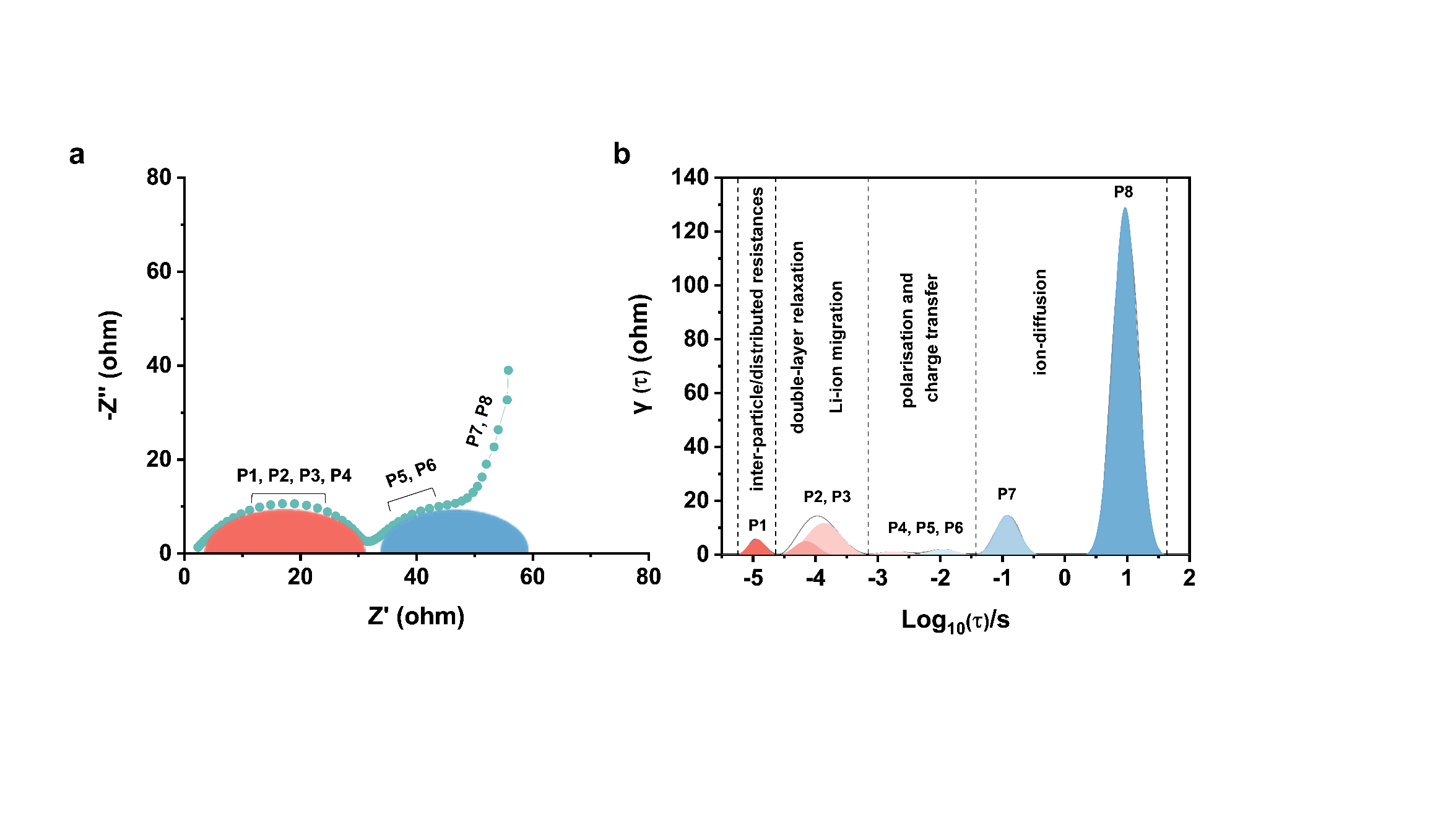


**Fig. S37** (**a**) Nyquist plot of P-CS-Vo-0.5 and CS; (**b**) DRT plot of the impedance data shown in (a)


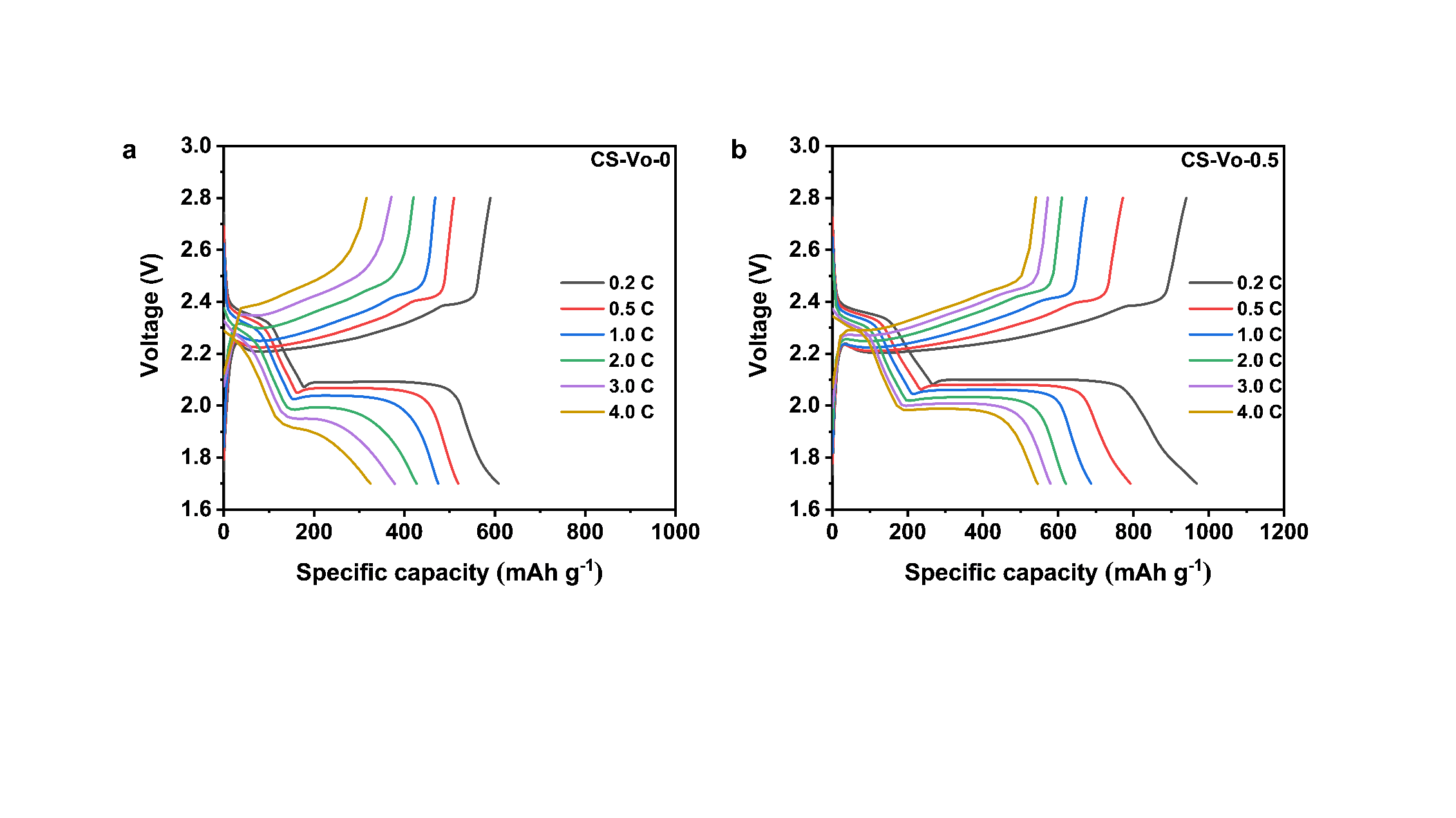


**Fig. S38** GCD curves of (**a**) CS-Vo-0 and (**b**) CS-Vo-0.5

**
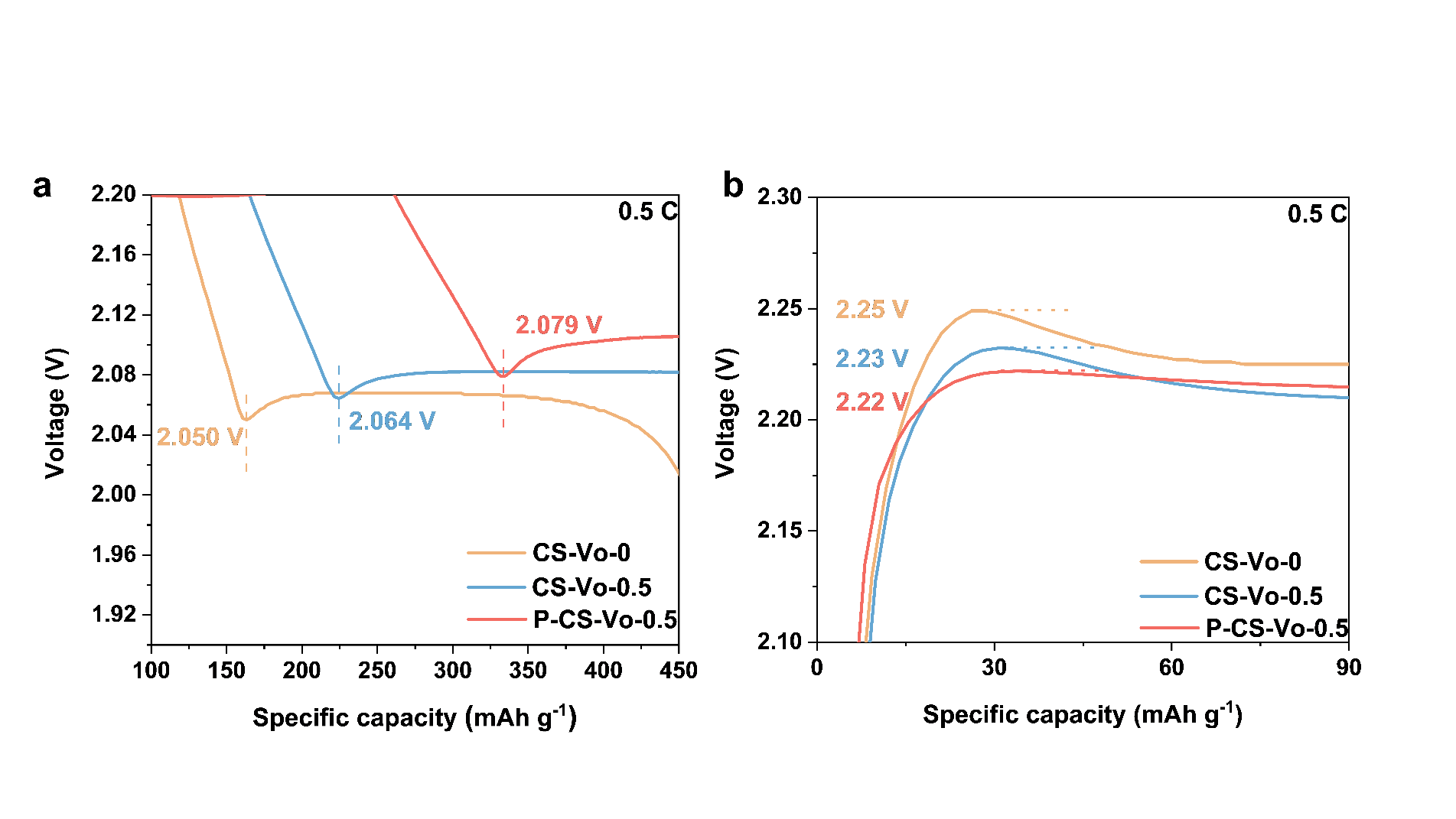
**

**Fig. S39** The corresponding magnified curves in the stages of (**a**) nucleation of Li_2_S_x_ and (**b**) decomposition of Li_2_S


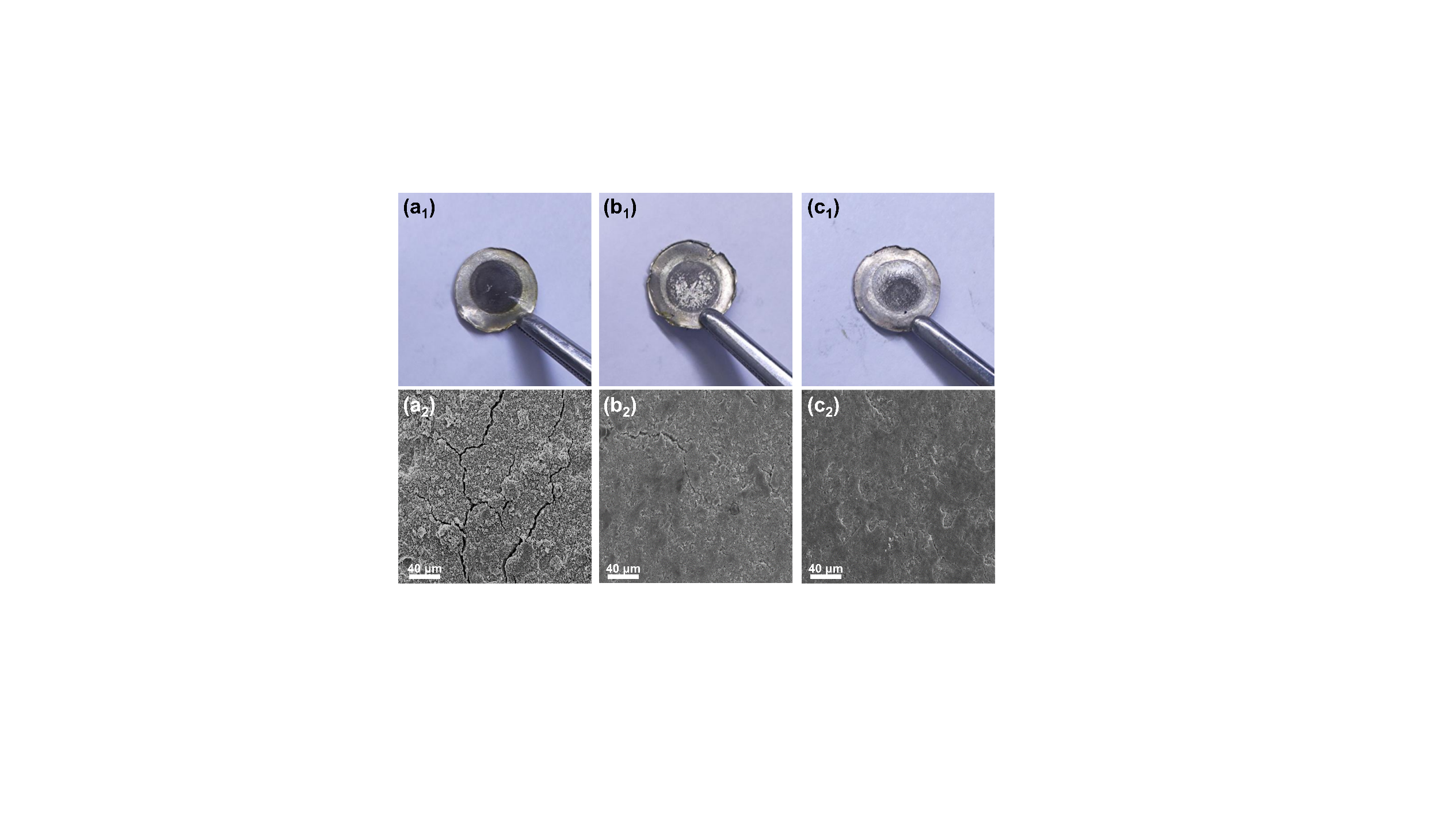


**Fig. S40** SEM images of the separator and digital photos of the lithium tablets after cycles of the (**a**) CS-Vo-0, (**b**) CS-Vo-0.5 and (**c**) P-CS-Vo-0.5


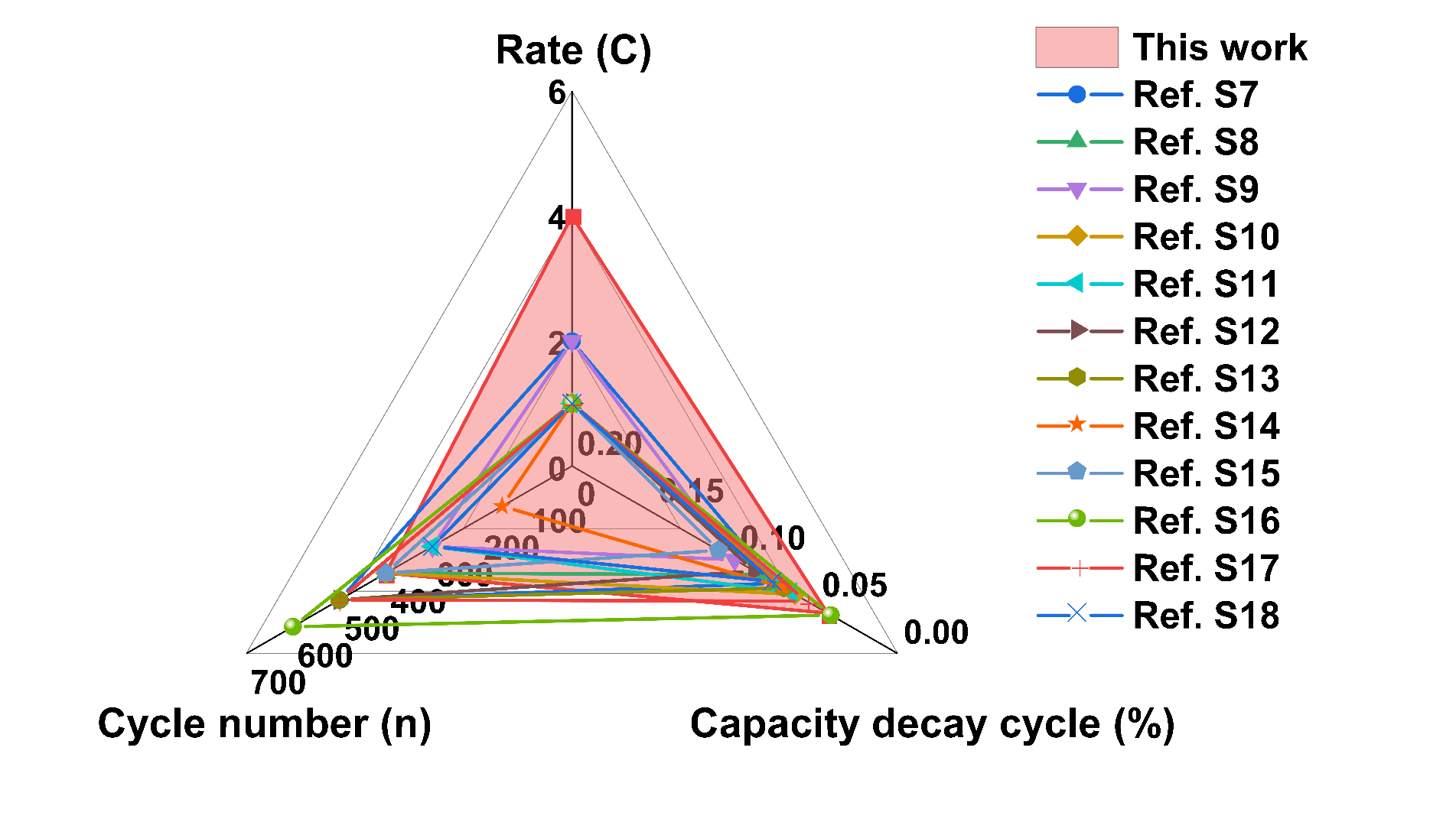


**Fig. S41** Comparison of electrochemical performance with the reported P-CS-Vo-0.5 and recent relevant literature

**Table S1** The elemental ratio of cobalt, selenium and phosphorus of the samples.

| **Sample** | **Co** | **Se** | **P** |
| --- | --- | --- | --- |
| **CS-Vo-0** | **1** | **1.92** | **/** |
| **CS-Vo-0.1** | **1** | **1.85** | **/** |
| **CS-Vo-0.5** | **1** | **1.72** | **/** |
| **CS-Vo-1** | **1** | **1.65** | **/** |
| **P-CS-Vo-0.5** | **1** | **1.70** | **0.10** |

**Supplementary References**

1. G. Kresse, J. Furthmüller, Efficiency of ab-initio total energy calculations for metals and semiconductors using a plane-wave basis set. Comput. Mater. Sci. **6**(1), 15–50 (1996). <https://doi.org/10.1016/0927-0256(96)00008-0>
2. G. Kresse, J. Furthmüller, Efficient iterative schemes for *ab initio* total-energy calculations using a plane-wave basis set. Phys. Rev. B Condens. Matter **54**(16), 11169–11186 (1996). <https://doi.org/10.1103/physrevb.54.11169>
3. R. Ouyang, W.-X. Li, First-principles study of the adsorption of Au atoms and Au2 and Au4 clusters on FeO/Pt(111). Phys. Rev. B **84**(16), 165403 (2011). <https://doi.org/10.1103/physrevb.84.165403>
4. G. Kresse, D. Joubert, From ultrasoft pseudopotentials to the projector augmented-wave method. Phys. Rev. B **59**(3), 1758–1775 (1999). <https://doi.org/10.1103/PhysRevB.59.1758>
5. P.E. Blöchl, Projector augmented-wave method. Phys. Rev. B 50(24), 17953–17979 (1994). <https://doi.org/10.1103/physrevb.50.17953>
6. V. Wang, N. Xu, J.-C. Liu, G. Tang, W.-T. Geng, VASPKIT: a user-friendly interface facilitating high-throughput computing and analysis using VASP code. Comput. Phys. Commun. **267**, 108033 (2021). <https://doi.org/10.1016/j.cpc.2021.108033>
7. J. Xia, W. Hua, L. Wang, Y. Sun, C. Geng et al., Boosting catalytic activity by seeding nanocatalysts onto interlayers to inhibit polysulfide shuttling in Li–S batteries. Adv. Funct. Mater. **31**(26), 2101980 (2021). <https://doi.org/10.1002/adfm.202101980>
8. L. Ma, Y. Zhang, C. Zhang, H. Zhu, S. Zhang et al., A separator modified by barium titanate with macroscopic polarization electric field for high-performance lithium–sulfur batteries. Nanoscale **15**(12), 5899–5908 (2023). <https://doi.org/10.1039/D3NR00263B>
9. D. He, J. Meng, X. Chen, Y. Liao, Z. Cheng et al., Ultrathin conductive interlayer with high-density antisite defects for advanced lithium–sulfur batteries. Adv. Funct. Mater. **31**(2), 2001201 (2021). <https://doi.org/10.1002/adfm.202001201>
10. L. Ma, Y. Wang, Z. Wang, J. Wang, Y. Cheng et al., Wide-temperature operation of lithium-sulfur batteries enabled by multi-branched vanadium nitride electrocatalyst. ACS Nano **17**(12), 11527–11536 (2023). <https://doi.org/10.1021/acsnano.3c01469>
11. N. Wang, B. Chen, K. Qin, E. Liu, C. Shi et al., Rational design of Co9S8/CoO heterostructures with well-defined interfaces for lithium sulfur batteries: a study of synergistic adsorption-electrocatalysis function. Nano Energy **60**, 332–339 (2019). <https://doi.org/10.1016/j.nanoen.2019.03.060>
12. Z. Gu, C. Cheng, T. Yan, G. Liu, J. Jiang et al., Synergistic effect of Co_3_Fe7 alloy and N-doped hollow carbon spheres with high activity and stability for high-performance lithium-sulfur batteries. Nano Energy **86**, 106111 (2021). <https://doi.org/10.1016/j.nanoen.2021.106111>
13. P. Wang, B. Xi, Z. Zhang, M. Huang, J. Feng et al., Atomic tungsten on graphene with unique coordination enabling kinetically boosted lithium–sulfur batteries. Angew. Chem. Int. Ed. **60**(28), 15563–15571 (2021). <https://doi.org/10.1002/anie.202104053>
14. M. Zhao, H.-J. Peng, B.-Q. Li, X. Chen, J. Xie et al., Electrochemical phase evolution of metal-based pre-catalysts for high-rate polysulfide conversion. Angew. Chem. Int. Ed. **59**(23), 9011–9017 (2020). <https://doi.org/10.1002/anie.202003136>
15. L. Luo, X. Qin, J. Wu, G. Liang, Q. Li et al., An interwoven MoO_3_@CNT scaffold interlayer for high-performance lithium–sulfur batteries. J. Mater. Chem. A **6**(18), 8612–8619 (2018). <https://doi.org/10.1039/C8TA01726C>
16. L. Chen, Y. Sun, X. Wei, L. Song, G. Tao et al., Dual-functional V2C MXene assembly in facilitating sulfur evolution kinetics and Li-ion sieving toward practical lithium–sulfur batteries. Adv. Mater. **35**(26), 2300771 (2023). <https://doi.org/10.1002/adma.202300771>
17. Z.-D. Pan, Z.-L. Wang, X.-Y. Rao, X. Liu, Y. Zhang, Synergistic effect of oxygen-deficient Ni_3_V_2_O_8_@carbon nanotubes-modified separator for advanced lithium–sulfur batteries. Rare Met. **44**(3), 1632–1648 (2025). <https://doi.org/10.1007/s12598-024-03180-z>
18. J. Sivaraj, B. Dasari, K. Ramesha, Cobalt vanadate (Co_3_V_2_O_8_) hollow microspheres as a polysulfide adsorption and conversion catalyst for Li–S batteries. Energy Fuels **37**(13), 9672–9681 (2023). <https://doi.org/10.1021/acs.energyfuels.3c01153>
